# Hand classification of fMRI ICA noise components

**DOI:** 10.1016/j.neuroimage.2016.12.036

**Published:** 2017-07-01

**Authors:** Ludovica Griffanti, Gwenaëlle Douaud, Janine Bijsterbosch, Stefania Evangelisti, Fidel Alfaro-Almagro, Matthew F. Glasser, Eugene P. Duff, Sean Fitzgibbon, Robert Westphal, Davide Carone, Christian F. Beckmann, Stephen M. Smith

**Affiliations:** a Centre for the functional MRI of the Brain (FMRIB), University of Oxford, United Kingdom; b Functional MR Unit, Policlinico S. Orsola - Malpighi, Bologna, Italy - Department of Biomedical and Neuromotor Sciences (DIBINEM), University of Bologna, Bologna, Italy; c Washington University School of Medicine, Washington University, St. Louis, MO, USA; d Department of Psychiatry, University of Oxford, United Kingdom; e Acute Stroke Programme, Radcliffe Department of Medicine, University of Oxford, Oxford, United Kingdom; f Laboratory of Experimental Stroke Research, Department of Surgery and Translational Medicine, University of Milano Bicocca, Milan Center of Neuroscience, Monza, Italy; g Department of Cognitve Neuroscience, Radboudumc and Donders Institute for Brain, Cognition and Behaviour, Radboud University, Nijmegen, The Netherlands

## Abstract

We present a practical “how-to” guide to help determine whether single-subject fMRI independent components (ICs) characterise structured noise or not. Manual identification of signal and noise after ICA decomposition is required for efficient data denoising: to train supervised algorithms, to check the results of unsupervised ones or to manually clean the data. In this paper we describe the main spatial and temporal features of ICs and provide general guidelines on how to evaluate these. Examples of signal and noise components are provided from a wide range of datasets (3T data, including examples from the UK Biobank and the Human Connectome Project, and 7T data), together with practical guidelines for their identification. Finally, we discuss how the data quality, data type and preprocessing can influence the characteristics of the ICs and present examples of particularly challenging datasets.

## 1 Introduction

Spatial Independent Component Analysis (ICA) has proven to be a powerful tool for blind source separation of fMRI data (Beckmann and Smith, 2004, Hyvarinen, 1999, McKeown et al., 1998) into 3D spatial maps and 1D time courses. At the single subject level, ICA is increasingly often being used in the context of artefact removal (also called ‘data denoising’ or ‘data cleanup’), for its ability to separating neural-related signal from different sources of noise (Beckmann, 2012, Murphy et al., 2013).

Although fMRI data contain both structured and stochastic noise, in the context of ICA-based denoising the term *noise* refers to just the structured noise, given that ICA decomposition aims at un-mixing the data into non-Gaussian sources. Therefore, a noise component (N-IC) is defined as a component (time series and associated spatial map) that characterizes a noise/artefact effect. For ICA and any other linear decomposition technique, this necessitates that structured noise can be described in a linear fashion. However, while this may seem a significant restriction, it is the case that even non-linear effects may be approximately described by a superposition of linear effects.

The information represented in the ICA decomposition is used to detect N-ICs and to reduce the negative effect of artefacts on the analyses in various possible ways (Beckmann, 2012): (i) using the information from the spatial maps to remove certain voxels from further analysis; (ii) using the information from the time series to identify certain points in time that require attention (e.g. in the case of slice dropouts, the affected scans can either be excluded from further analysis or the intensity value at a time point can be adjusted to the mean intensity of the volumes acquired before and after the dropout occurred); (iii) regressing out the N-ICs related time courses from the original data (combining spatial maps with their associated time series to form an estimate of the noise in the data, and subtract it from the original data, see Griffanti et al. (2014) for details); (iv) reconstructing the data from the S-ICs (combining spatial maps with their associated time series and sum them together) (Perlbarg et al., 2007). Between the latter two approaches, regressing out the noise from the data is the most promising approach, as, unlike the reconstruction approach, it makes no assumptions about the signals of interest and therefore can be combined with later ‘null hypothesis’ testing, e.g. a classical GLM analysis (Beckmann, 2012). That option also allows for the later identification of weaker components in the data that lie in the stochastic part of the data for single-dataset ICA decompositions, but which may be identified from group-level ICA.

Independently from the denoising method adopted, the biggest challenge, and main focus of this paper, remains the identification of the N-IC. Several approaches have been proposed to achieve this aim, exploiting the fact that signal components (S-IC) and noise components (N-IC) differ in terms of spatial, temporal and (temporal) spectral characteristics.

Some methods, for example, classify ICs using task-paradigm timing information (Calhoun et al., 2005, Kochiyama et al., 2005, Thomas et al., 2002). However, such approaches can only correctly classify noise components in cases where there is little time series correlation between the paradigm and the IC time course. As such, stimulus correlated head motion effects captured within IC components will be challenging.

ICA-based cleaning is especially useful in resting state fMRI (rfMRI) data, where there is no a-priori information about the signal of interest.

A method that uses the temporal information without task-related information on single-echo data has been proposed by Thomas and colleagues (Thomas et al., 2002). This approach identifies (random or structured) noise components using an unsupervised algorithm that examines the Fourier decomposition of the time series. Perlbarg et al. (2007) used manually defined regions of interest (ROIs) to define typical time courses of structured noise in fMRI data, which were used as regressors for the BOLD signals, while Rummel et al. (2013) proposed a fully automatic time course-based filtering procedure to detect artefacts in the ICA.

The multi-echo method proposed by Kundu et al. (2012) differentiates BOLD-like functional network components from non-BOLD-like components related to motion, pulsatility, and other nuisance effects based on TE-dependence. This was found to be a robust method, although the technique requires a multi-echo acquisition sequence and cannot be applied to typical conventional single-echo fMRI data. Additionally there are limitations to the currently achievable spatial and temporal resolution of multi-echo fMRI data, because, for a given TR, increasing resolution competes with acquiring more TEs (Kundu et al., 2012).

Other approaches instead mainly rely on spatial information. Calhoun et al. (2008) use a brain atlas to aid sorting ICs, while Sui et al. (2009) employ purely spatial criteria to automatically classify ICs obtained from coefficient-constrained ICA that contain no time-domain information.

Among the methods exploiting both spatial and temporal information, some approaches focus on a specific category of artefacts. For example, the approaches proposed by Perlbarg et al. (2007) and Beall and Lowe (2007) focus on the identification of physiological noise (cardiac and respiratory fluctuations). The former, CORSICA, uses both spatial and temporal patterns to categorize ICs into noise and signal, while the second estimates those fluctuations from the rfMRI data with temporal ICA and generated spatial weight matrices applicable to other resting state data. ICA-AROMA, developed by Pruim et al., 2015a, Pruim et al., 2015b, focuses instead on the identification and removal of artefacts due to head motion using four spatial and temporal features. From a complementary perspective, Storti et al. (2013), utilizes both spatial and temporal information with the aim to identify signal components (RSNs), rather than artefacts.

Finally, another set of methods aims at identifying and removing a wider set of artefacts. De Martino et al. (2007) represent each IC in a multidimensional space (eleven IC-fingerprints) and classify ICs into six categories (one signal and five noise categories) using a support vector machine. Tohka et al. (2008) use a combination of spatial and temporal criteria to aid in classifying signal and four classes of noise via global decision trees. SOCK (Bhaganagarapu et al., 2013) automatically extracts four features from each IC and identifies artefacts according to five conditions. Sochat et al. (2014) adopted a sparse logistic regression with elastic net regularization method based on more than 200 features (spatial, temporal and power spectra information of individual IC and time courses) to automatically identify artifacts, showing high accuracy (Du et al., 2016). These techniques have been incorporated into the GIFT toolbox (http://mialab.mrn.org/software/gift/). FIX, developed by Salimi-Khorshidi and colleagues (Griffanti et al., 2014, Salimi-Khorshidi et al., 2014) extracts over 180 features from each IC and classifies the ICs into signal or noise using a hierarchical fusion of classifiers (namely k-Nearest Neighbour, support vector machines and decision trees) (http://fsl.fmrib.ox.ac.uk/fsl/fslwiki/FIX).

Although many of these approaches are fully automated, the gold standard for component classification remains the visual inspection of the components (Kelly et al., 2010, McKeown et al., 1998, Moritz et al., 2003). Manual classification is typically used to test the efficacy of newly developed approaches (Bhaganagarapu et al., 2013, Perlbarg et al., 2007, Rummel et al., 2013, Storti et al., 2013) and/or to create training datasets for supervised algorithms (De Martino et al., 2007, Salimi-Khorshidi et al., 2014, Tohka et al., 2008). Moreover, small sample size or unusual characteristics of a given dataset might require full manual labelling for effective artefact removal. Finally, being able to double-check the output of the automatic classification when applying any of these methods on a new dataset is highly recommended.

The operation of manual labelling, besides being time consuming, requires expertise. A general consensus on IC classification would provide more objective test-beds for training, testing and checking ICA-based cleaning procedures. This raises the need for guidelines on component classification.

The spatial and temporal patterns of the signal components are well documented in literature when describing resting state networks (RSNs). They are described both at group level (Beckmann et al., 2005, De Luca et al., 2006), and at the single subject level (Beckmann, 2012, De Martino et al., 2007, Kelly et al., 2010, Rummel et al., 2013, Salimi-Khorshidi et al., 2014, Smith et al., 2013). However, if a component does not match commonly presented RSNs, this does not necessarily mean that it should be classified as noise. This is especially true when, depending on the ICA dimensionality used, components might only contain a portion of the commonly defined RSNs (i.e., may be “sub-networks”), or different RSNs can merge into a single component. The aim of ICA-based artefact removal is to retain as much signal as possible, while removing structured noise to cleanup the data. The reason for this is that, for many applications of rfMRI, one probably cares more about keeping good signal than removing bad, particularly if the effects of residual artefacts can be ameliorated elsewhere, e.g., through the use of partial correlations in network modelling, where a move from full to partial correlation will address issues of ‘shared’ confounds. Therefore, a rule of thumb is that a component should be kept in the data, unless it is clearly artefactual (“innocent until proven guilty”). This highlights the value in identifying general rules and features for signal and noise components, more broadly than enforcing that single-subject RSNs have a strong spatial match to known (typically group-level-derived) RSNs.

The two main noise categories are noise related to the subject (motion/physiological effects) or related to the acquisition (MR physics artefacts). Therefore an understanding of both basic physiology and MR physics is important for correct interpretation. A detailed description of the possible range of artefactual components is more difficult to find in the literature and very few papers report descriptions or examples of artefact-related components (Beckmann, 2012, Kelly et al., 2010, Rummel et al., 2013, Salimi-Khorshidi et al., 2014, Smith et al., 2013). Rummel et al. (2013) described a set of rules that their raters adopted and Kelly et al. (2010) proposed a procedure based on some quantitative measures and hierarchy rules. However, the examples in these papers are usually related to a specific dataset of interest. In Salimi-Khorshidi et al. (2014) examples from three datasets are shown, although all acquired on a 3T scanner. Moreover, most of these papers do not describe in detail the specific strategy adopted to visually detect the N-ICs.

The aim of this “how-to” paper is to provide actual guidelines, practical pipelines and enrich them with examples, to help identify noise components in single-subject ICA for manual cleaning, training supervised algorithms or checking results of (un)supervised algorithms. First, we provide general guidelines about which features to evaluate when classifying a component, and strategies for optimal visualisation of the components. To this aim we merged knowledge and guidelines already present in the literature with practical suggestions derived from the direct experience of the authors. Second, we give practical examples from a variety of datasets (3T data, including examples from the UK Biobank Imaging study and the Human Connectome Project - HCP, and 7T data), in order to provide a wide spectrum of examples. Finally, we discuss some of the factors influencing the ICs and present examples of particularly challenging applications.

## 2 How to evaluate components

### 2.1 What to look at when evaluating ICs: “Features”

Classifying a component into signal (S-IC) or noise (N-IC) benefits from using three complementary pieces of information: the IC spatial map, its time series and its power spectral density (magnitude of the Fourier transform of the time series). Most of the guidelines and automated approaches evaluate all of these, but they differ in the number and type of characteristics (features) of spatial maps, time series and power spectra to consider. In this section we provide a general overview of the main features that can be visually evaluated when classifying ICs (Table 1).

**Table 1:** Features of signal- and noise-related independent components.

**Features**	**S-IC characteristic**	**N-IC characteristic**
Spatial		
Number and dimension of clusters	Low number of large clusters	Large number of small clusters
Overlap with GM	Clusters’ peaks in GM and overall good overlap of the clusters with GM.	Indiscriminate overlap with non-GM tissues, or clusters’ peaks in WM/CSF
Overlap with WM, CSF, blood vessels	Very low or absent overlap with WM, CSF, blood vessels	High overlap with one or more of WM, CSF, blood vessels
Overlap with brain boundaries or areas close to the edges of the FOV.	Very low or absent overlap with brain boundaries. Clusters follow known anatomical (e.g. structural/ histological) boundaries.	Ring-like or crescent shape or stripes near the edges of the field-of-view
Location near area of susceptibility induced signal loss (e.g. orbitofrontal)	Generally located away from these areas	Located within the region of signal loss (e.g. areas of air-tissue interface)
Non-biological, acquisition-related patterns	Patterns have no relation to acquisition parameters	Often show banding patterns in slice direction or streaks along the phase encoding direction, accelerated sequences may have centrally located artefacts
Temporal (and spectral) features		
Overall aspect of the time series	Fairly regular/oscillatory time course	Large jumps and/or sudden change of oscillation pattern.
Distribution of power in frequency domain	Predominantly low frequency (at least one strong peak within 0.01 – 0.1 Hz)	Predominantly high frequency, very low frequency, or pan frequency

Legend: GM = grey matter; WM = white matter; CSF = cerebrospinal fluid.

Spatial maps of signal components should contain a low number of relatively large clusters, while the presence of small and scattered clusters suggests the presence of a noise component. It is important to remember that spatial smoothing influences this feature: if no or little smoothing is being applied during data preprocessing, the spatial maps will contain more small scattered clusters, without necessarily implying that they contain more noise (see following sections for details). The localisation of clusters (and their peaks) in the grey matter (GM) suggests the neural-related origin of the component, while clusters mainly located in the white matter (WM), cerebrospinal fluid (CSF) and blood vessels (particularly arteries) are usually related to physiological noise (respiration, pulsation). The presence of clusters near brain edges suggests the presence of motion-related artefact (especially if the cluster has a ring-like or crescent shape or stripes near the edges of the field-of-view - FOV) or susceptibility artefacts in areas with air-tissue interface (mostly orbitofrontal gyrus and temporal poles in the case of typical echo-planar imaging). The presence of non-physiological patterns, such as positive/negative stripes or clusters visible only in a single slice or alternating slices, or streaks going in the phase encoding direction, are usually related to the MRI sequence (e.g., EPI susceptibility or multiband acceleration) or hardware artefacts (e.g. RF interference), or interactions of the acquisition with head motion (e.g., interleaved slice acquisitions).

Regarding temporal features, the main characteristic to look for in the time series is the presence of sudden jumps in the signal, likely suggesting rapid motion. Also, the oscillation pattern should not change significantly across the time course.

The BOLD signal is characterized by low frequency fluctuations, with highest power between 0.01 – 0.1 Hz. Although valid signal is present at higher frequencies (Chen and Glover, 2015, Kundu et al., 2012, Niazy et al., 2011), one of the main indicators of a BOLD-related signal component is the presence of predominantly low-frequency power, visible in the power spectrum as a low-frequency peak, but also in the time series (at least for data with shorter TR) as regular low frequency oscillations. However, due to the long TR of standard BOLD EPI (~2 to 3 s), the physiological noise due to cardiac and respiratory cycle (~1 Hz and ~0.3 Hz, respectively) often becomes aliased into this low-frequency range (Lowe et al., 1998, Murphy et al., 2013). Thus, cardiac and respiratory noise (see Section 2.3. *Examples of commonly seen noise components*) will also appear as low-frequency fluctuations and may be mistaken for neural activity-related BOLD signals. For this reason the performance of ICA-based classification is generally enhanced by using faster TRs (Griffanti et al., 2014).

### 2.2 How to look at the components

The aim of this section is to provide recommendations on how to look at the components to better evaluate the features described above. We will also provide some practical examples in the next section.

Regarding tools for visualising components, there is no single recommendation. Desirable requirements are the ability to visualise the spatial maps in the 3 planes at different thresholds along with the corresponding time series and power spectra at the same time. For example, Melview (http://fsl.fmrib.ox.ac.uk/fsl/fslwiki/Melview) provides such functionality, which is now embedded into FSLeyes (Fig. S1, panel a, a replacement for FSLview that will be part of the next upcoming FSL version and used for the examples presented here); Connectome Workbench is another effective viewer, which allows display of cortical surface maps of ICA components and for which the HCP provides standard view setups for each dataset, to visualize the ICA components (http://www.humanconnectome.org/software/connectome-workbench.html, Fig. S1, panel b). Other available packages for visualization are GIFT (http://mialab.mrn.org/software/gift), which provides a complete view of spatial and frequency content as well as an automated classification machine learning tool (http://journals.plos.org/plosone/article?id=10.1371/journal.pone.0095493), and AFNI (https://afni.nimh.nih.gov/afni/) which in addition to spatial and temporal visualization, includes probabilities for GM and WM.

*Change threshold:* when looking at the spatial maps, it is often useful to start with a default thresholding of the z-maps (usually around 2–3) and look at the number and dimension of the clusters (although some coauthors prefer to start by viewing unthresholded maps, as these maps show the full patterns of the data). Setting a higher threshold can help identifying the peaks of spatial maps, to help confirm that they are in the GM. When lowering the threshold, smaller clusters, if part of RSNs, would become bigger and follow the GM anatomy; sometimes a contralateral cluster or another cluster usually part of that RSN will also appear. On the contrary, clusters that are part of a noise component probably remain small, localized blobs, or expand with a spatial pattern not overlapping the GM.

*Change plane:* look in different planes to make sure the cluster(s) follow the GM ribbon in all views (a cluster might look like signal in one plane but not in others). Physiological N-ICs for example, will follow arteries and veins (e.g. the anterior, middle and posterior cerebral arteries are more visible in the axial plane, while the sagittal sinus is best viewed in the sagittal plane, see examples in the next section). Changing planes can also help detect MRI-related artefacts, for example the presence of signal in alternating slices.

*Underlay different modalities*. Components are usually displayed overlayed onto a mean EPI image. This is useful for evaluating susceptibility dropouts and other artefacts specific to the EPI acquisition. However, whenever available, it is also helpful to overlay the ICs onto a high-resolution structural image (e.g. T1w or T2w), to see anatomical details, especially cerebellum and basal ganglia, but also the GM folding, and, in some cases, major arteries/veins, in order to better evaluate the spatial overlap with the GM. The structural images need to be accurately aligned to the fMRI data (including correction for EPI distortions) for this to be useful.

*Look at both positive and negative clusters.* ICA spatial maps are often oriented so as to show strong positive clusters, but sometimes the main clusters are shown as negative areas, and/or ICs contain both strong negatives and positives values. Observing primarily negative signal ICs does not imply that these should be considered noise, as sign flipping does not affect the analyses or the spatial independence of the components. If the negative pattern appears as a signal component based on the other features, for artefact removal purposes, it should be kept in the data.

*Compare time series with realignment parameters*. If the spikes seen in the time series correspond to a sudden rotation/translation as seen in the realignment parameters generated in the preprocessing phase, the component will be likely to contain motion-related noise.

*Smooth data or components.* Data with high spatial and temporal resolution and sufficient number of timepoints will likely not benefit from spatial smoothing in the preprocessing (for example data from the Human Connectome Project are not spatially smoothed). This holds particularly for longer scans (and concatenation across multiple shorter runs from a given session might enhance the performance of ICA). On the other hand, unsmoothed components can have a noisier pattern than smoothed ones and the latter may therefore be easier to classify, as they more resemble group-ICA RSNs, well described in literature (Beckmann et al., 2005, De Luca et al., 2006). Therefore, for datasets with smaller overall signal strength (e.g., short time series), it may be necessary to spatially smooth the data before running ICA, in order for the ICA to function well. Alternatively, if pre-smoothing has not been applied, visualising (potentially noisy) ICs may be aided by having an additional version of the ICs that have been spatially smoothed (after generation by the ICA processing) for display/identification purposes only.

*Look at components in surface space*: After running ICA on the volume time series, components can be visualized in surface space. This can be done by performing a temporal multiple regression on the data in CIFTI grayordinates (Smith et al., 2013), using the ICA component time courses as temporal regressors, thereby finding the corresponding surface space component spatial maps (De Martino et al., 2007, Smith et al., 2013). In this space, signal components will map more consistently onto the surface than noise components do, even if they look more similar in the volume.

In order to most effectively identify the full range of artefacts, it is likely optimal to run the ICA in volumetric space, as carried out in the HCP (Smith et al., 2013), because volumetric space includes both gray matter and non gray matter voxels, which helps the decomposition algorithm to better separate components. The HCP's manual classifications used visualisation on the surface when training FIX, resulting in particularly high performance rates (Smith et al., 2013) in trained FIX performance. After training FIX, new datasets were then denoised by running ICA and FIX in volume space, and then regressing noise IC time series out of both volumetric and CIFTI grayordinate (Glasser et al., 2013) versions of the time series data.

*Expect a high proportion of noise components*. If the algorithm used for ICA decomposition orders the components according to the amount of explained variance (as done by FSL's MELODIC), many of the initial components will typically not contain signal. Usually, there will be many more noise components than signal (>70%), as shown in previous works:

– standard sequences at 3T: around 70% in (Rummel et al., 2013) and 88% in (Griffanti et al., 2014);
– multiband sequences at 3T: 90% in the HCP (Smith et al., 2013) and 88% in (Griffanti et al., 2014);
– standard sequence at 1.5T, 82% in (Griffanti et al., 2015).

Such large fractions of artefact seen in the literature provide a big incentive for carrying out data cleanup such as that described in this paper.

*Set priorities*. Manual IC classification is, many times, not straightforward, and needs the application of if-then rules. In the context of removing noise from fMRI data, when manually labelling ICs, the dominant aim is typically to preserve as much neural signal of interest as possible (Kelly et al., 2010). Therefore, following the “innocent until proven guilty” criterion, a component should be kept in the data, unless it is clearly artefactual. Kelly and colleagues (Kelly et al., 2010) based their classification procedure mainly on the inspection of the spatial maps. If in doubt, and the IC is likely to contain at least 90% of noise, other secondary criteria involving the evaluation of time series and power spectrum can be used to help with the final decision. Although we agree that the spatial map is the major piece of information to discriminate S-ICs from N-ICs, we strongly recommend always checking also the time series and the power spectrum, not only if doubts arise from the spatial maps, but as part of a hierarchical decision process.

The decision process we suggest to classify a component as signal is:

1) spatial maps first and foremost need to be plausible, i.e. be located in GM, away from the main veins, WM or CSF (with no obvious noise-related features as described above);
2) time series should be without sudden, abrupt changes;
3) power spectra should largely show power at low frequency (or at least the low frequency content should be larger than the high frequency one).

### 2.3 Examples of commonly seen noise components

After describing the components’ features and several strategies for their evaluation, we present some example categories of noise components, highlighting their features and giving guidelines to better recognise them.

For the purpose of fMRI data cleaning and/or training supervised automated algorithms there is not necessarily a need to subdivide the noise components into sub-categories, but only to distinguish between S-IC (not to be removed) and N-IC (to be removed). However, it can be useful to know the characteristics of specific artefacts, to be able to recognise them when they are clearly identifiable, but also to generalise the rules to apply when the classification is not straightforward.

The example components that are shown in this section are from the UK Biobank imaging study acquired at 3T (http://www.ukbiobank.ac.uk/scientists-3/ and http://biobank.ctsu.ox.ac.uk/crystal/refer.cgi?id=1977). Acquisition parameters are as follows: TR/TE = 735/39 ms, resolution 2.4 × 2.4 × 2.4 mm, 490 volumes, multiband acceleration factor 8; preprocessing: motion correction, B0 unwarping, brain extraction, no smoothing, no intensity normalization and high-pass temporal filtering with cut-off 100 s). The quality of this data can be considered “leading-edge” (attaining relatively high spatial and temporal resolution by leveraging the HCP's multiband developments), but still attainable (the acquisition time was 6 min and only standard hardware was used – Siemens Skyra with 32-channel head coil).

More examples of the same kinds of artefacts from a range of datasets are provided in the Supplementary material:

– Supplementary Figs. S2–S12 are from a (historically more typical) 3T dataset (Bijsterbosch et al., 2014), using a standard pre-multiband protocol: TR/TE = 2210/30 ms, resolution 3.5 × 3.5 × 3 mm^3^, 150 volumes (5 min 30 s), no multiband acceleration; preprocessing: motion correction, B0 unwarping, slice timing correction, spatial smoothing with a Gaussian kernel of 5 mm FWHM, and high-pass temporal filtering with cut-off 100 s.
– Supplementary Figs. S13–S24 are from the (leading-edge protocol high quality data, with custom hardware and long scanning sessions) Human Connectome Project (HCP), 3T scans (http://www.humanconnectome.org): TR/TE = 720/33.1 ms, resolution 2 × 2 × 2 mm, 1200 volumes per run (~15 min), multiband acceleration factor 8; see (Smith et al., 2013) for details about acquisition and preprocessing; no smoothing applied).
– Supplementary Figs. S25–S35 are from a 7T dataset (Siemens Magnetom, TR/TE = 1853/25 ms, resolution 1.2 × 1.2 × 1.2 mm, 220 volumes (~7 min), multiband acceleration factor 4; preprocessing: motion correction, B0 unwarping, brain extraction, intensity normalization and high-pass temporal filtering with cut-off 100 s; no spatial smoothing applied).

**Fig. 1:**
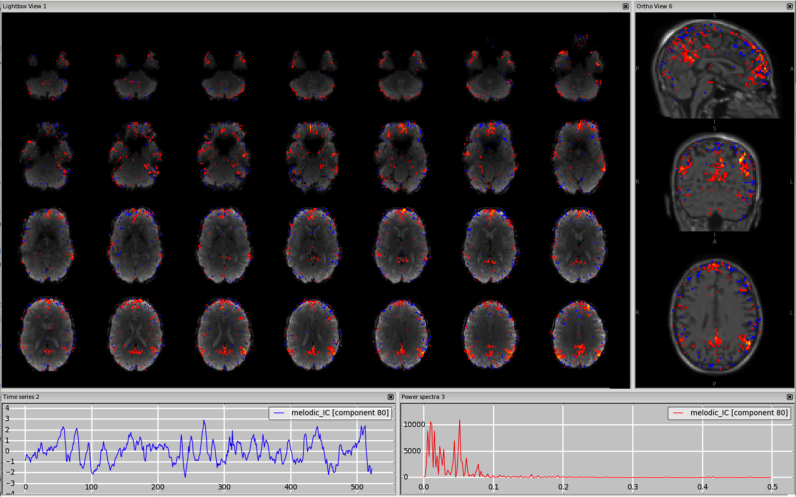
Signal. An example signal component showing the Default Mode Network (DMN). The time series (left plot) does not contain sudden jumps and the power spectrum (right plot) is predominantly low frequency. The change of viewing plane and the use of a structural image (e.g. high-resolution T1w) as underlay can help evaluating whether the clusters are localised in the GM (right panel). Threshold z = 2.3.

*Signal*. As reported in Table 1, a clean signal component would have a spatial pattern localised in the GM, low frequency power spectrum and no sudden jumps in the time series. Fig. 1, Appendix A (3T standard), S13 (HCP), S25 (7T).

**Fig. 2:**
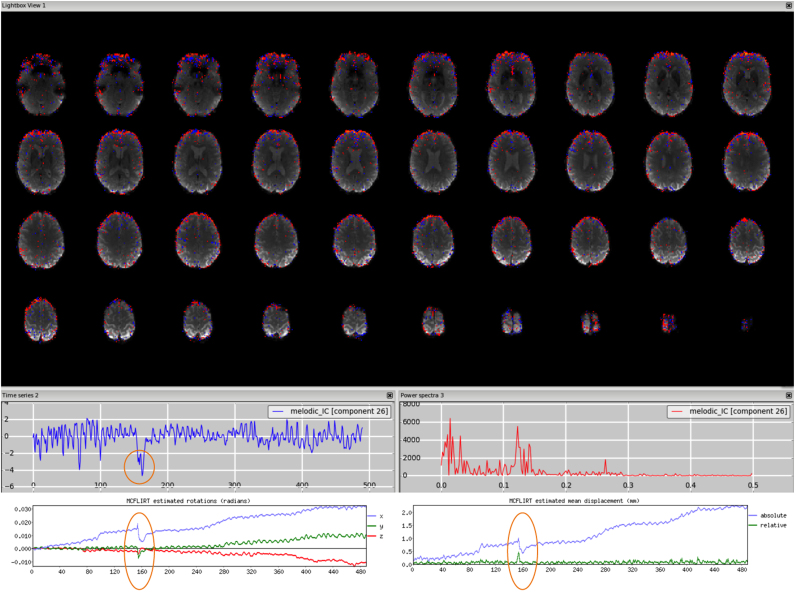
Motion artefact. The spatial map presents the typical ring at the edge of the brain and the time series contains a sudden jump in correspondence to sudden head movement, as visible in the motion-correction realignment parameters (highlighted in the orange circles).

*Motion*. This type of artefact is mostly seen well as a ring around the edge of the brain or as stripes close to the edge of the FOV, if it is quite tight. The voxels in these areas are inside or outside the brain/FOV depending of the subject's head motion, therefore the time series might follow the trend of the realignment parameters and sudden jumps or gradual drifts should be visible in both in the time series and the head motion profile. Fig. 2, Appendix A (3T standard), S14 (HCP), S26 (7T).

**Fig. 3:**
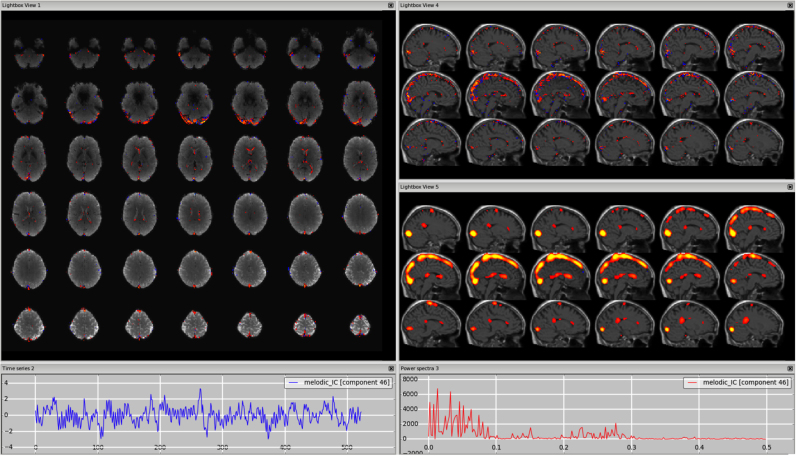
Vein (e.g., sagittal sinus). The vessel is most visible in the sagittal plane, with a structural image as underlay (top-right panel). A similar pattern is visible on smoothed ICA components (in this dataset a smoothing of FWHM = 9.4 mm was applied to the IC maps for visualisation purposes only) (bottom-right panel).

*Veins (e.g. sagittal sinus).* The signal coming from the veins is usually low frequency, so the time series and the power spectrum can sometimes be very similar to those from a signal component. These artefactual components are most often detectable from the spatial maps and this requires some knowledge of the anatomy of the veins. In particular, the main vein that is most commonly detectable as a physiological noise in ICs is the sagittal sinus. Kelly and colleagues presented an example in their paper (Example 5 in (Kelly et al., 2010)) showing the component in the axial plane with a fixed threshold. As shown in Fig. 3, Appendix A (3T standard), S15 (HCP), S27 (7T), the vessel can become more visible in the sagittal plane, and changing the threshold helps confirm that the peak is actually outside brain tissue; the use of a structural image as underlay can also help. Other veins often visible in the ICs are the straight sinus and the transverse sinus. It is important to remember that for a single dataset there could be multiple ICs containing signal from the veins (e.g., the sagittal sinus might be broken down in several ICs). Veins also make characteristic “staining” patterns on the cortical surface, tending to produce stripes across the tops of gyri.

**Fig. 4:**
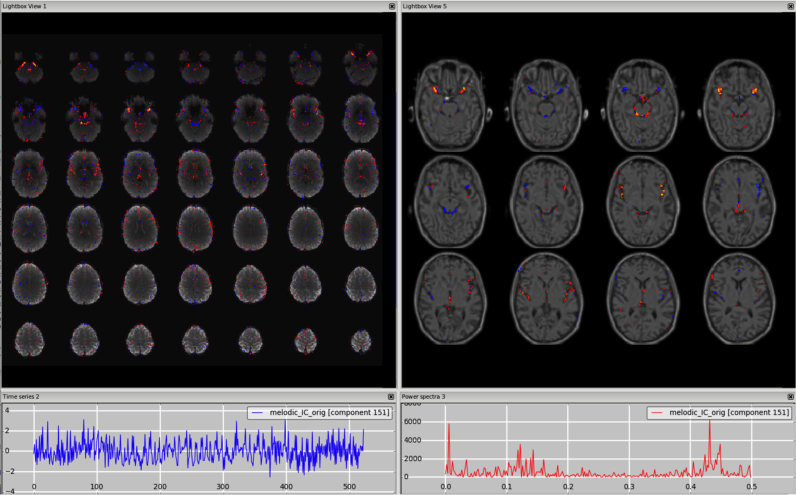
Arteries. The middle cerebral branches run close to the insula, so a structural image as underlay can help localise the vessels. Changing the threshold (in this case from z = 2.3 to z = 3) helps confirm that the peaks are not in the GM (right panel), although this would probably have been hard to see if the data had been pre-smoothed. This type of component also has a distinctive power spectrum (right plot).

*Arteries (anterior, middle and posterior cerebral arteries).* While in this case some knowledge about the anatomy of the brain vessels is again needed, the components containing BOLD signal coming from the arteries also have a distinctive high frequency spectrum. How distinct this peak will appear from the rest of the spectrum depends on the TR. As mentioned in Section 2.1 (*What to look at when evaluating ICs: “Features”*), the cardiac pulsation, usually around 1 Hz, will be aliased at lower frequencies for TRs around 2–3 s (Lowe et al., 1998, Murphy et al., 2013), making this component less easy to distinguish (see Fig. S5). Also in this case the change of the threshold, the use of additional anatomical information from the structural scan and the change of plane along the direction of the vessel of interest are useful ways to detect such a component. A particularly interesting case is the middle cerebral branches (ramifications of the middle cerebral arteries), which run through the insula. This is a very good example of a case in which the judgment of just the spatial map might be misleading, and careful inspection is needed to distinguish GM signal in the insula from physiological noise with the help of the temporal and spectral characteristics. See Fig. 4, Appendix A (3T standard), S16 (HCP), S28 (7T). However, it is not always possible to disentangle the neural related signal from the physiological noise. If this is the case, the component should be kept (following the “innocent until proven guilty” criterion).

**Fig. 5:**
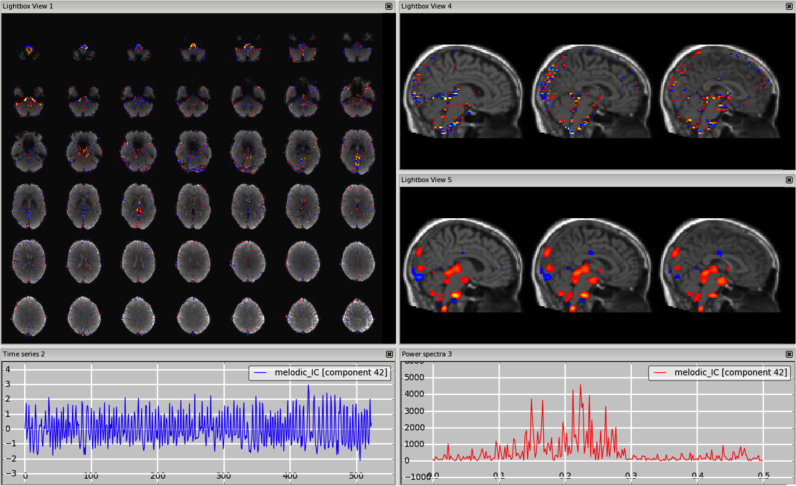
Cerebrospinal fluid pulsation. The spatial pattern overlaps the third and fourth ventricle, the cisterna magna and the aqueduct of Sylvius (threshold z = 2.3). This is seen most clearly when overlaid onto a structural image looking at a different plane at a higher threshold (top-right panel, z = 3) and after smoothing of the IC map (bottom-right panel).

*Cerebrospinal Fluid pulsation.* The CSF pulsation is mainly due to cardiac and respiratory cycles. Frequencies of the cardiac and respiratory cycles are around 1 Hz and 0.3 Hz respectively; therefore, also in this case, the corresponding signal is aliased into lower frequency for standard TRs (see also Section 2.1*What to look at when evaluating ICs: “Features”* and (Lowe et al., 1998; Murphy et al., 2013)). The spatial pattern of ICs containing signal from the CSF will be overlapping the ventricles and the cortical CSF, although sometimes it can be hard to distinguish the contribution from arteries. Fig. 5, Appendix A (3T standard), S17 (HCP), S29 (7T).

**Fig. 6:**
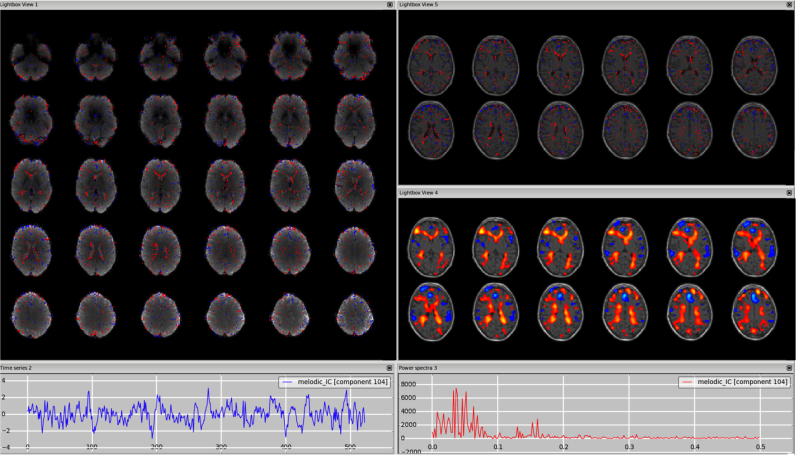
Fluctuations in subependymal (and transmedullary) veins. The spatial pattern overlaps the WM-CSF boundary, better localised overlaid onto a structural image (top-right panel). After smoothing of the IC map, the clusters extend more into the WM (bottom-right panel).

*Fluctuations in subependymal (and transmedullary) veins.* Although often defined as “white matter” components, they are due to primarily subependymal veins (Lang et al., 1981, Ohkawa et al., 1997). They have primitive connections with so-called transmedullary veins, which explain the clusters seen in the nearby WM, especially on smoothed data (Fig. 6, Appendix A). These components are best detected if overlaid on a high-resolution structural image, or any modality with good tissue contrast (e.g., fractional anisotropy maps from diffusion MRI). Fig. 6, Appendix A (3T standard), S18 (HCP). We did not find any clear component of this kind in the 7T dataset.

**Fig. 7:**
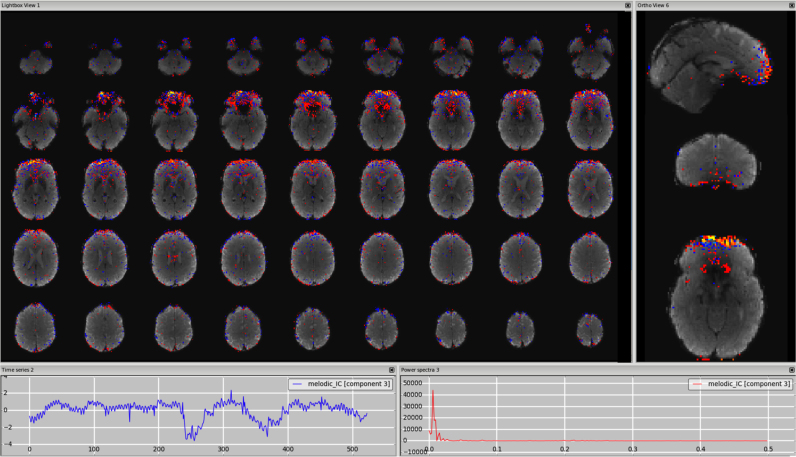
Susceptibility artefacts. Localised on the EPI in areas of signal drop, due mainly to air-tissue interfaces. The use of a higher threshold (in this case from z = 2.3 to z = 4) makes it possible to verify that the peak is in the region of signal drop (right panel).

*Susceptibility artefacts.* These kinds of acquisition-related artefacts are usually best detected from the spatial map as the associated time course can be predominantly low frequency, i.e., looking like a S-IC. Given that this artefact is related to the use of a gradient-echo pulse sequence, it is better to use the mean EPI image as underlay and look at the overlap of the IC clusters with areas of signal-drop in the EPI image, where the peak will be located when increasing the threshold. Unsmoothed, unthresholded images of susceptibility-related components will probably have tightly mixed high positive and negative values in them. Fig. 7, Appendix A (3T standard), S19 (HCP), S30 (7T).

**Fig. 8:**
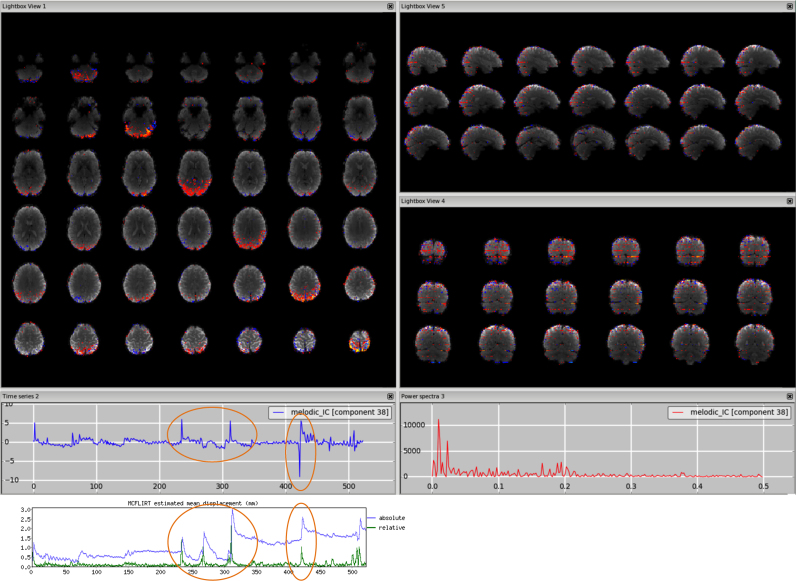
Multiband artefact. In the spatial maps, the clusters are visible in a regular way across slices (‘checkerboard’ effects), in this case every 8 (64 slices in z direction, multi band factor 8). This is reflected in stripes in the sagittal and coronal plane (right panels). The spikes in the time series suggest an interaction with head motion (as visible in the motion-correction realignment parameters, highlighted in the orange circles).

*Multi-band acceleration.* Another sequence-related artefact occurs when using multi-band acceleration (Feinberg and Yacoub, 2012, Moeller et al., 2010). The simultaneous acquisition of multiple slices is reflected in a typical spatial pattern within some ICs, where the clusters, usually with no clear neural-related pattern overlapping the GM, are present in sparsely and evenly spaced slices. These effects are often most clearly visible on unthresholded spatial maps. The slice spacing depends from the total number of slices and the multiband factor: for example, a volume with 64 slices acquired with MB acceleration factor 4 will give a MB-related artefact every 16 slices. These artefacts are therefore more visible in the sagittal or coronal plane as stripes, or in axial lightbox view as ‘checkerboard’ effects (all assuming axial acquisition). Such artefacts are not necessarily intrinsic to the acquisition/reconstruction, but may be an interaction of the multi-slice acquisition with head motion. Therefore, depending on the characteristic of head motion, the associated time course can be either low-frequency heavy or show strong peaks). Fig. 8, Appendix A (HCP), S31 (7T).

**Fig. 9:**
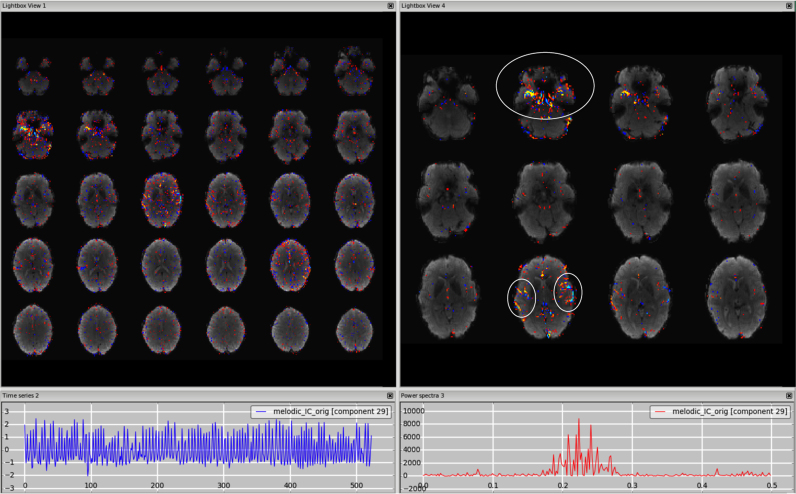
MRI-related artefact. The spatial pattern alternates between positive and negative values (highlighted in the circles on the right panel), and the high frequency spectrum (right plot) and the time series (left plot) are not physiologically meaningful.

*MRI-related artefacts.* Other artefacts can be related to MRI hardware or acquisition (Jezzard and Clare, 1999). Because of the complex nature of the imaging process, they can be quite hard to identify and understand fully. There are many different possible stages at which even slight deviation from expected performance of the individual components can result in large differences in the measurements. The most frequent case is the presence of alternating stripes of positive and negative z-values, as shown in Fig. 9. Other examples of MRI-related artefacts are shown in the supplementary figures: (i) pulse sequence timing instabilities generating not physiologically meaningful time course and spatial maps (Fig. S9, 3T standard), (ii) initial T1 saturation effect in the CSF at the start of the sequence (Fig. S21, HCP. This artefact is identifiable from the spatial map because it overlaps with ventricles and cortical CSF, as well as from the spike at the beginning of the time course), (iii) uneven fat suppression (Fig. S32. At 7T the signal caused by the fat from the eyes may in some cases appear as two hyperintense foci in the lower part of the brain).

**Fig. 10:**
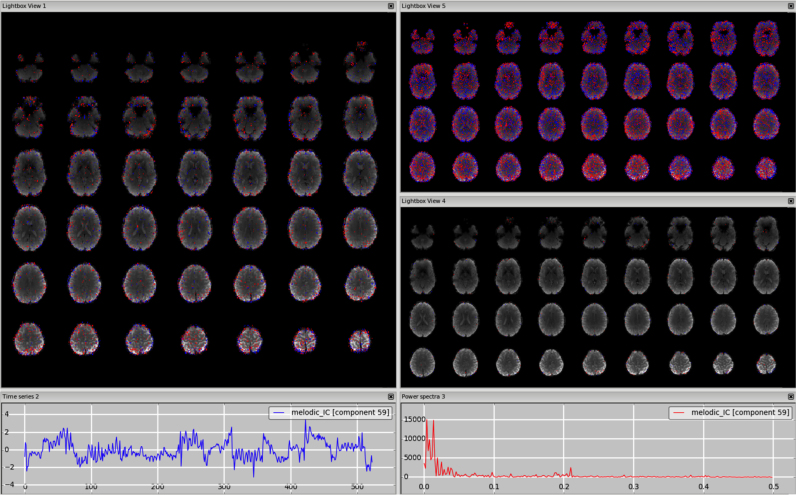
Unclassified noise. In this example, this component has a low frequency spectrum, with a not very smooth time series, a few temporal jumps/discontinuities, and a very scattered spatial pattern. Neither decreasing the threshold (in this case from z = 2.3 to z = 1, top-right panel) or increasing it (in this case from z = 2.3 to z = 4, bottom-right panel) shows any GM cluster that would likely have neural origin.

*Unclassified noise.* If the component we are evaluating does not fit into any of the previous categories but has one or more features typical of a noise component (see Table 1), we will simply refer at it as “unclassified noise”, count this as N-IC, and its contribution will be removed from the data. Fig. 10, Appendix A (3T standard), S22 (HCP), S33 (7T). Note that such cases are not the same as “unknown” (i.e., “mixed good-bad”) components.

Many more examples of S-ICs and N-ICs are freely accessible at www.fmrib.ox.ac.uk/analysis/FIX-training, where the hand-labels have been used to train FIX, the supervised ICA-based cleaning method (Griffanti et al., 2014, Salimi-Khorshidi et al., 2014) used in the main HCP, Whitehall II and UK Biobank processing pipelines.

### 2.4 Examples of often misclassified components

Among the example categories of noise components presented above, some of those are often misclassified as signal, due to their resemblance to known RSNs. In this section we discuss further their characteristics, in order to help in identifying them. In addition, we describe other areas where the components are often difficult to classify and deserve more careful inspection.

*Sagittal sinus or visual cortex?* Since in both cases the power spectrum is predominantly low frequency, it is important to consider the spatial maps and in particular the sagittal views. This will show that a sagittal sinus IC often has a pattern following the vessel up to the top of the brain (see Fig. 3), although not always (as in Fig. S15), while a visual-related IC will be localised in the occipital lobe GM. By changing the threshold, the artefact shows its maximum peak(s) outside the brain, while the RSN has peak(s) inside the GM, often bilateral.

*Arteries or insula?* In this case the power spectrum can help the judgement, as the BOLD signal coming from the arteries has a distinctive high frequency spectrum (see Fig. 4, right plot). The presence of clusters also in correspondence of other arteries (e.g. the posterior cerebral artery in Fig. S5), also helps classifying the IC as noise. As in the previous case, changing the threshold shows the peak(s) outside (noise) or inside (signal) the GM.

*Susceptibility or ventromedial prefrontal cortex?* The ventromedial prefrontal cortex is an area that is often affected by susceptibility artefacts, due to its proximity to the air cavities. In this case the distinction between signal and noise is not easily made by looking at the location of the cluster with respect to the GM, but of the cluster (and/or its peak) with respect to the area of signal dropout in the EPI image, set as underlay (see Fig. 7, S8, S19, S30). Another way to identify the orbitofrontal susceptibility components is to check if the time series follows the trend of the realignment parameters, since head nodding changes the dropout pattern in the EPI images. Finally, in multi-echo fMRI data, there will be an orbitofrontal component related to the different dropout in different echoes, which will be spatially signal-like, but with regular temporal pattern related to the sequence of TEs. Other areas that could be affected by susceptibility artefacts and therefore require careful inspection are the amygdala and the hippocampus.

*Deep GM structures*. The basal ganglia, the thalamus and hypothalamus are very close to the CSF, and therefore signal and noise from these structures can be difficult to disentangle. Regarding the spatial maps, it is important to increase the threshold to check the location of the peak. The signal in subcortical GM structures will have the peak(s) inside the structure, rather than in the ventricles or at the border between GM and CSF, and these peaks will usually be bilateral (unless present in a clearly lateralised S-IC).

*Cerebellum*. In many cases the cerebellar components have considerable spatial content in the veins. In other cases, there are components containing visual as well as cerebellar areas, which are close to the confluence of sinuses. Similarly to what can be done in case of ambiguous IC in the subcortical structures, increasing the threshold will help identifying the location of their peaks. For an S-IC, these peaks will be located in the GM and, specifically for "visual-cerebellar" components, either in the occipital or the top cerebellar area (rarely in both). In case of N-IC, the peaks will be in the veins. Often, particularly in shorter and lower spatial resolution datasets, one component spans cerebellar grey matter as well as spreading out into nearby veins; in general such components are a mixture of “good and bad” signal, and should not be removed from the data.

Also in these difficult cases, following the “innocent until proven guilty” criterion, if this further examination does not lead to a clear choice, the component should be kept in the data. More generic cases of no clear distinction between signal and noise are discussed in the following section.

### 2.5 What if still in doubt?

In cases when a clear classification into N-IC vs S-IC cannot be achieved, the components are usually labelled as “Unknown” and the general recommendation for these components in the cleaning phase is to keep them in the data, to avoid losing valid signal. However, it is important to note that unknown components can be generated from different scenarios.

**Fig. 11:**
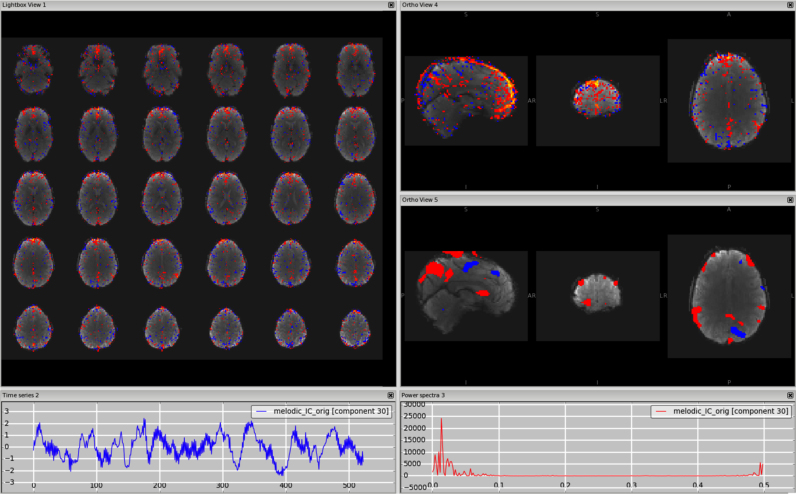
Unknown. In this example, the component contains clearly some neural-related signal (DMN), but also some artefacts, possibly of vascular origin, especially visible in the sagittal plane (right panels). The time series is mostly low frequency, but with a high frequency peak as well.

**Fig. 12:**
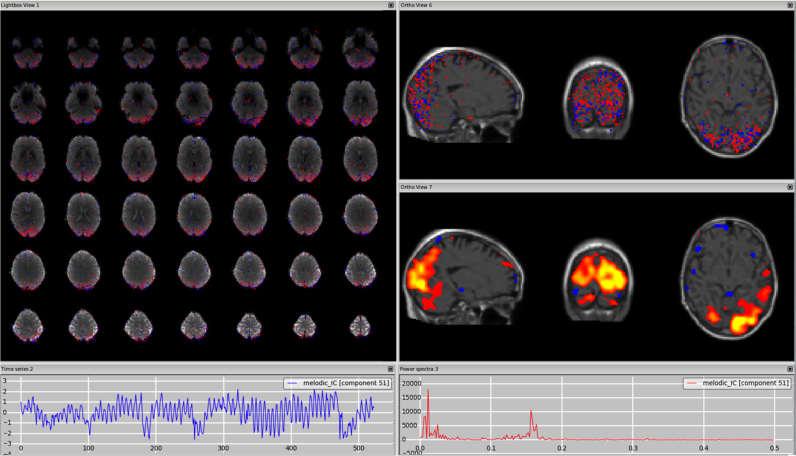
Unknown. Unlike the former example, there is no clear presence of signal and noise. The spatial pattern is mainly localised in the GM, but is not clearly attributable to an RSN, there is a positive/negative pattern but only on unsmoothed maps, and the power spectrum contains both low and high frequency peaks. Especially in these cases, careful inspection of different planes and smoothed data, also with the underlay of structural image (right panels), is good practice to help determine that the component does not belong to other categories.

First, ICs can contain a mix of signal and noise sources identifiable by their specific features. Sometimes the signal and noise can be clearly identified, as in Fig. 11 (DMN combined with sagittal sinus), other times the component may contain both features typical of signal and of noise, but they are not clearly attributable to a single RSN or to a specific artefact described above (e.g., spatial pattern with GM and non-GM clusters, low and high frequency peak, Fig. 12). In both cases, as there is some signal, these components should generally be kept in the data. This is especially true if the signal-like part of the component is in a key area for the study, and in a more difficult brain region for imaging (e.g. basal ganglia, brainstem, cerebellum). Fig. 11, Fig. 12, S11-S12 (3T standard), S23-24 (HCP), S34-S35 (7T).

This “unknown” category also helps ameliorate limitations in expertise of the manual labeller (Hartendorp et al., 2012, Murphy and Ross, 2010).

A way to reduce the uncertainty associated with the classification process is to involve multiple raters. A more formal and quantitative way is blind multi-session and/or multi-rater classification. This procedure gives a quantitative measure of the inter- and intra-rater variability and can be used to calibrate the labelling method (Kelly et al., 2010), and train new raters. However, the final classification is usually reached through a consensus of multiple experts, especially regarding unknown components (when the raters do not or cannot agree, the component is labelled as unknown) (Griffanti et al., 2014, Rummel et al., 2013, Salimi-Khorshidi et al., 2014, Storti et al., 2013). For example, the work by Rummel and colleagues (Rummel et al., 2013) involved three raters; after independent rating the raters agreed on N-ICs and S-ICs in a discussion session and then provided rating accuracies of individual raters compared to the raters’ agreement. Similarly, in Storti et al. (2013) two experts evaluated the components; the disagreements were discussed with the group, resolved by reference to a third author. A similar approach was adopted for the HCP data.

Finally, taking a second non-blind pass through classification by the same operator can further help inter-rater consistency as, by the end of the manual classification, the rater may have acquired expertise.

### 2.6 Any automatic help or alternative for components’ classification?

The guidelines for IC classification described so far do not require any additional calculations but only rely on visual inspection. This is time consuming and can appear quite subjective. Besides multi-session and multi-rater labelling or consensus strategies, the use of some more quantitative measures and/or automatic approaches can help the classification.

Kelly et al. (2010) for example, provided the rater with statistics about the proportion of thresholded voxels lying in the periphery of the brain, within non-peripheral CSF, WM, or GM, in order to facilitate determination of what percentage of supra-threshold voxels lie in functionally relevant GM clusters. These values can be helpful for a relative comparison among ICs within a dataset, but there is no specific value suitable for every dataset and application.

Another approach is to calculate the similarity of the spatial pattern with some template components both for RSNs and for noise. This can be a useful measure to help classification, but the ICA decomposition might split the components in a different way compared with more standard known RSNs, and so the comparison might not be useful.

In order to help the identification of movement-related N-IC, some quantitative measures can be calculated from the time courses. For example Vergara and colleagues (Vergara et al., 2016) used the DVARS method (Power et al., 2012) to find spike regressors where the root mean squared head position change exceeded 3 standard deviations. Other options are to calculate deviations of mean frame-wise displacement (Power et al., 2012) or the temporal correlation between the time course and the realignment parameters (Vergara et al., 2016).

The calculation of the ratio of total power above a given frequency to the power below that frequency from the Fourier transform can help better characterization of the frequency content. In Salimi-Khorshidi et al. (2014) several quantitative features were calculated using several different frequency thresholds (one per new feature): 0.1, 0.15, 0.2 and 0.25 Hz (and these features are used in the automated trained classifier FIX). Similarly, Allen et al. (2011) calculated the low frequency to high frequency power ratio as the ratio of the integral of spectral power below 0.10 Hz to the integral of power between 0.15 and 0.25 Hz (and this metric is included in GIFT). They also calculated a second metric, the dynamic range, i.e. the difference between the peak power and minimum power at frequencies to the right of the peak.

De Martino et al. (2007) proposed a representation of the components in a multidimensional space of quantitative measures (IC-fingerprints). The IC-fingerprint of a component can be visualised as a polar diagram and used as a tool to help the rater in the classification.

Another option is to use unsupervised automated methods or supervised automated methods with a default training dataset to get an initial classification of the components or to detect one particular type of artefact, and then iteratively refine the classification by hand by checking classifier/manual mismatches for errors. Indeed this approach was very helpful for training the HCP dataset, as it enabled catching a number of ‘transcription’ errors.

### 2.7 Classification of group-ICA

Most of the guidelines presented so far can be extended to also classify components extracted from group-ICA. Although it has been shown that the number of artefactual components decreases when effective noise removal is first performed at single subject level (Feis et al., 2015), the noise left in the data, either from mixed/unknown components or in the residuals of single-subject ICA decomposition, can appear at the group level if there is some spatial consistency across subjects. In order to select the RSNs of interest for later analysis steps, a group-level classification is often needed. Moreover, when performing network analysis, if full correlation is used to generate the network, the contribution of the group level noise components should be removed from the data in a “higher-level” cleanup phase before calculating the correlation matrix. If partial correlation is used, bad components should then be included when estimating the partial correlation (and then most likely ignored after the creation of the matrix), as their contribution is taken into account by partial correlation (Griffanti et al., 2014).

The advantage of classification of group-ICA over single subject ICA is that the spatial maps are less noisy and the RSNs are more clearly identifiable. The data are registered to a standard space and spatial priors of GM/WM/CSF are available to assess the overlap with these tissues. On the other hand, useful time series the power spectra are not immediately available from the primary group-ICA outputs. A way to evaluate the power spectra of group ICs is to derive for each subject the power spectrum for each component (e.g., performing the first step of dual-regression (Filippini et al., 2009)) and average these across subjects to obtain an average spectrum per component (Allen et al., 2011, Griffanti et al., 2014, Kullmann et al., 2013, Mattiaccio et al., 2016).

When running group ICA, it is also very important to ensure that images are well aligned across subjects. The HCP has used the CIFTI grayordinates standard coordinate space together with areal-feature-based registration (Glasser et al., 2013, Glasser et al., 2016) to achieve very good alignment of brain areas. Interestingly, relatively few artefactual group components were found in a d = 137 ICA decomposition of 210 subjects (1 vein, one arterial pulsation, and one coil artefact that was present in only 1 run out of 840). This was likely attributable to the excellent performance of the automated individual run noise removal from ICA+FIX, the noisy voxel time series rejection at the point of projecting volume data onto the surface (Glasser et al., 2013), and with the alignment quality, which eliminates components that might appear to be noise but instead arise from misalignment of RSNs across subjects.

## 3 Factors influencing ICs: Data quality, data type and preprocessing

ICs are influenced by multiple factors related to the subjects, the acquisition and the preprocessing. In this section we will discuss the impact of acquisition and preprocessing on the ICs, and in the next section we will present some challenging scenarios related to the population of interest.

Data from different scanners and sequences may require different preprocessing options and may benefit from some changes in the analysis pipeline for an optimal visualisation and classification of single subject ICs.

In general, high quality data (i.e., high spatial and temporal resolution data generated with higher field strength and/or accelerated sequences) usually allow for improved IC classification. Higher temporal resolution (short TR) allows ICA to better separate different frequencies, partly due to reduced aliasing, as the highest identifiable frequency is 1/(2 x TR) (Nyquist frequency) and contribution of higher frequencies will be artificially expressed in lower frequencies. The acquisition of many time points allows ICA to better separate components due to the higher number of degrees of freedom. However, a consequence of increasing the number of time points (higher temporal resolution or longer scans) is the generation of a higher (automatically estimated) number of ICs, the manual evaluation of which can be very time consuming and difficult, partly because commonly defined RSNs may be split into multiple sub-networks, and therefore more difficult to identify. One reason for the higher number of components is overfitting of noise, which can be reduced by constraining the maximum number of components (e.g., 250 is set as the maximum for HCP preprocessing). As mentioned in Section 2.2 (*How to look at the components)*, long runs of such data are generally best decomposed and manually or automatically classified without spatial smoothing.

Despite the trade-off between increased dimensionality (and possible refined cleanup) and difficulty (and possible inaccuracy) of manual classification, the general rules provided in this how-to paper can be applied at different dimensionalities. Together with the examples from different datasets (in addition to those already available online www.fmrib.ox.ac.uk/analysis/FIX-training), we aim to improve manual classification accuracy at any dimensionality.

Moreover, specific artefacts can be related to the sequence or scanner, and, although they can be visible already in the EPI images, are captured in specific ICs, which helps their identification. For example, the signal caused by the fat from the eyes may in some cases appear as two hyperintense foci in the cerebellum or temporal lobes in EPI images. This occurs as a result of imperfect/absent fat suppression, and can sometimes be seen in the ICs (see Supplemental Fig. S32). Other MRI-related artefacts that can be sometimes captured as ICs are ghosting, signal dropouts and RF interference (Jezzard and Clare, 1999).

ICA-based cleaning has also been used in multi-echo data (Kundu et al., 2012). In the Multi-Echo Independent Component Analysis (ME-ICA) method, developed by Kundu and colleagues, multi-echo data with at least 3 different echo times are decomposed with Independent Components Analysis (ICA) either after spatially concatenating data across space and TE (Kundu et al., 2012) or after creating a new single optimally combined time series per voxel, optimised for functional contrast (Olafsson et al., 2015). Based on the idea that S-ICs show a TE-dependent signal change, while N-ICs do not, the components are automatically classified according to two summary metrics (see (Kundu et al. (2012)) for further details). Kundu et al. (2012) show TE-dependence maps of ICA components as well as examples of signal components (functional networks) and artefacts. From those, it can be observed that the features of the spatial maps and time series described so far are similar to those of the ICs generated from single-echo data. Therefore, although ME-ICA is an unsupervised algorithm for detecting noise components, the guidelines provided here can be applied also in this context, to check the classification of ME-ICA or perform manual cleaning of multi-echo data.

ICA-based cleaning can also be used to clean task-fMRI data. It is less crucial to apply structured noise removal for task data, as the task-related signal of interest is hypothesised a-priori and so will not too often correlate by random chance with many types of structured noise. Moreover, the removal of many degrees of freedom (i.e. components) could reduce the statistical power of the first-level analyses, especially if the GLM design matrix is very complex. Nevertheless, the general guidelines for the identification of noise components described above are still applicable for task-fMRI data. Note that the components containing signal will include specific task-related components that may not look like RSNs.

Regarding the preprocessing phase, ICA-based cleaning is a powerful tool to clean many sources of noise from fMRI data, potentially with no need for additional cleaning. However, when the data is particularly corrupted by one or more types of artefact, the ICA algorithm might struggle to separate the components, creating a lot of components that are not clearly identifiable or are a mix of signal and noise. In such cases, and depending on the type of artefact, it could be useful to perform a multi-stage cleanup, including additional pre-processing or post-processing steps to the standard pipeline, or even run ICA cleanup twice (e.g., to help remove artefacts that are strongly nonlinear corruptions of the data). In general, operations that would improve ICA decomposition should be run before it (e.g., removal of volumes due to saturation effect at the beginning). On the other hand, some operations that would refine the cleaning (e.g., regression of physiological signal acquired during scanning), but which do not generally completely remove the artefact of interest, should be run afterwards. The rationale for this recommendation is that imperfect removal of a specific kind of artefact before running ICA could result in the artefact not being completely removed, but also possibly being too weak to appear in a distinct IC. If the artefact has been already successfully removed with ICA-based cleaning, the additional cleaning step should not corrupt the data in any case (as long as the same processing and cleaning steps applied to the data are also applied to the regressors used for additional cleaning). Despite these possibilities to improve ICA-based cleaning, this does not exclude the possibility that the data is too corrupted by one or more artefacts, and therefore unusable.

Another important consideration is that spatial ICA (whether for standard or multi-echo EPI) is mathematically unable to see global confounds. Fortunately, most noise sources (detailed above) are spatially specific, such as movement artefacts, scanner artefacts, or venus/arterial related artefacts. Artefacts related to changes in the perfusion pressure of the brain (e.g. variations in breathing rate/depth resulting in CO_2_ changes, heart rate variations, or even yawning) can result in global BOLD fluctuations (Golestani et al., 2015) that will be distributed across all of the signal components in the brain (though focused on the grey matter). Such global fluctuations pose a serious challenge to univariate methods of brain analysis such as standard functional connectivity by leading to global positive biases in connectivity and have led some authors to insist on the use of global signal regression to remove them (Power et al., 2015). Unfortunately, such methods make no distinction between global noise and global signal and will thus also distort measures of functional connectivity (Saad et al., 2012). Until we have a method for separating global signal from global noise, a better approach is to use multivariate analysis methods such as partial correlation (Smith et al., 2013).

## 4 Challenging scenarios

In this last section we briefly mention some challenging scenarios related to the manual labelling of data from “non-conventional” datasets, as the population of interest can influence the type and amount of artefacts. The artefacts presented so far are generally present both in healthy subjects and in patients. However, the proportion of these artefacts can change depending on the type of population, their variability (e.g., more or fewer CSF components due to more or less brain atrophy), and their compliance (e.g., more or fewer motion-related components). We highlight below some categories of subjects that are noteworthy for being particularly challenging in terms of IC classification.

*Altered neural brain activity*. A source of alterations of the BOLD signal and derived ICs is altered neural activity. For example, in the review by Soddu et al. (2011) the authors describe the difficulty in identifying resting state activity from ICA in patients with disorders of consciousness (locked-in, minimally conscious state, vegetative coma). In those patients, brain function is usually reduced (sometimes even limited to one hemisphere), and concomitant structural alteration can further complicate the detection of rfMRI activity. This results in RSNs sometimes being not detectable in an automatic fashion, but only by visual inspection. Similarly, Demertzi et al. (2014) found lower number of neuronal components and reduced functional connectivity in the identifiable RSNs in patients in minimally conscious state, vegetative state/unresponsive wakefulness syndrome and coma with respect to healthy controls.

RSNs are also altered during anaesthesia, being a condition of induced consciousness alteration. Guldenmund et al. (2016) observed that the detectability of RSNs from ICs diminished significantly with loss of responsiveness, and total brain connectivity decreased strongly in the frontal cortex in the detectable RSNs.

Epileptic patients represent another case of altered neural activity. Rodionov et al. (2007) showed that ICA on EEG-correlated fMRI data is capable of revealing areas of epileptic activity (interictal epileptiform discharges, IED) in patients with focal epilepsy as a separate class of ICs. For component classification they used an automatic IC classification approach to detect standard sources of noise, and then visually inspected the remaining ICs to identify the new class of IED-related components. In 7 out of 8 subjects the IED-related IC matched the GLM-based results of a previous case study, while some of the un-interpreted ICs (i.e. that did not satisfy both the spatial and temporal matching criteria) had a time course that was significantly correlated with the IED-derived GLM regressor, suggesting that those IC could describe abnormalities that are not apparent on scalp EEG.

*Acute vascular pathologies*. Other alterations of the BOLD signal may be caused by changes at the haemodynamic/vascular level. An example population that requires particular attention is patients who have had an acute vascular event (e.g., stroke). The vascular nature of the pathology can interfere with the BOLD signal, as acute ischemic lesions are able to alter the vascular tone and the cardiac and respiratory cycles (Soros and Hachinski, 2012). Moreover, when scanned in the acute phase, patients are likely to move more due to decreased compliance. The guidelines for IC classification described here are still applicable. However, due to the heterogeneity of the pathology and the localisation of the ischemic lesions, the number, type, and characteristics of the ICs are likely to be more variable across subjects. In a recent study, Carone and colleagues successfully applied ICA-based cleaning to a group of patients with ischemic lacunar stroke (Carone et al., submitted), but this required the creation of a study-specific set of manually-labelled components from two independent raters (inter-rater agreement 95%).

*Neonates and children*. Data from neonates and young children present significant challenges for data cleanup. Image contrast is different and more variable than healthy adults, as are structural and functional properties (Erberich et al., 2003). With standardised cleanup protocols yet to be defined for these diverse populations, careful assessment and tuning of data cleanup procedures is necessary to ensure that datasets are processed appropriately (Doria et al., 2010, Toulmin et al., 2015). Very high levels of head motion are typical, to an extent that a significant proportion of data may be unusable. High levels of motion will often lead to the ICA decomposition being dominated by motion components, which can be problematic for cleanup and analysis. Cleanup may need to involve a combination of data rejection (scrubbing), aggressive motion-parameter regression, and ICA-based denoising (Doria et al., 2010, Toulmin et al., 2015).

*Animals*. There is evidence for resting state activity in rodents and primates (see Gozzi and Schwarz (2016)) for a review) and in these cases ICA-based cleaning can also be used. Zerbi and colleagues for example (Zerbi et al., 2015) performed ICA-based denoising in data from mice. In their work they present many examples of signal (further divided into unilateral and bilateral) and noise components (motion, vascular, unspecified noise) from group ICA that can help single-subject classification. This demonstrates that, besides the anatomical differences in the brains of these animals and a different interaction of BOLD signal with cardiac and respiratory cycles, the guidelines presented here are adaptable to other species.

## 5 Conclusion

**Fig. 13:**
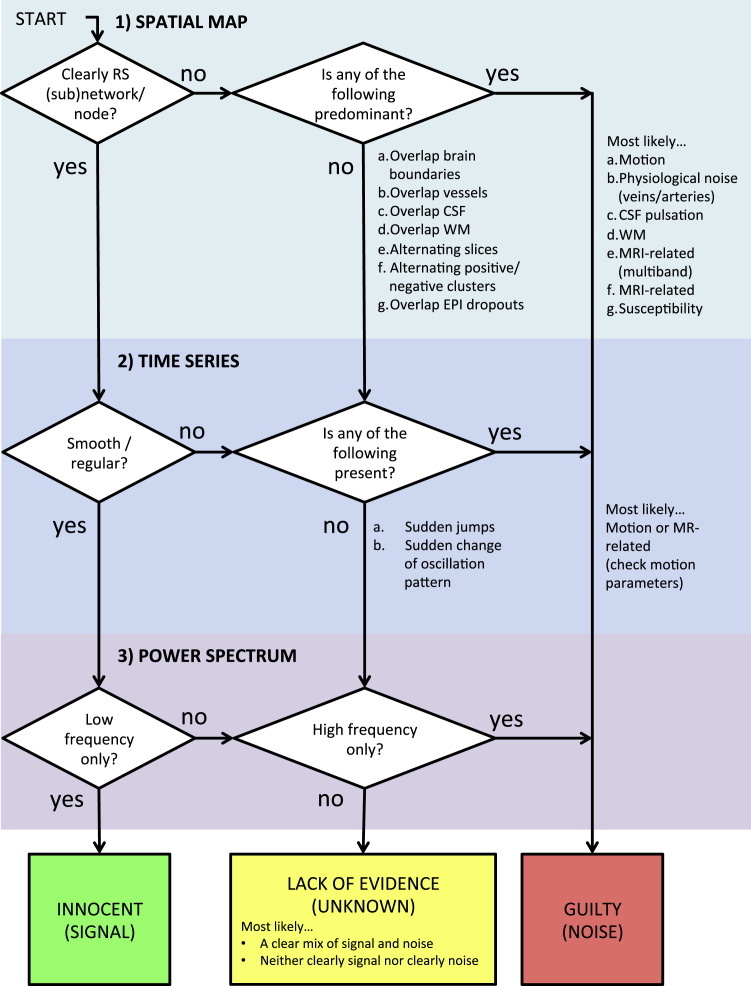
“Innocent until proven guilty” flowchart. A summary of the procedure for visual inspection and manual classification of ICs proposed in this paper. The key points are, first, the importance of evaluating all three pieces of information derived from ICA decomposition (spatial maps, time series and power spectra), although with some hierarchy. Second, the aim of retaining as much signal as possible, and not removing components not clearly identifiable as artefactual. The flowchart does not include natural loops involving double-checking decisions and specific cases, discussed more broadly in the main text.

In this “how-to” paper we provided guidelines and practical strategies for visual identification of noise components from ICA results. To this aim we merged knowledge and guidelines already present in the literature with practical strategies and visual examples derived from the direct experience of the authors. Fig. 13 summarises the main guidelines described here in what we called the “Innocent until proven guilty flowchart”, with the aim of retaining as much signal as possible while removing structured noise to improve fMRI analyses. This is of course a simplified version of a decision process that in real life would probably have many loops inside it, and that requires expertise and training beyond the application of simple if-then rules. Although this is a manual process with no absolute ground truth, and ICs can vary with different populations, acquisitions and processing, we believe that a consensus on general/typical characteristics of S-IC and N-IC is useful, to help provide a common reference for a more reliable and reproducible manual cleaning, training supervised algorithms or checking results of (un)supervised algorithms.

## Footnotes

Appendix A Supplementary data associated with this article can be found in the online version at http://dx.doi.org/10.1016/neuroimage.2016.12.036.
