# Supplementary figures and images for: Hand classification of fMRI ICA noise components
Source: Neuroimage. 2017 Jul 1;154:188–205. doi: 10.1016/j.neuroimage.2016.12.036 (PMC5489418; doi:10.1016/j.neuroimage.2016.12.036)

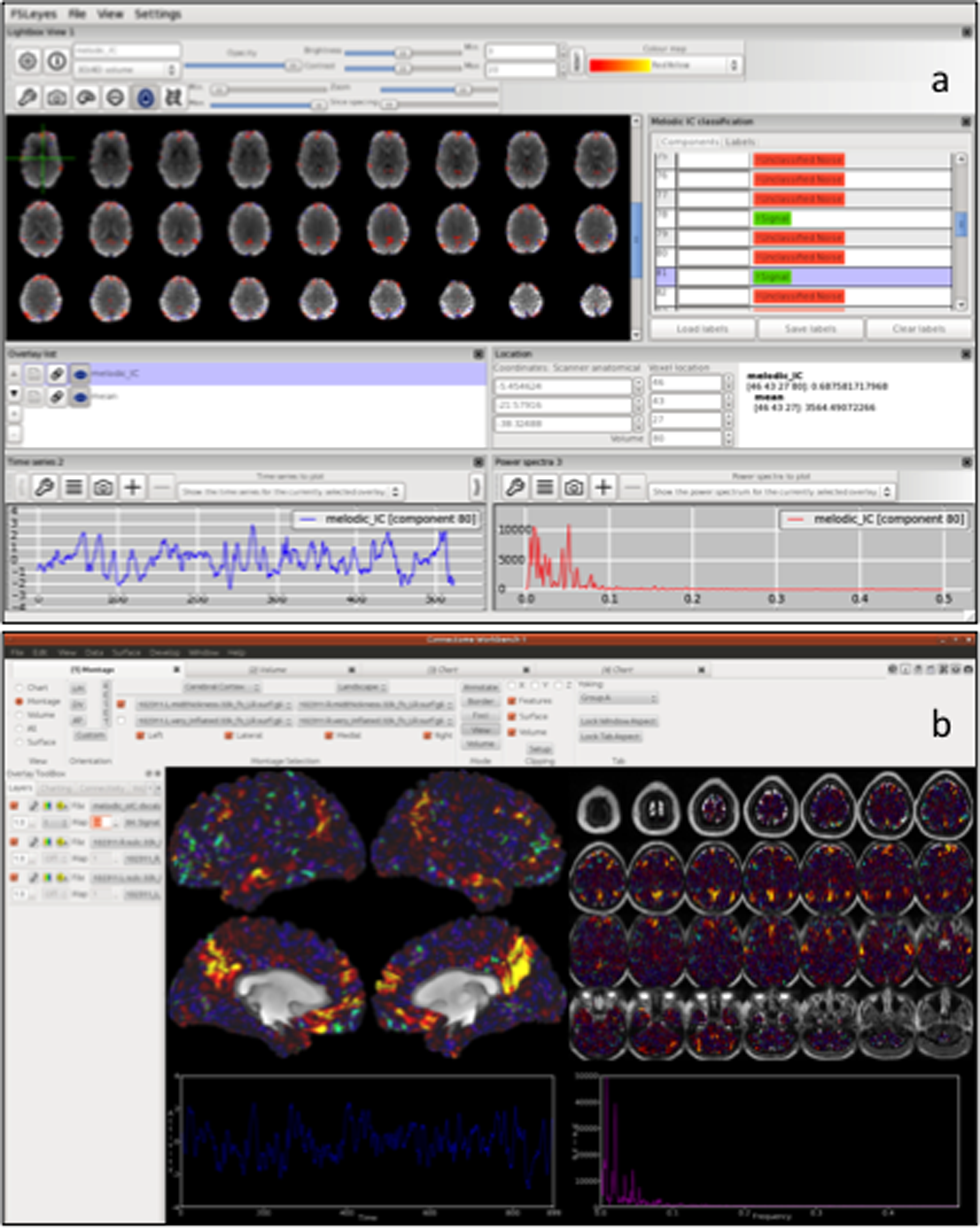

Supplement: Supplementary file 1 — Supplementary material [file mmc1.zip › mmc1.tif]

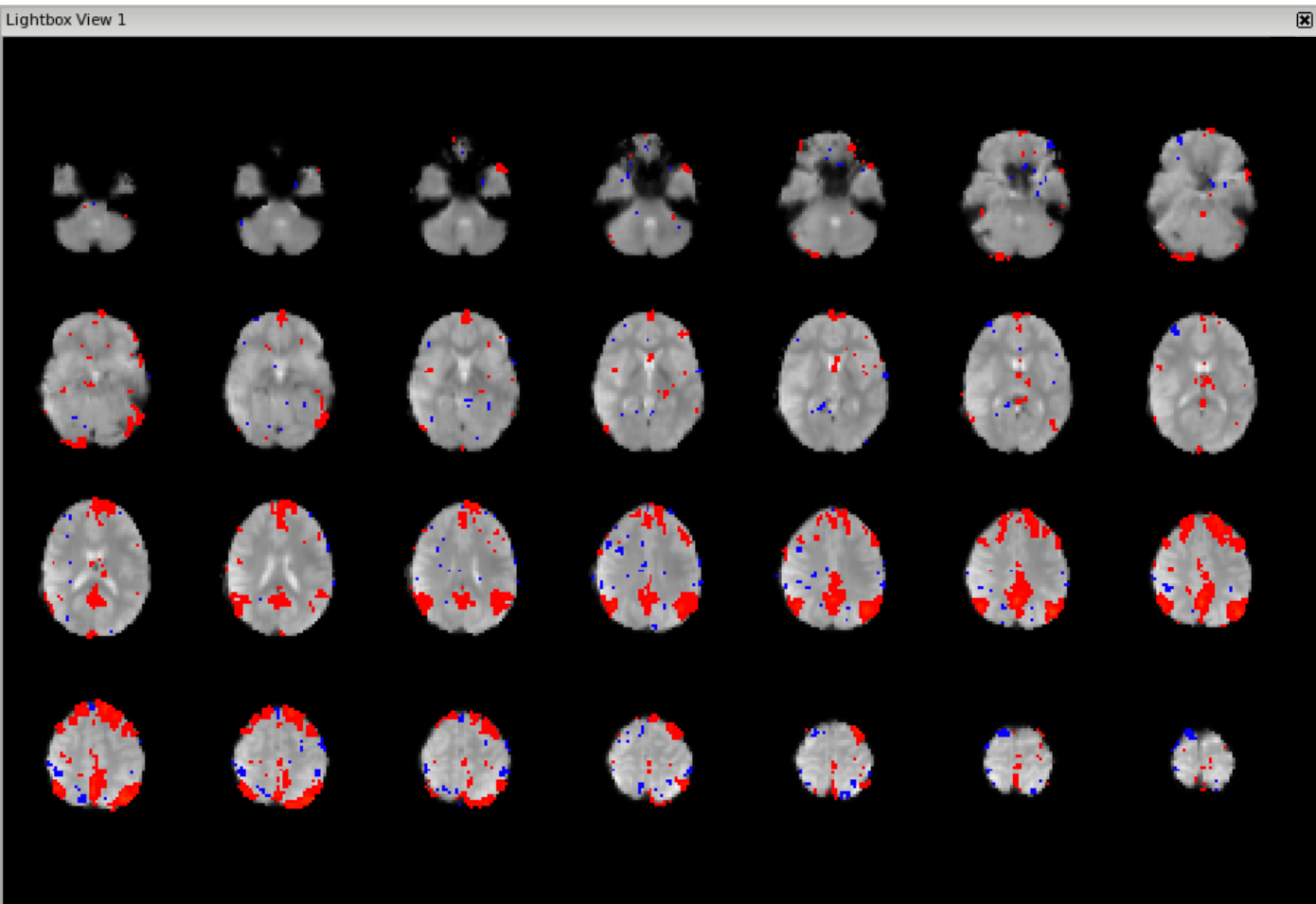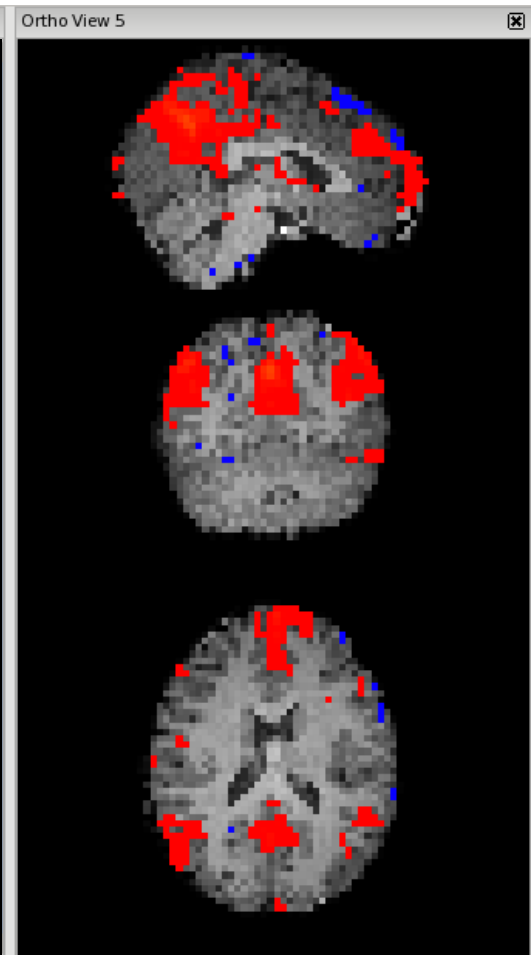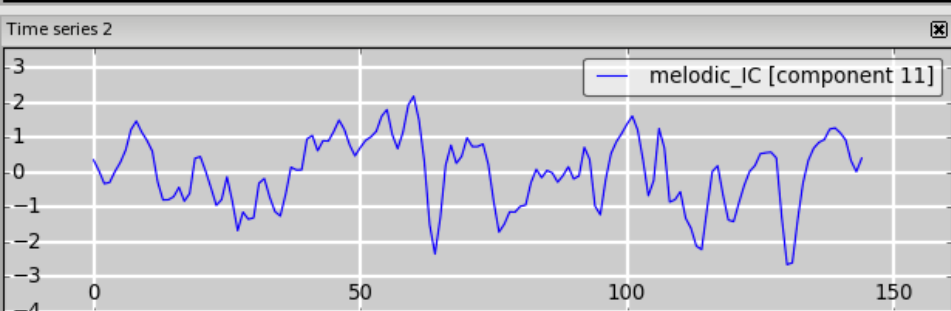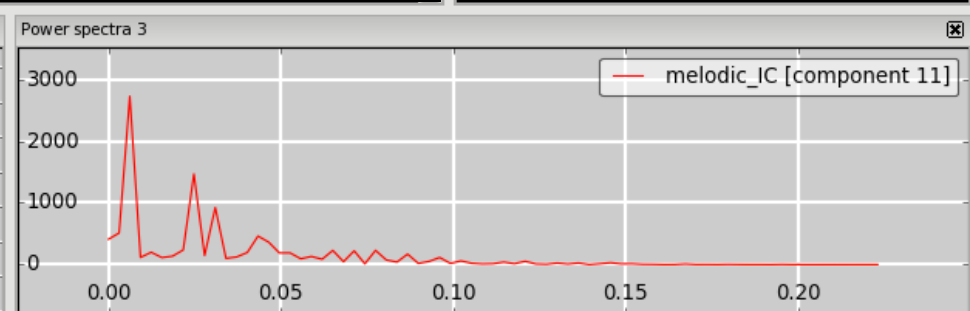

Fig S2

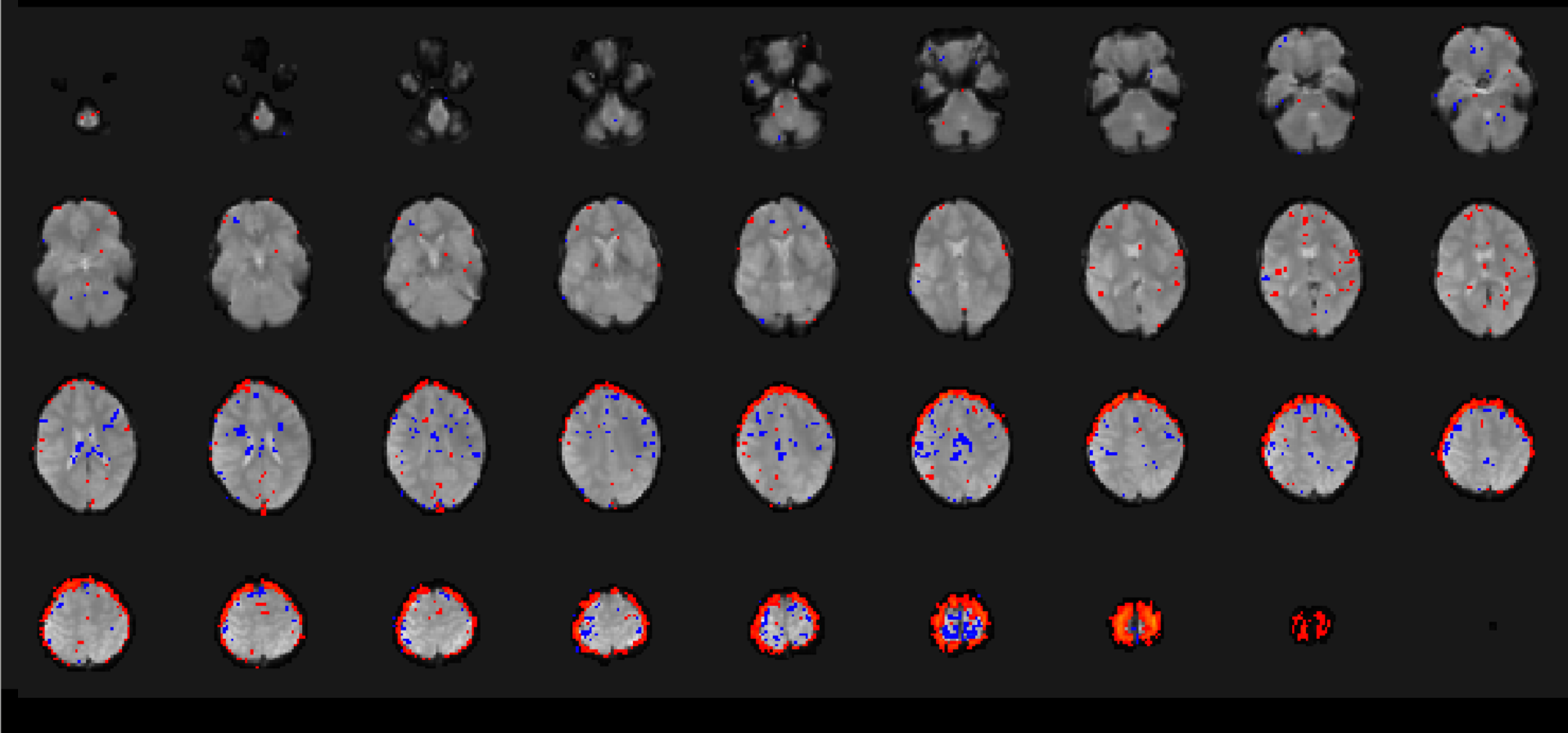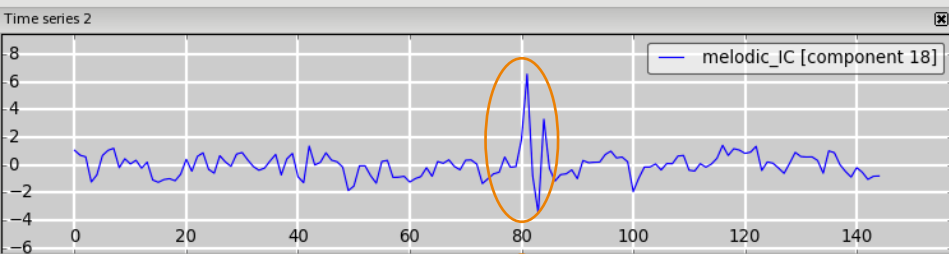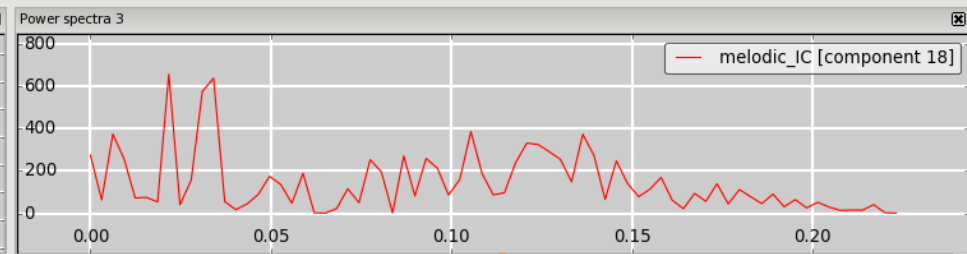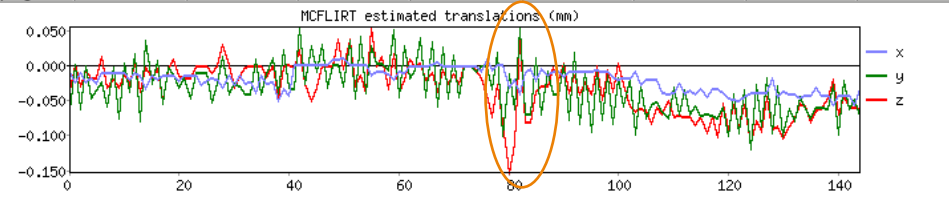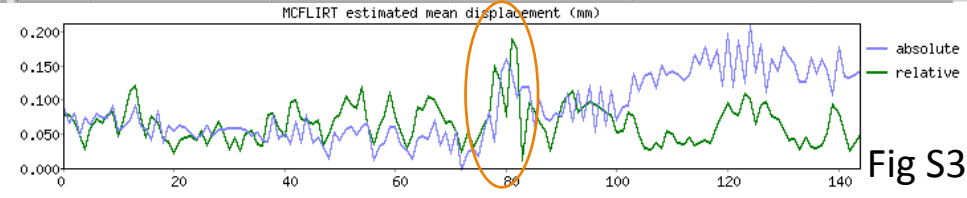

Fig S3

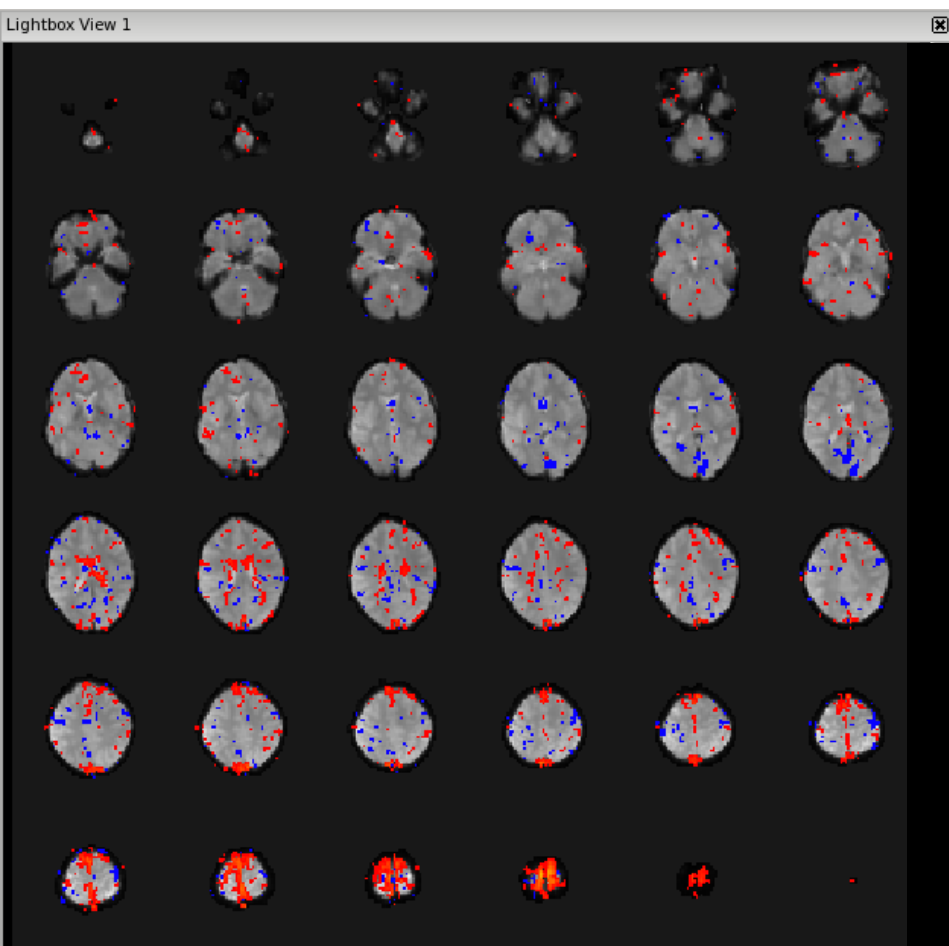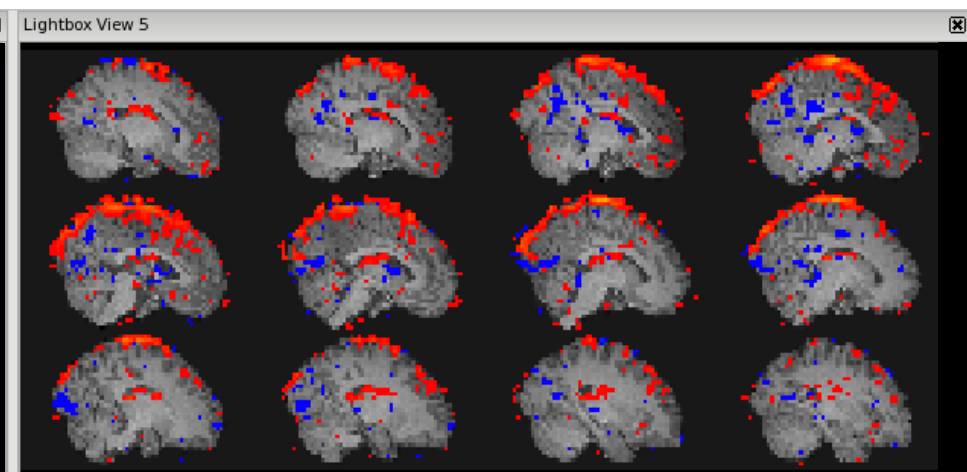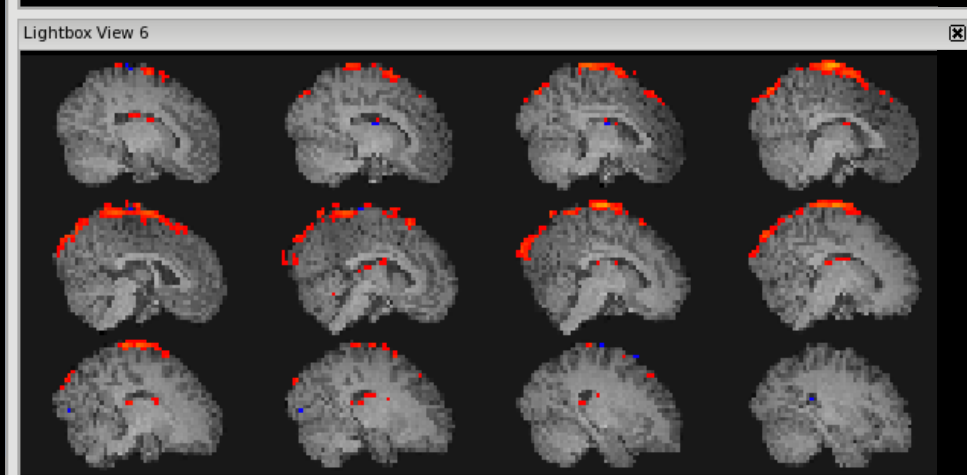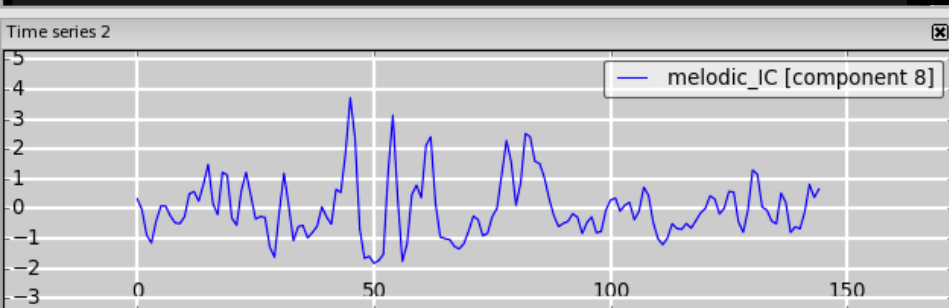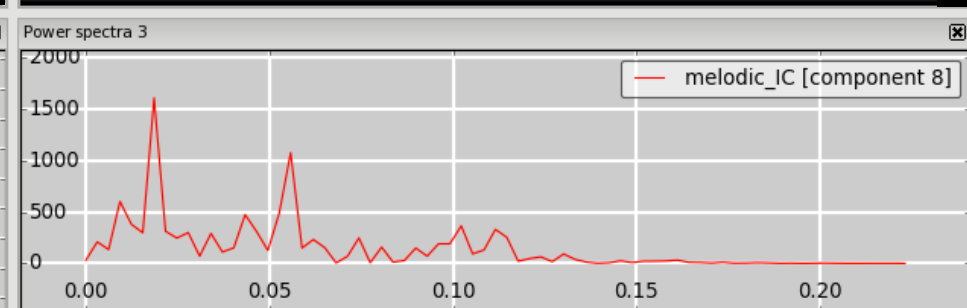

Fig S4

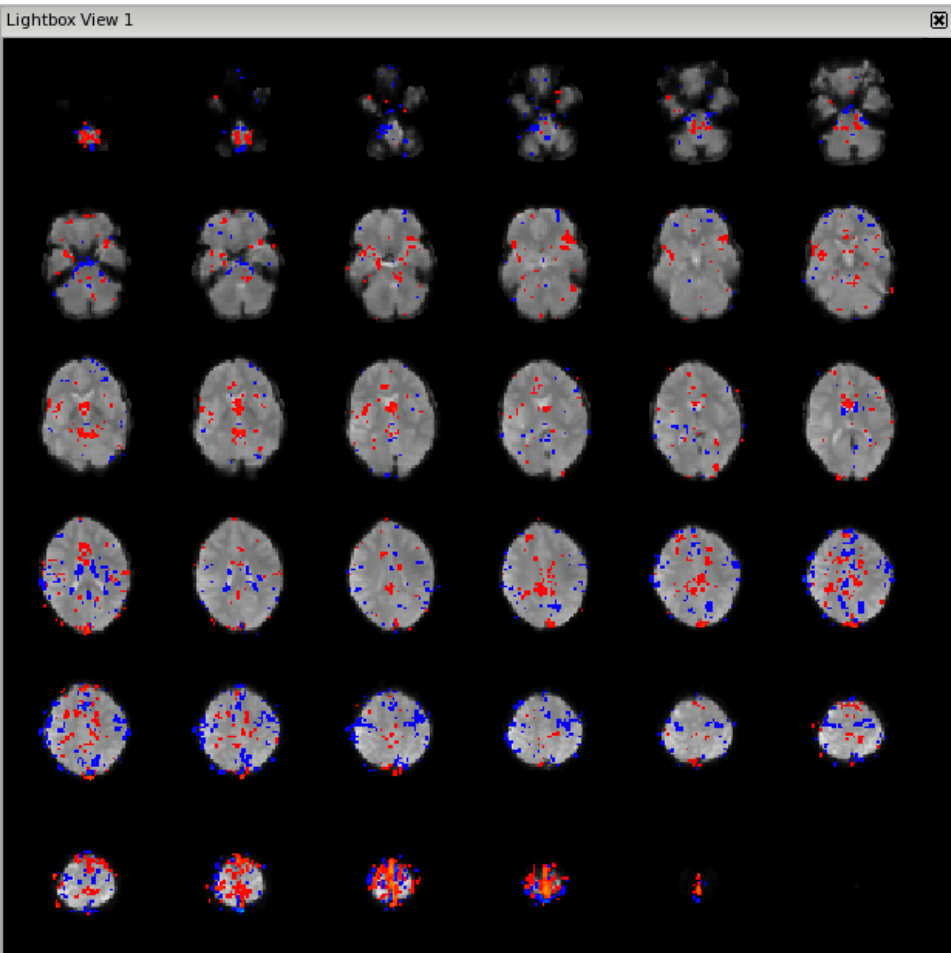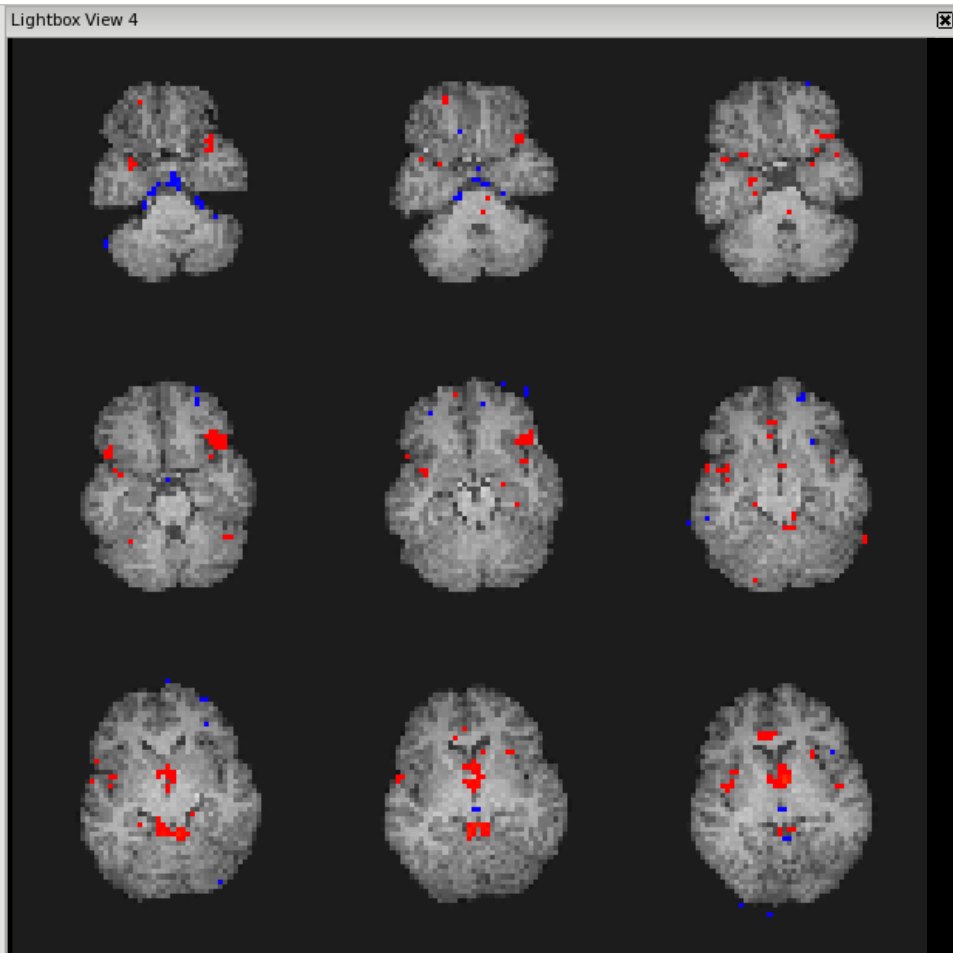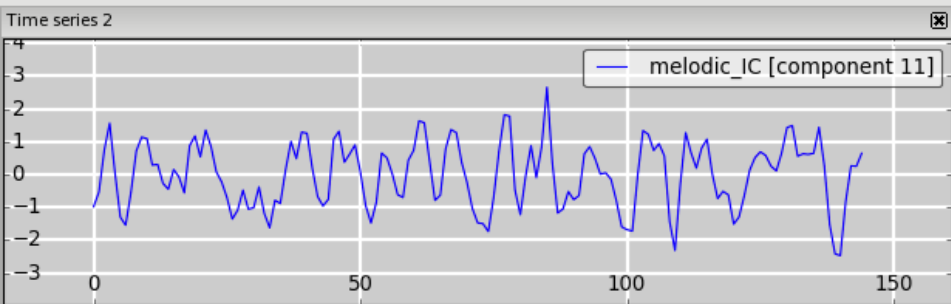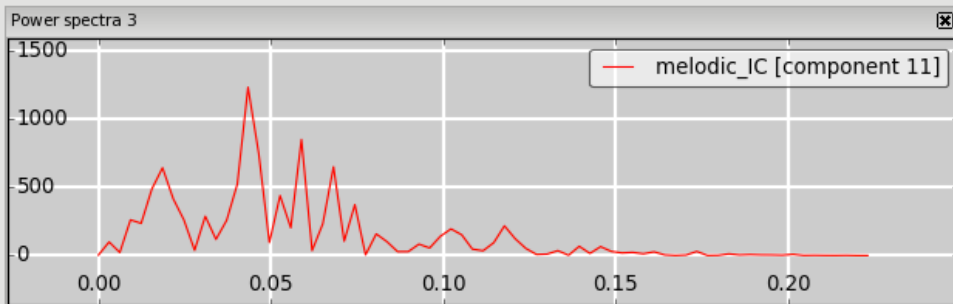

Fig S5

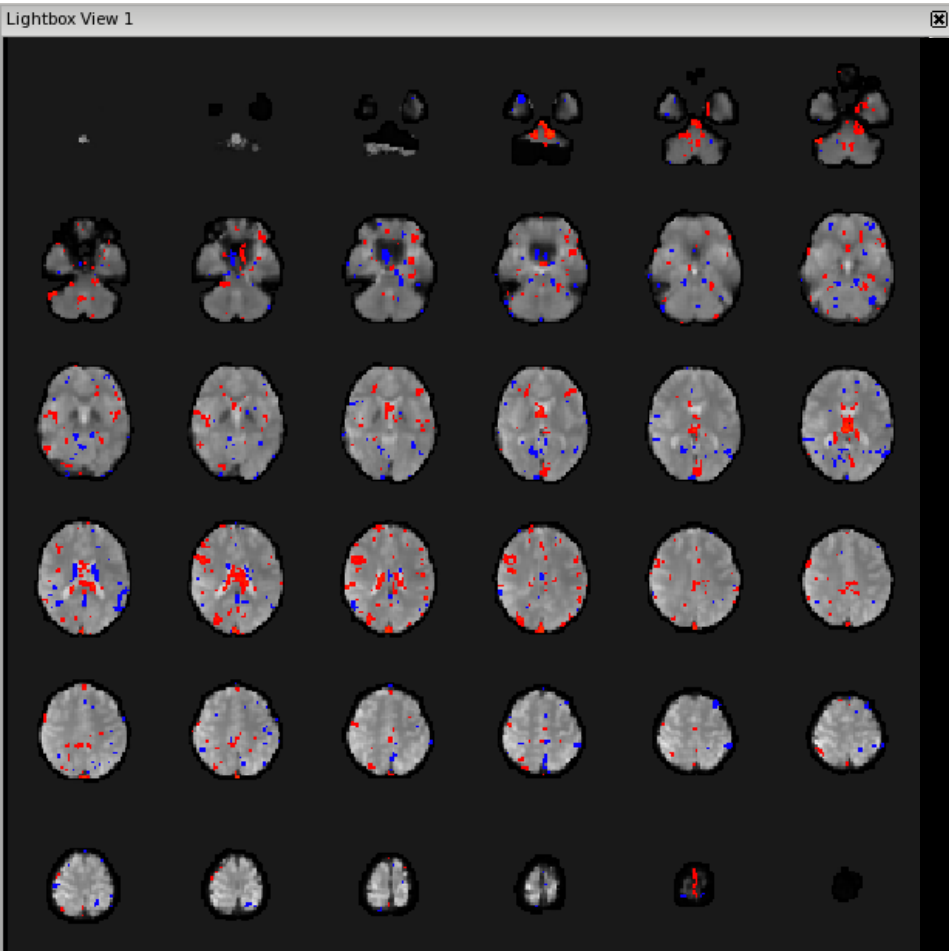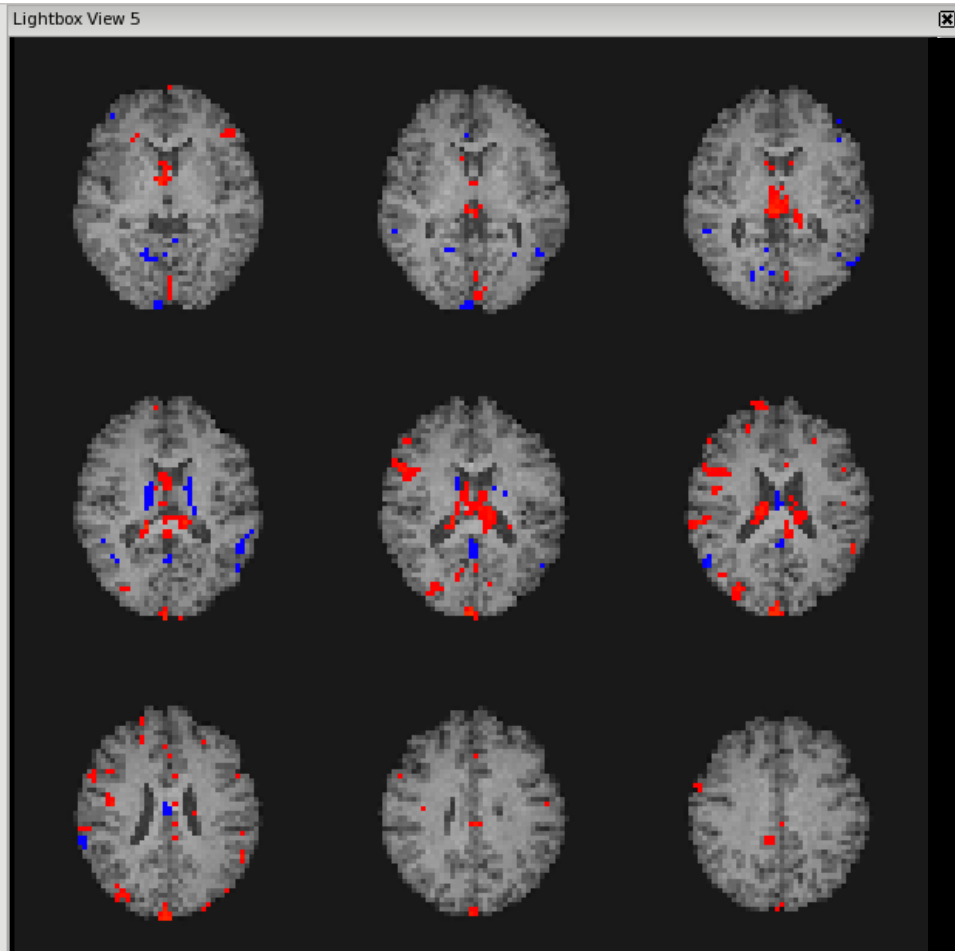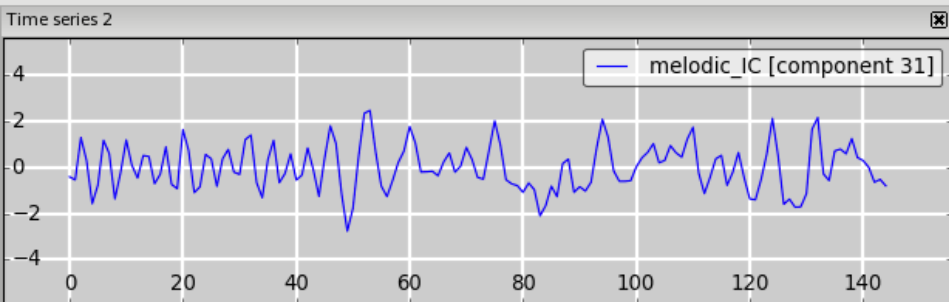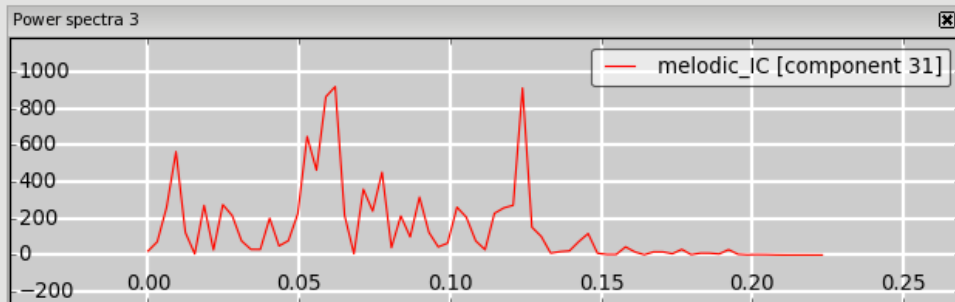

Fig S6

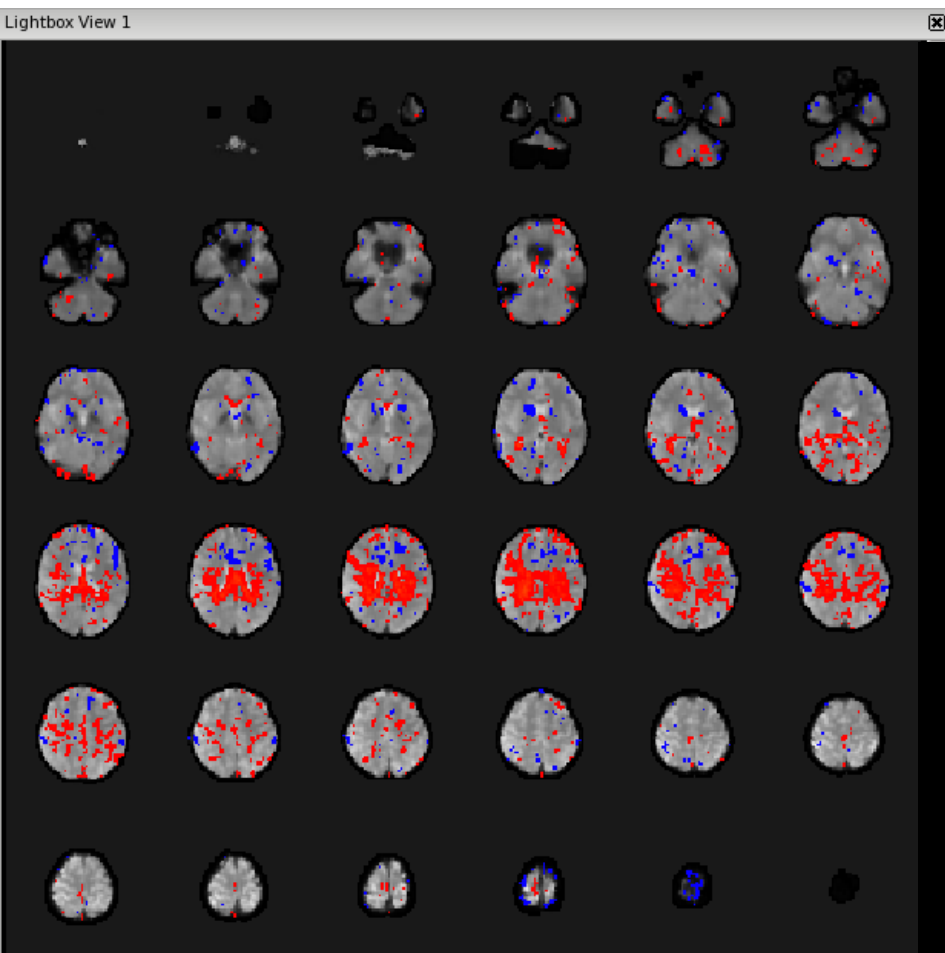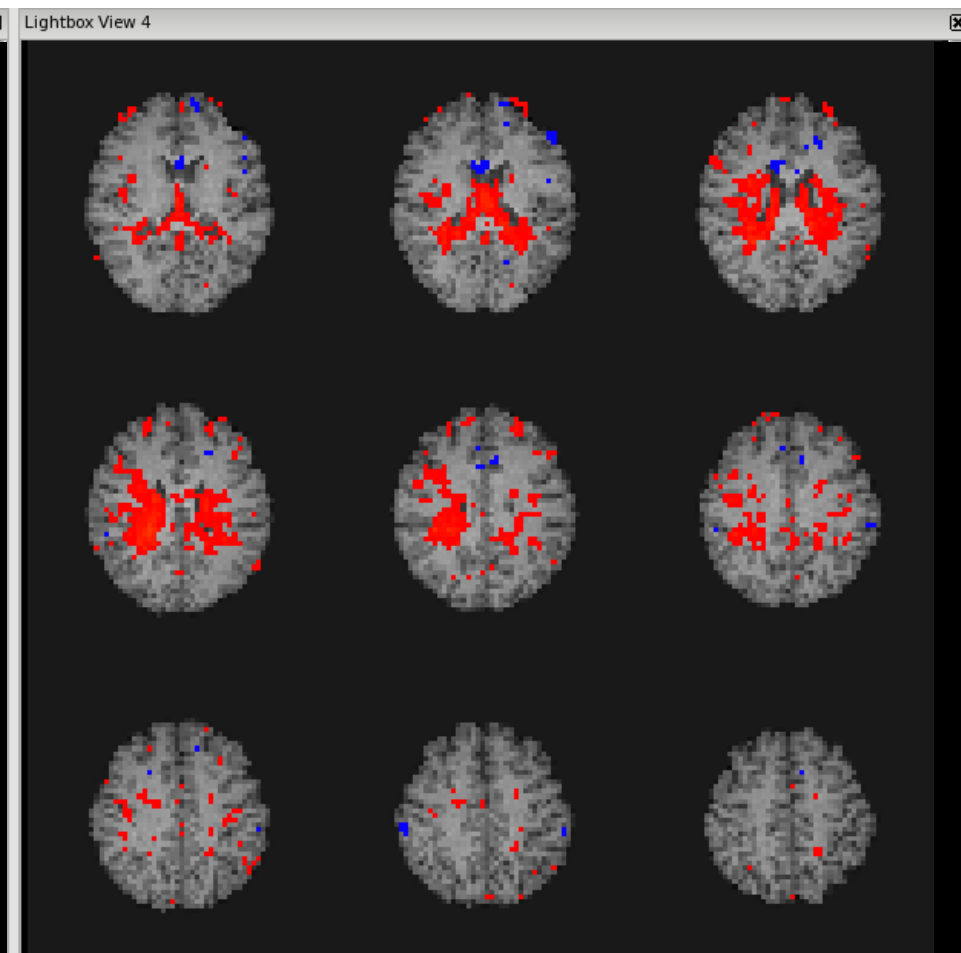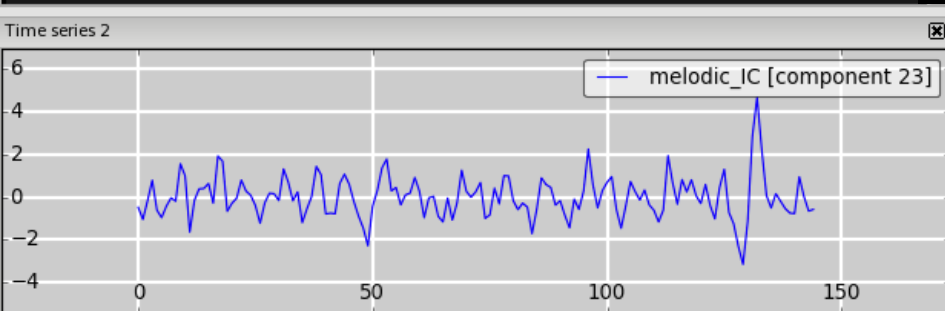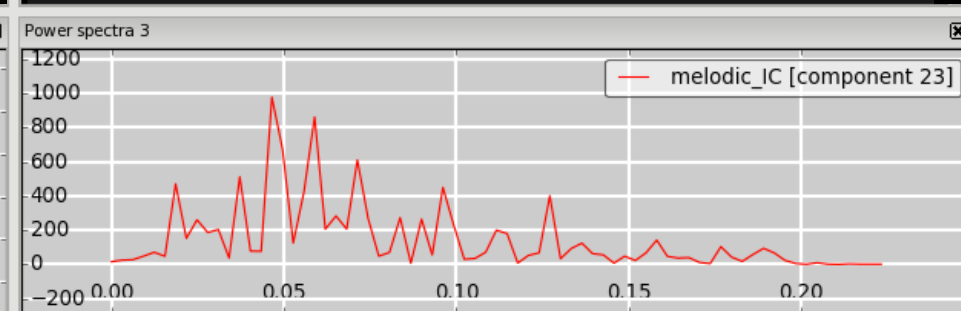

Fig S7

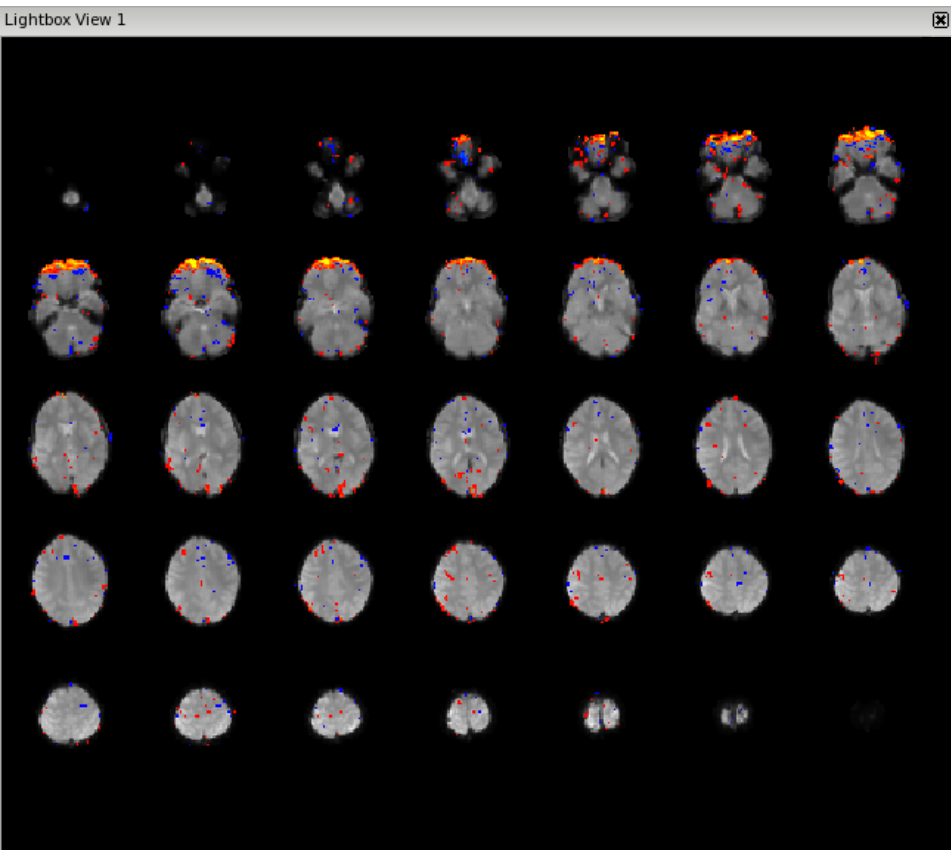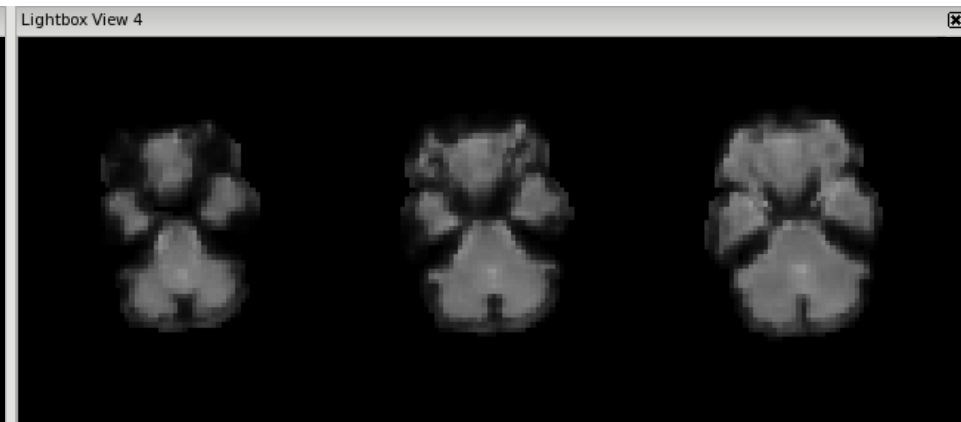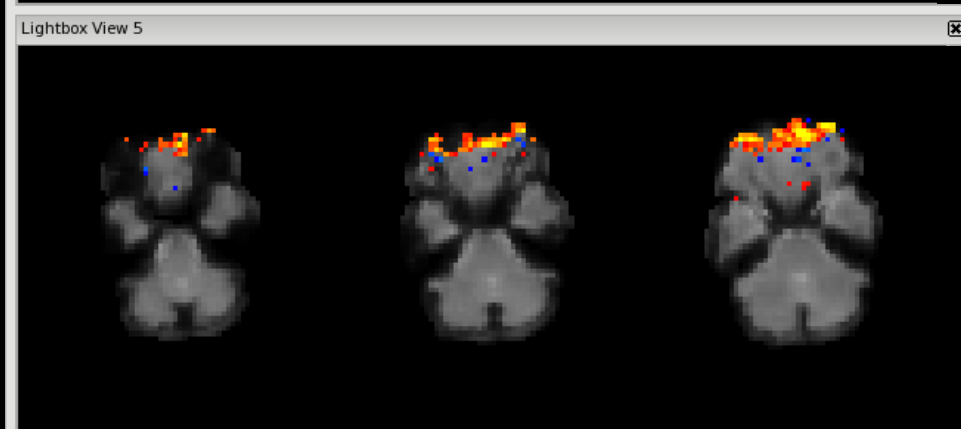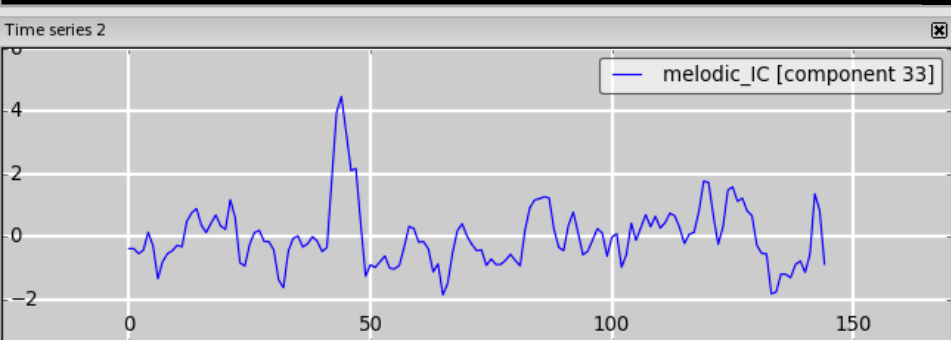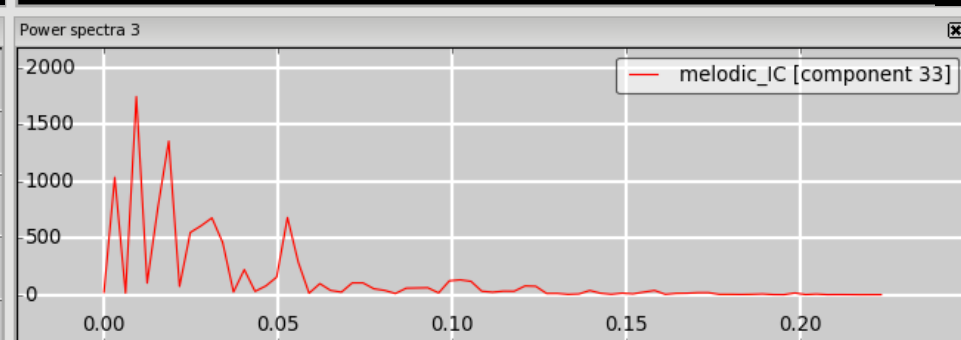

Fig S8

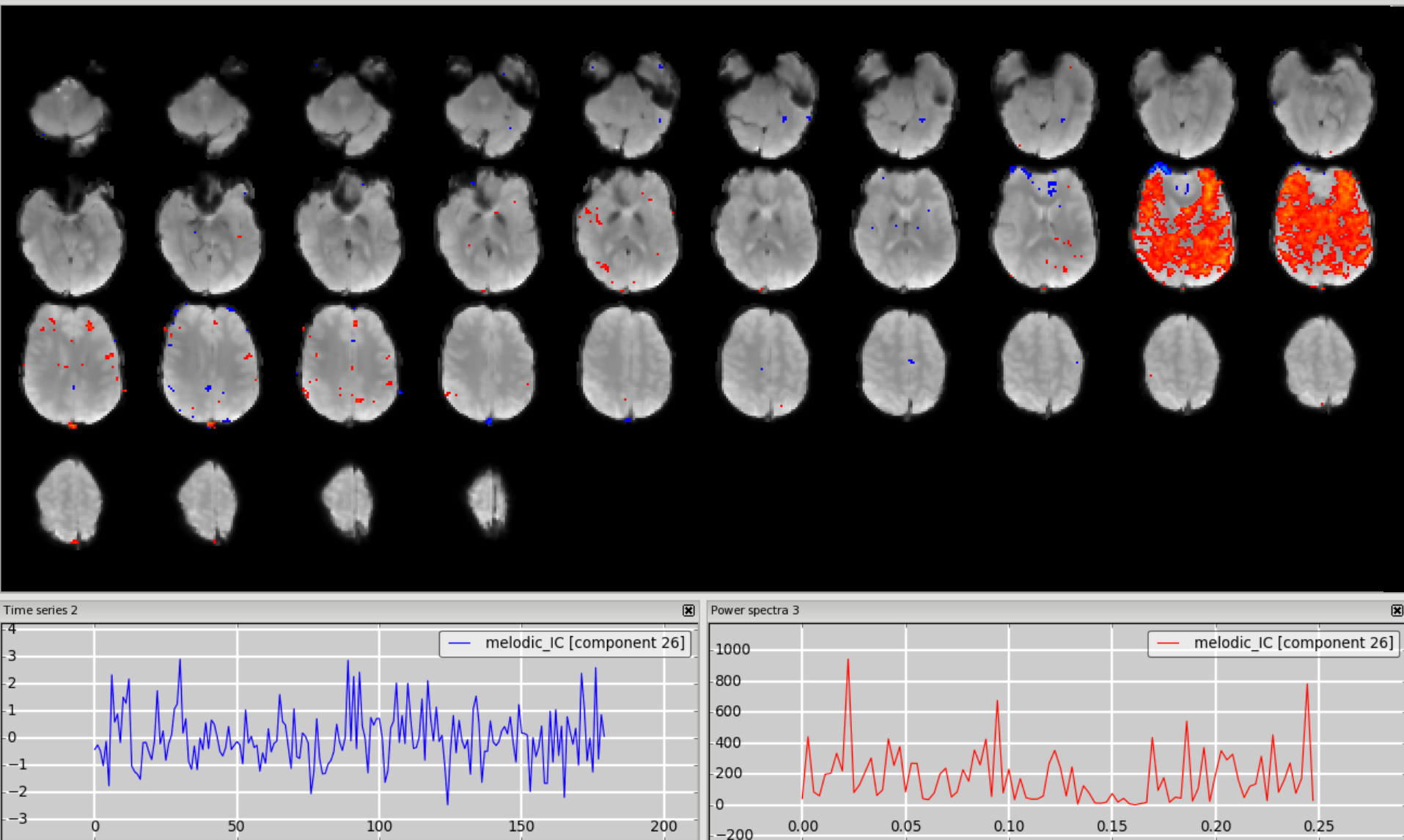

Fig S9

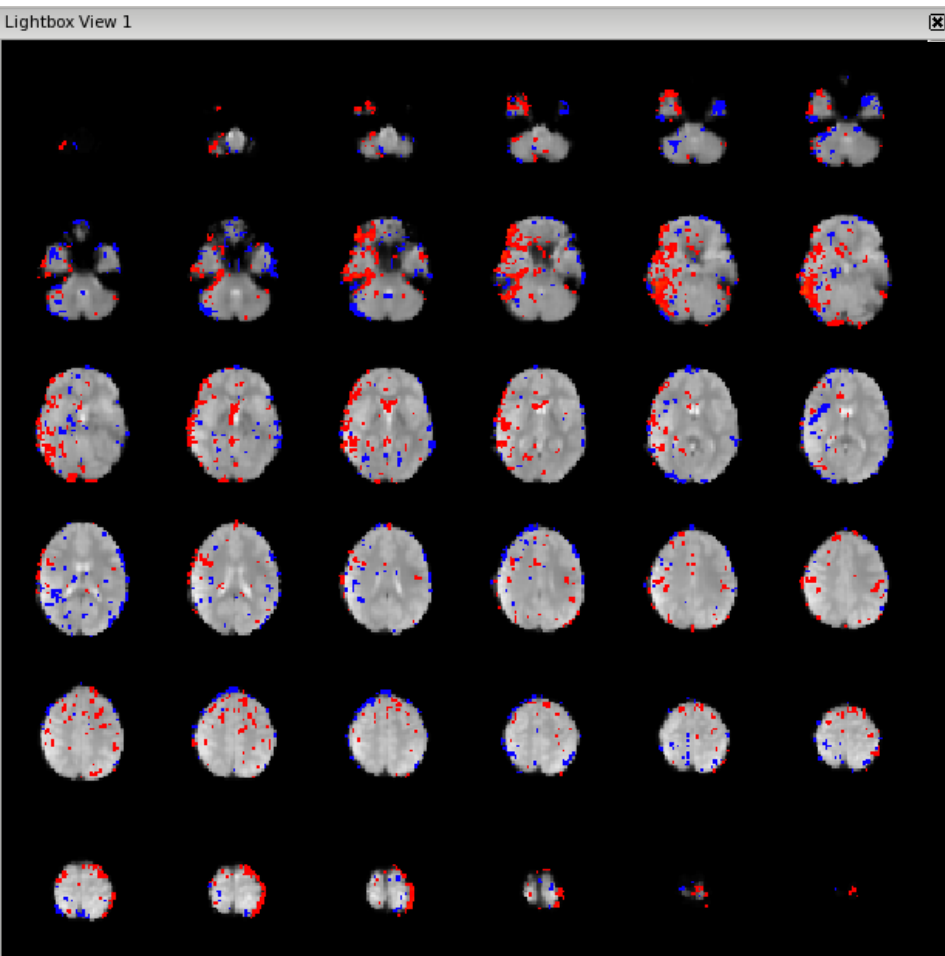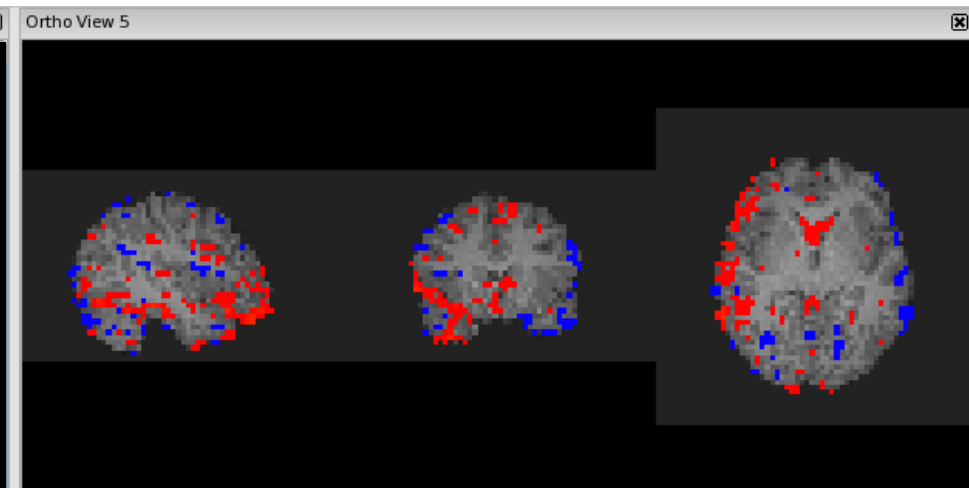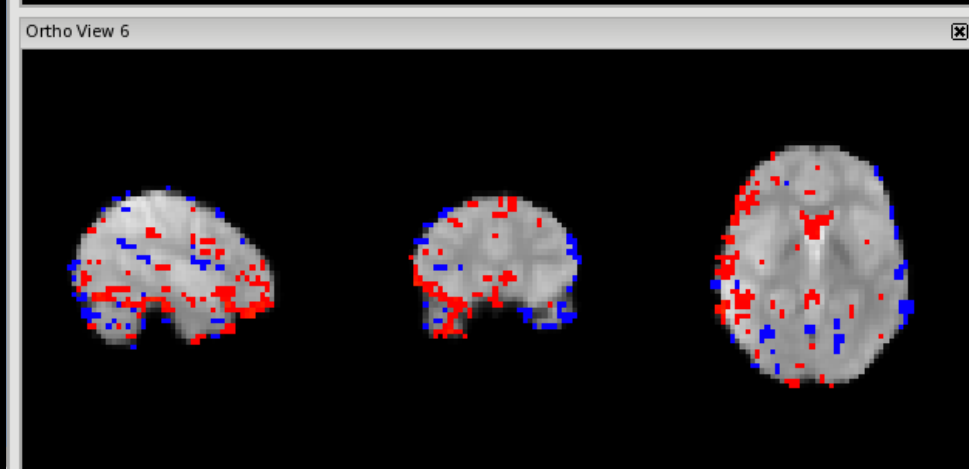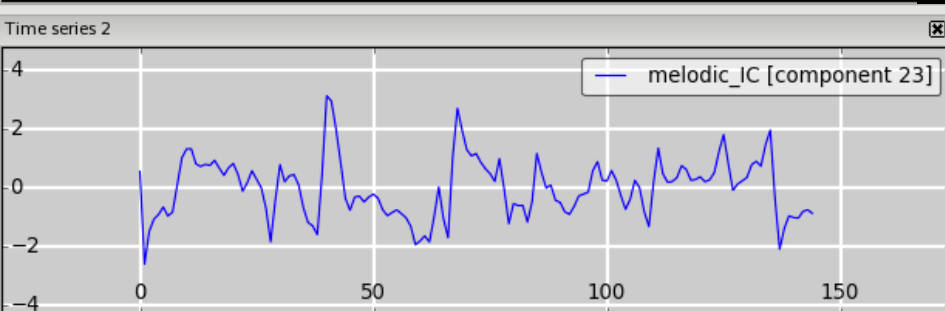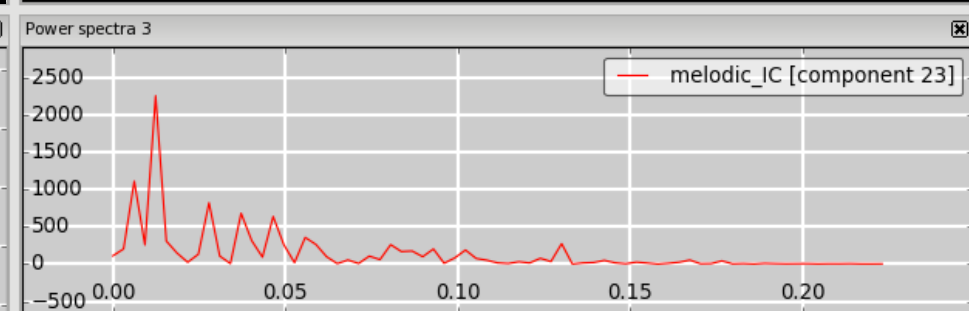

Fig S10

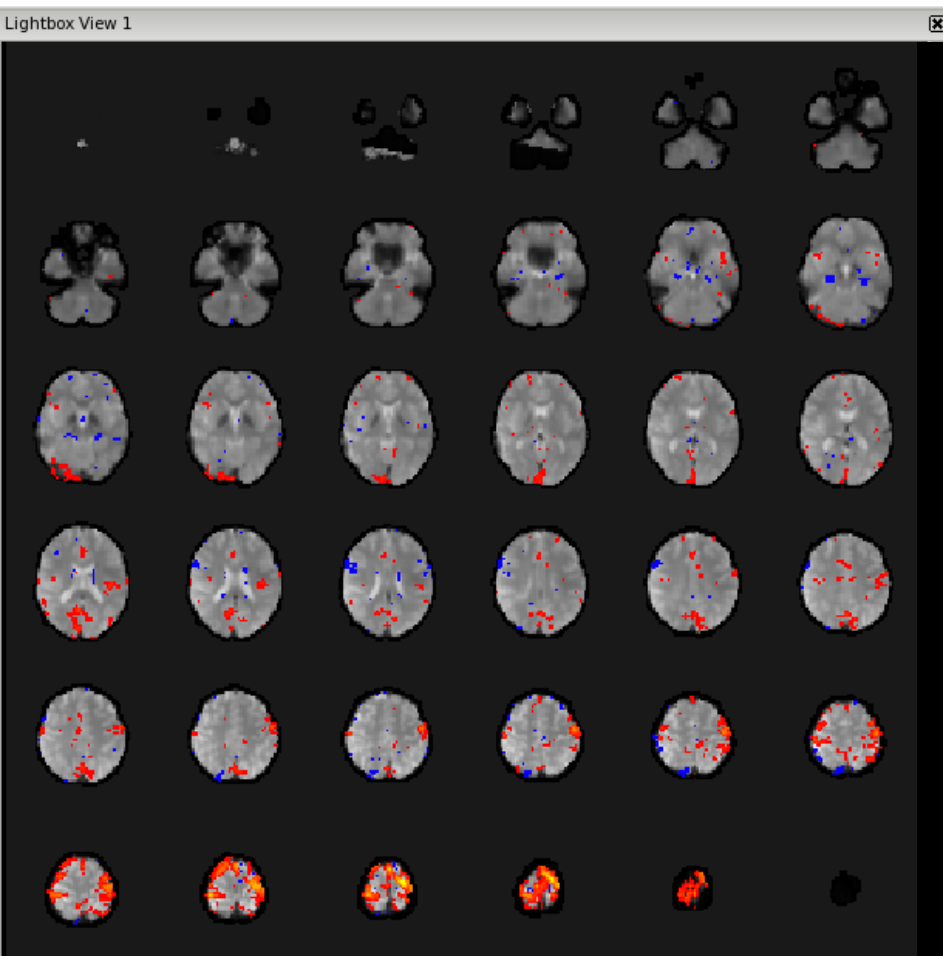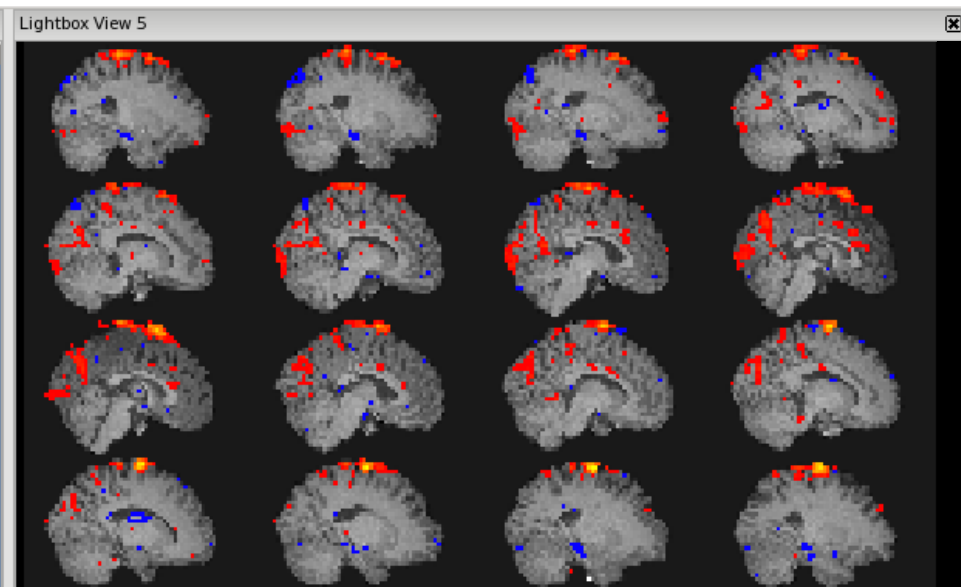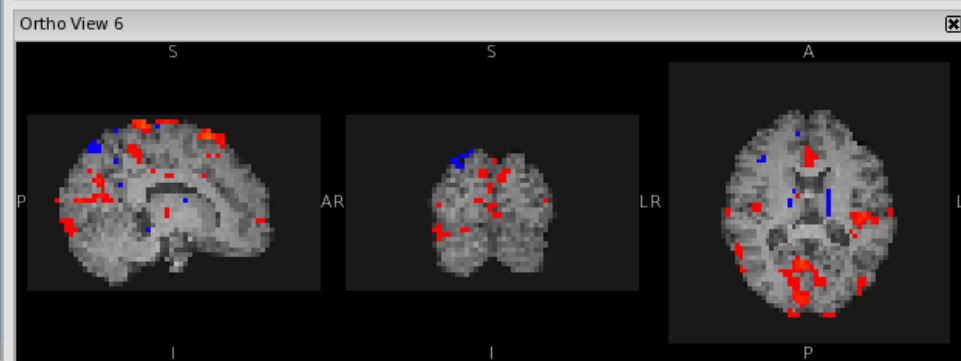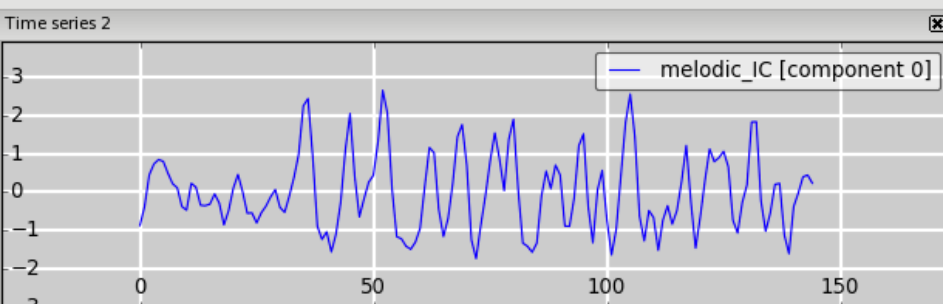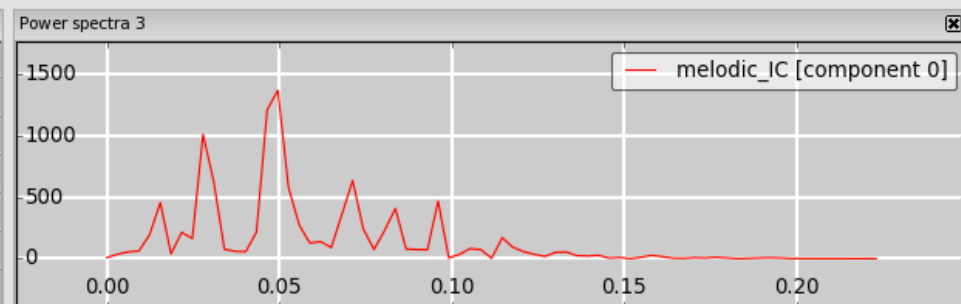

Fig S11

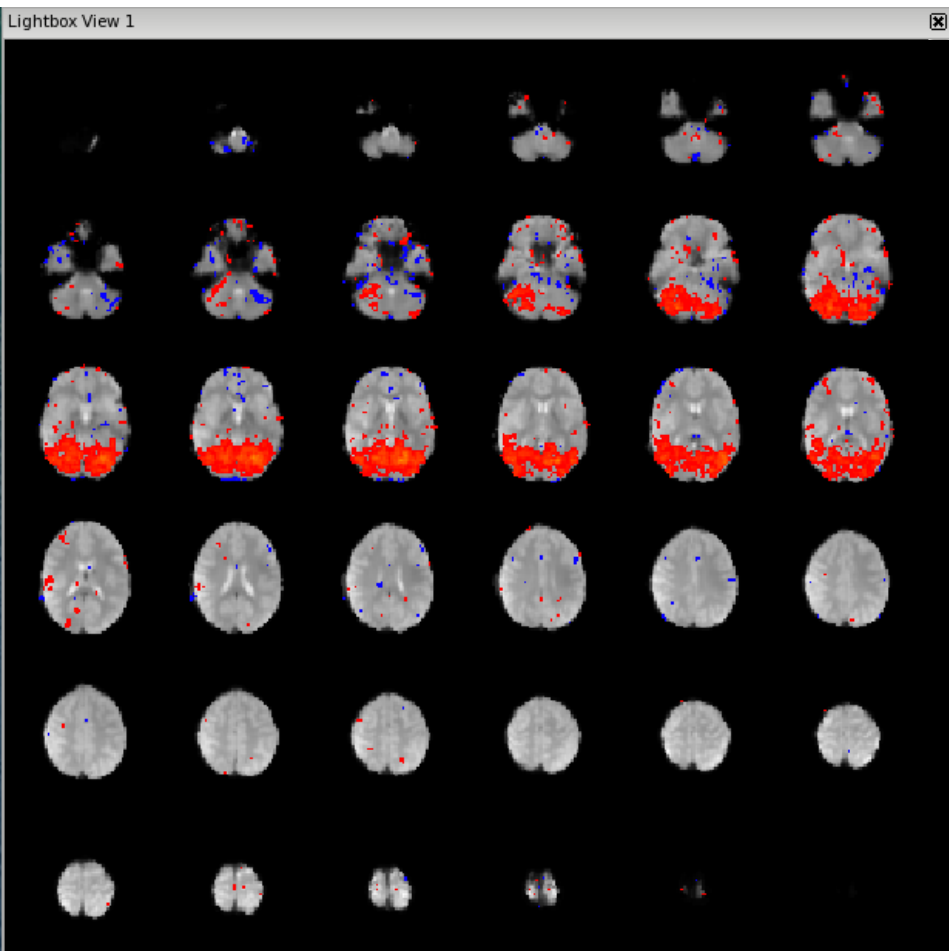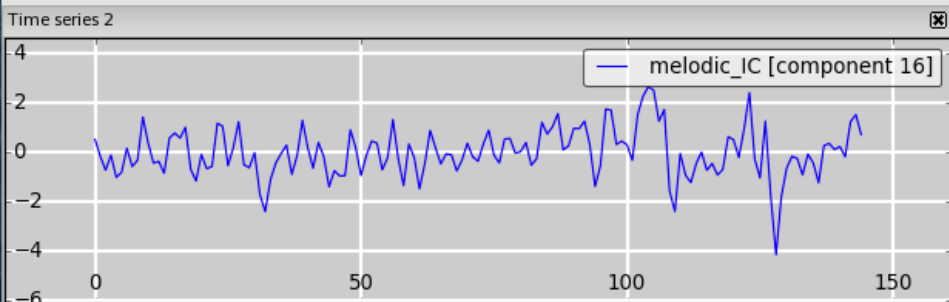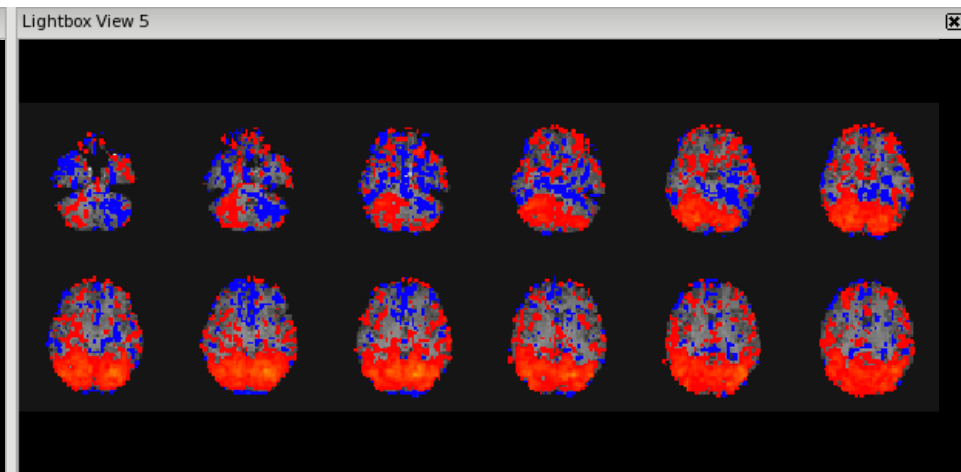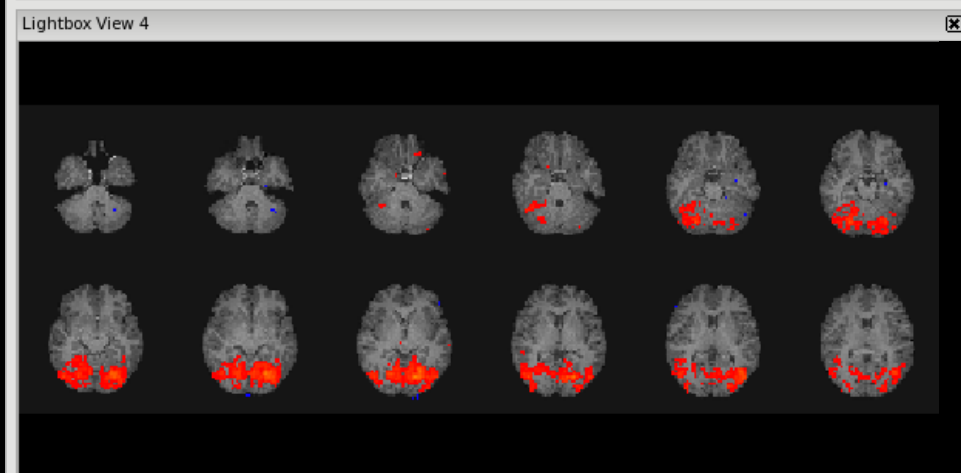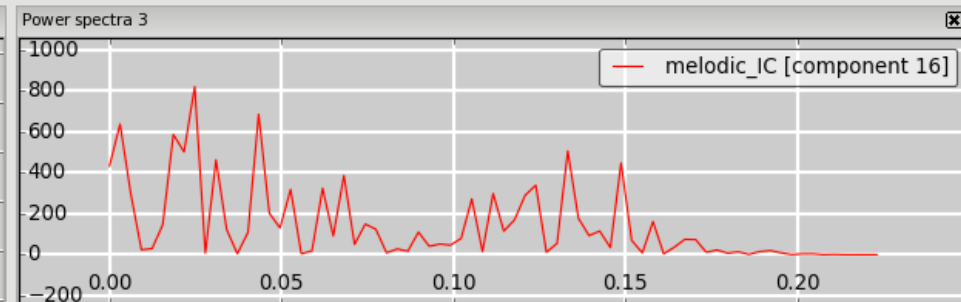

Fig S12

Supplement: Supplementary file 4 — Supplementary material [file mmc4.pdf]
