# Supplementary material for: Hand classification of fMRI ICA noise components
Source: Neuroimage. 2017 Jul 1;154:188–205. doi: 10.1016/j.neuroimage.2016.12.036 (PMC5489418; doi:10.1016/j.neuroimage.2016.12.036)
Supplement: Supplementary file 2 — Supplementary material [file mmc2.pdf]

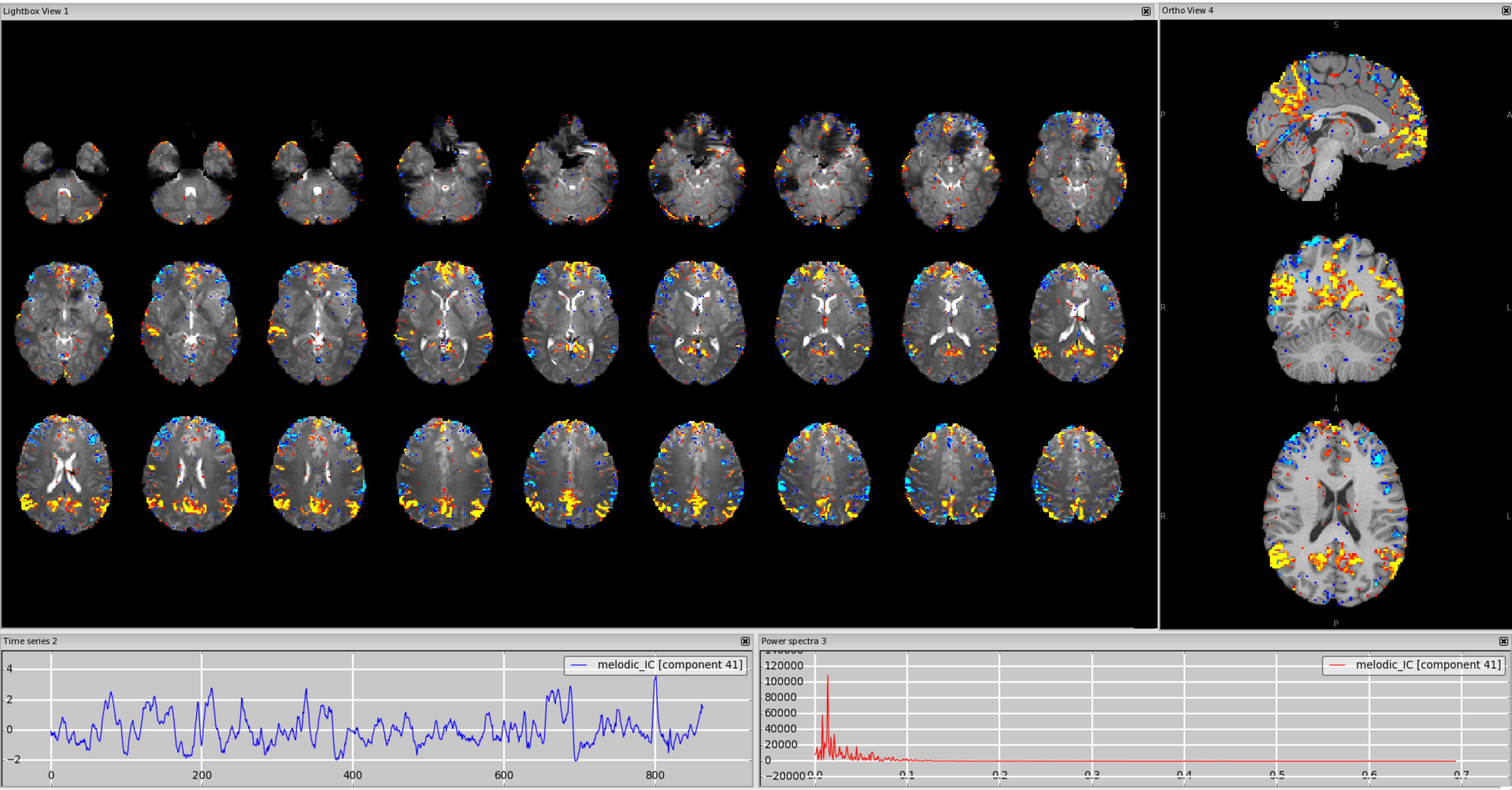
Lightbox View 1
Ortho View 4
Time series 2
melodic_IC [component 41]
4
2
0
−2
0
200
400
600
800
Power spectra 3
melodic_IC [component 41]
120000
100000
80000
60000
40000
20000
0
−20000
0.0
0.1
0.2
0.3
0.4
0.5
0.6
0.7

Fig S13

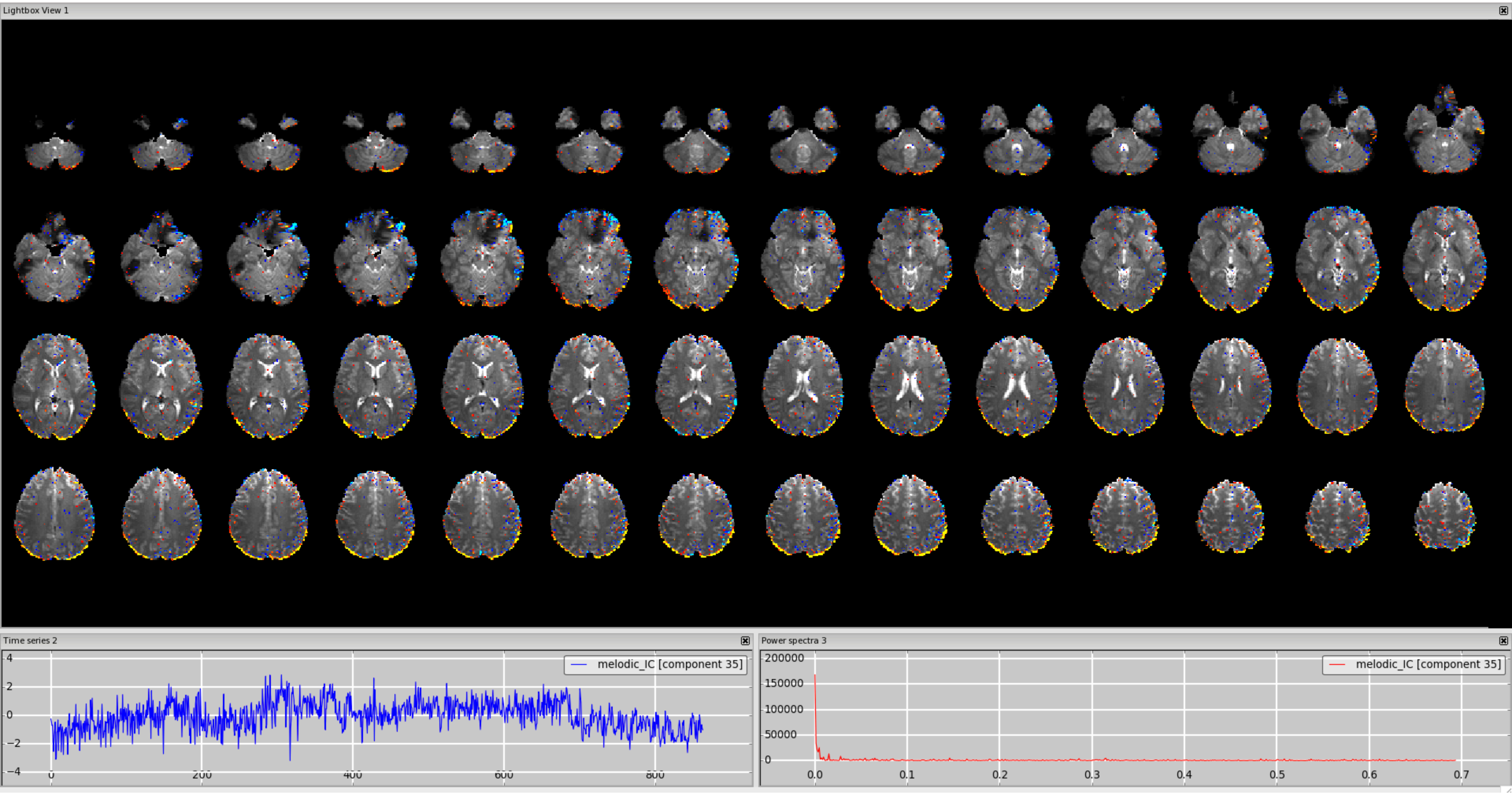
Lightbox View 1
Time series 2
melodic_IC [component 35]
4
2
0
−2
−4
0
200
400
600
800
Power spectra 3
melodic_IC [component 35]
200000
150000
100000
50000
0
0.0
0.1
0.2
0.3
0.4
0.5
0.6
0.7

Fig S14

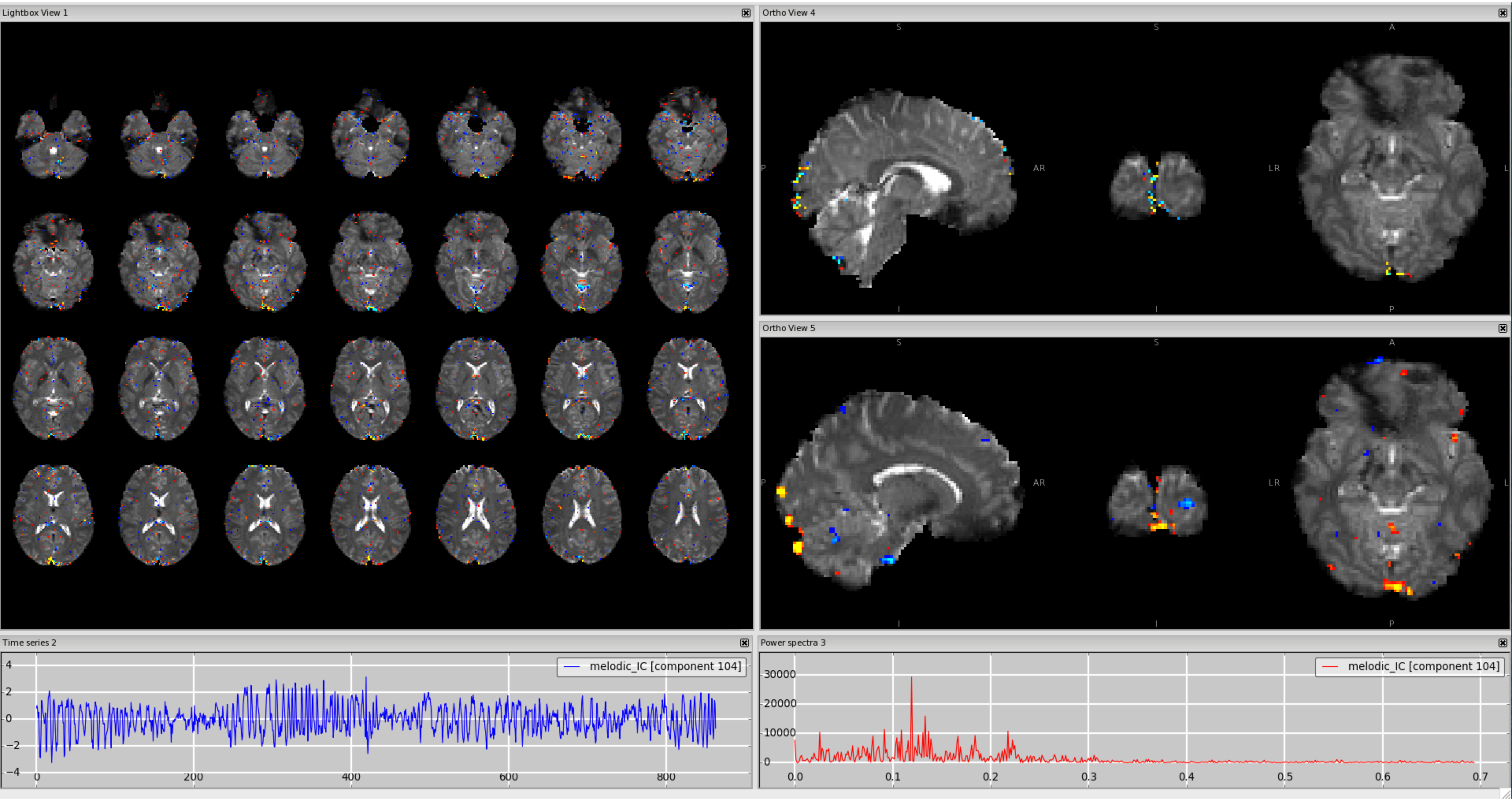
Lightbox View 1
Ortho View 4
Ortho View 5
Time series 2
melodic_IC [component 104]
Power spectra 3
melodic_IC [component 104]

Fig S15

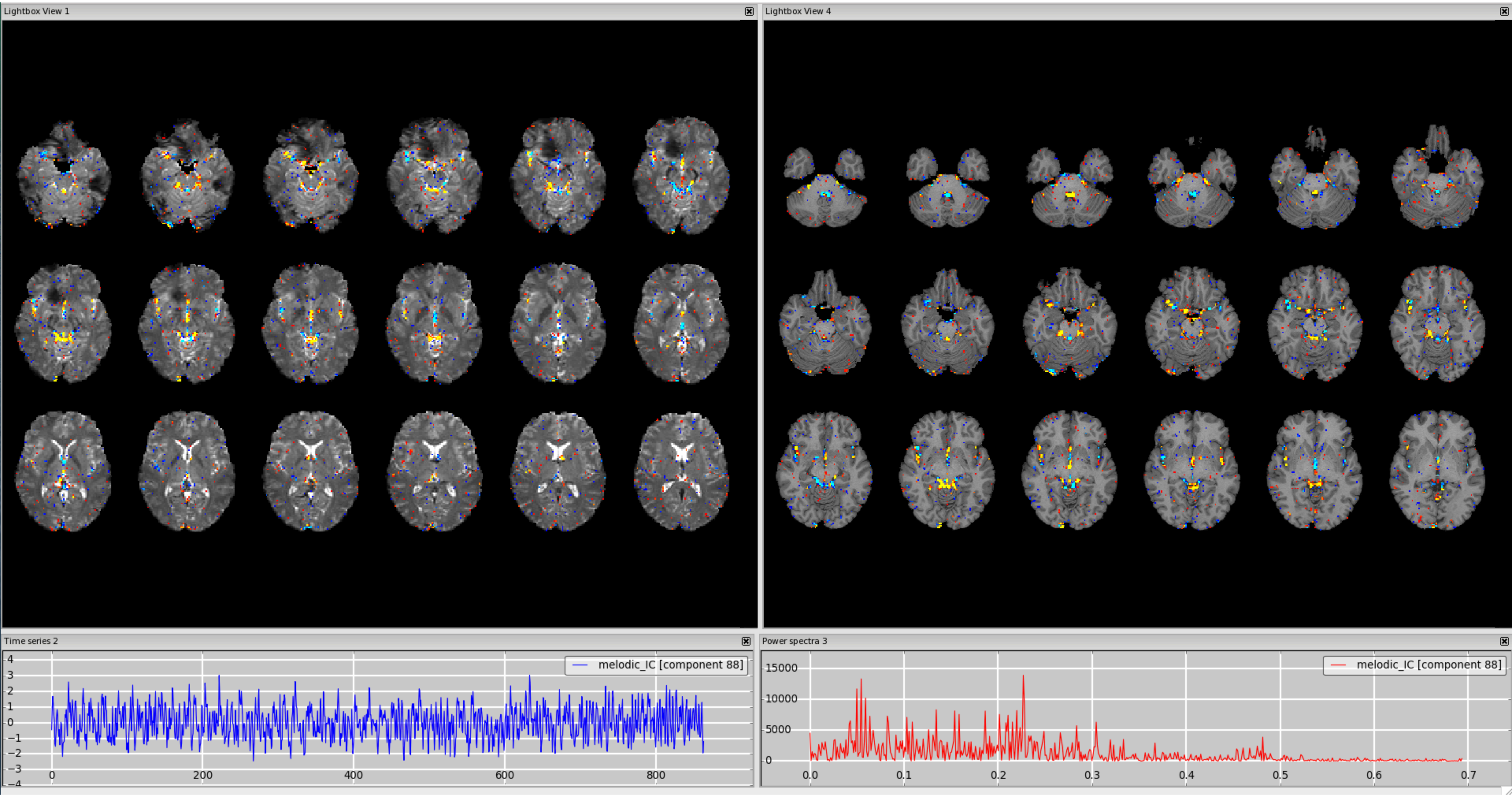

Lightbox View 1
Lightbox View 4
Time series 2
melodic_IC [component 88]
4
3
2
1
0
−1
−2
−3
−4
0
200
400
600
800
Power spectra 3
melodic_IC [component 88]
15000
10000
5000
0
0.0
0.1
0.2
0.3
0.4
0.5
0.6
0.7

Fig S16

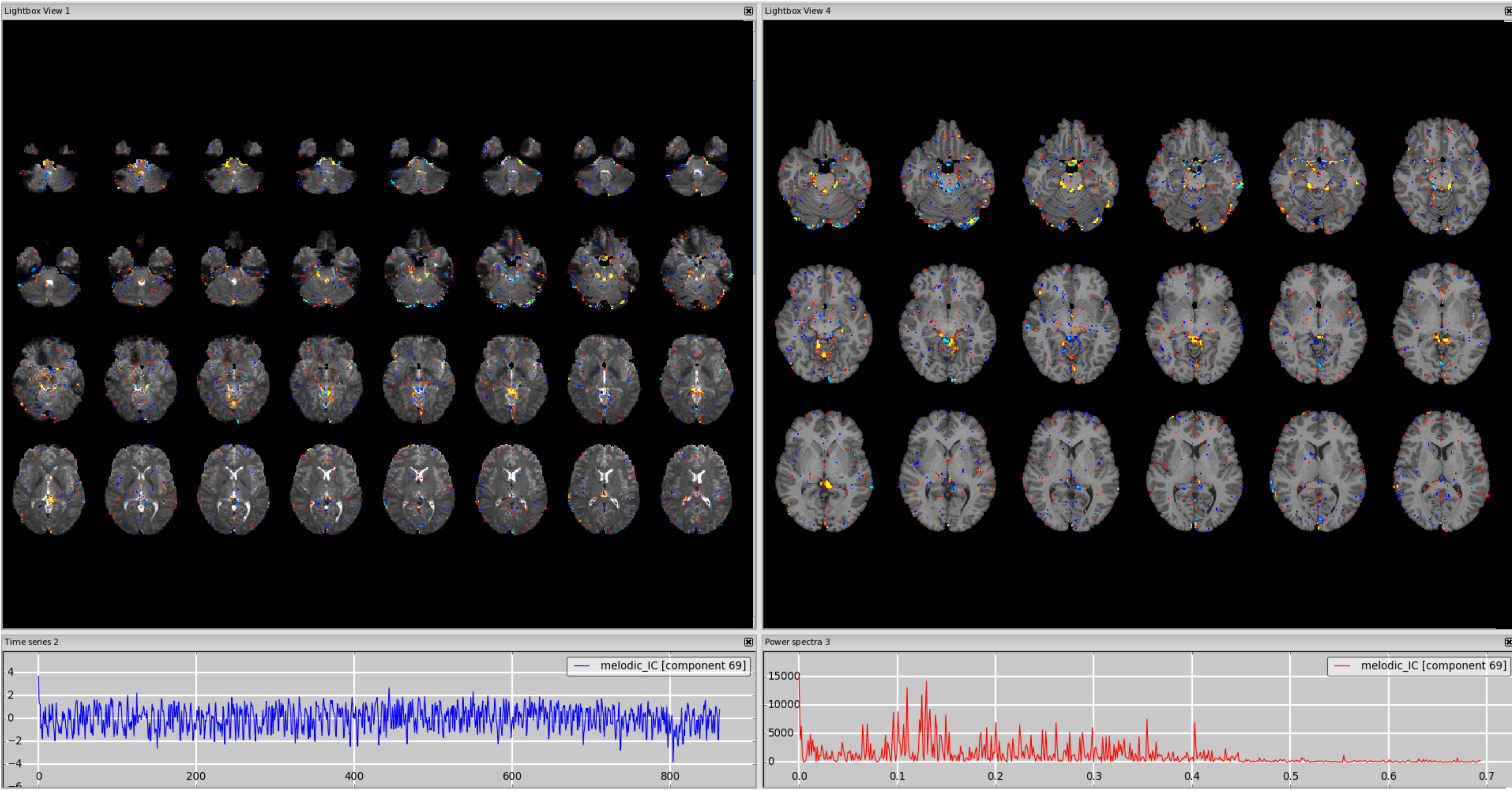
Lightbox View 1
Lightbox View 4
Time series 2
melodic_IC [component 69]
4
2
0
−2
−4
0
200
400
600
800
Power spectra 3
melodic_IC [component 69]
15000
10000
5000
0
0.0
0.1
0.2
0.3
0.4
0.5
0.6
0.7

Fig S17

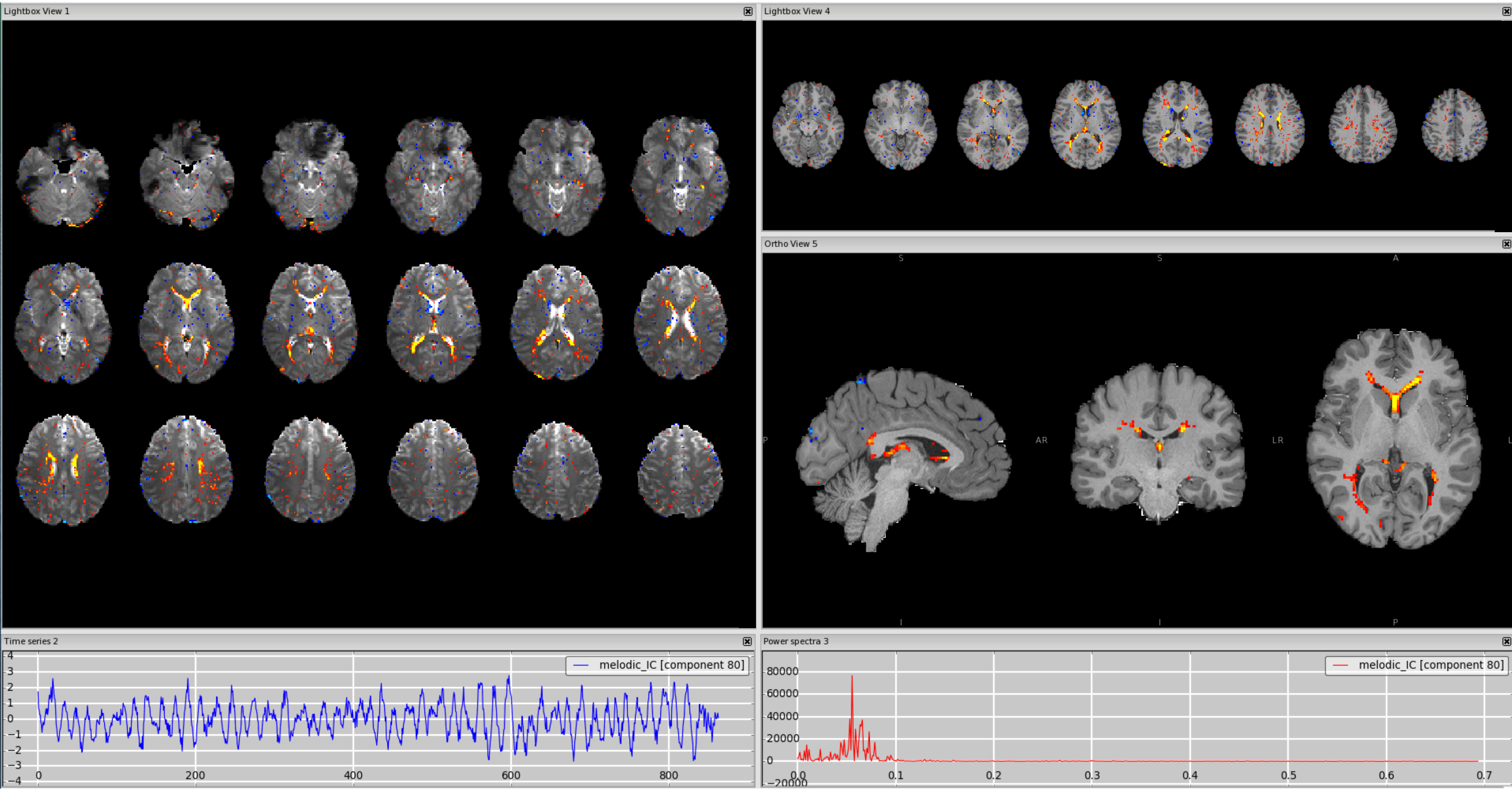

Lightbox View 1
Lightbox View 4
Ortho View 5
Time series 2
melodic_IC [component 80]
Power spectra 3
melodic_IC [component 80]

Fig S18

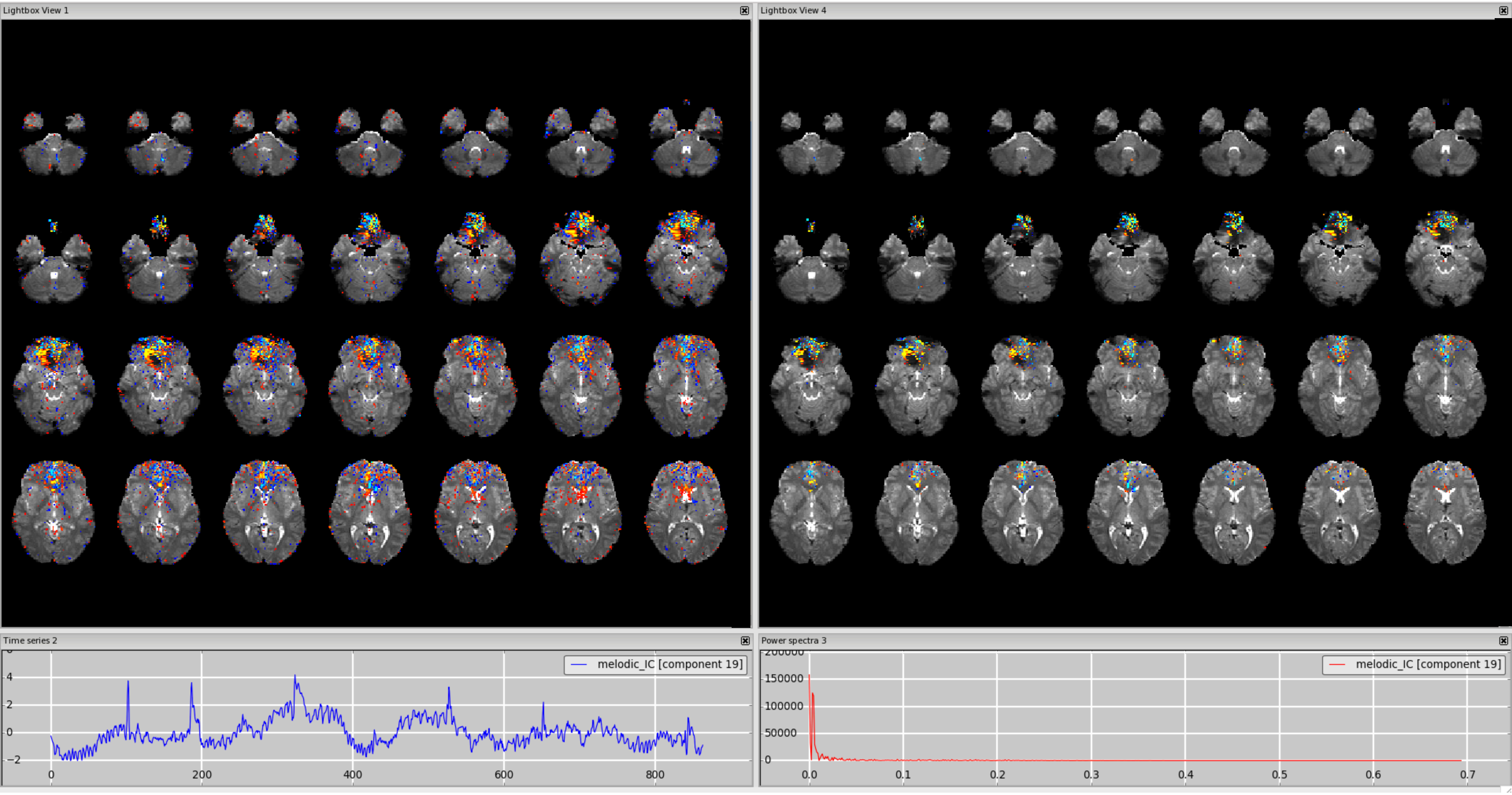

Lightbox View 1
Lightbox View 4
Time series 2
melodic_IC [component 19]
4
2
0
-2
0
200
400
600
800
Power spectra 3
melodic_IC [component 19]
200000
150000
100000
50000
0
0.0
0.1
0.2
0.3
0.4
0.5
0.6
0.7

Fig S19

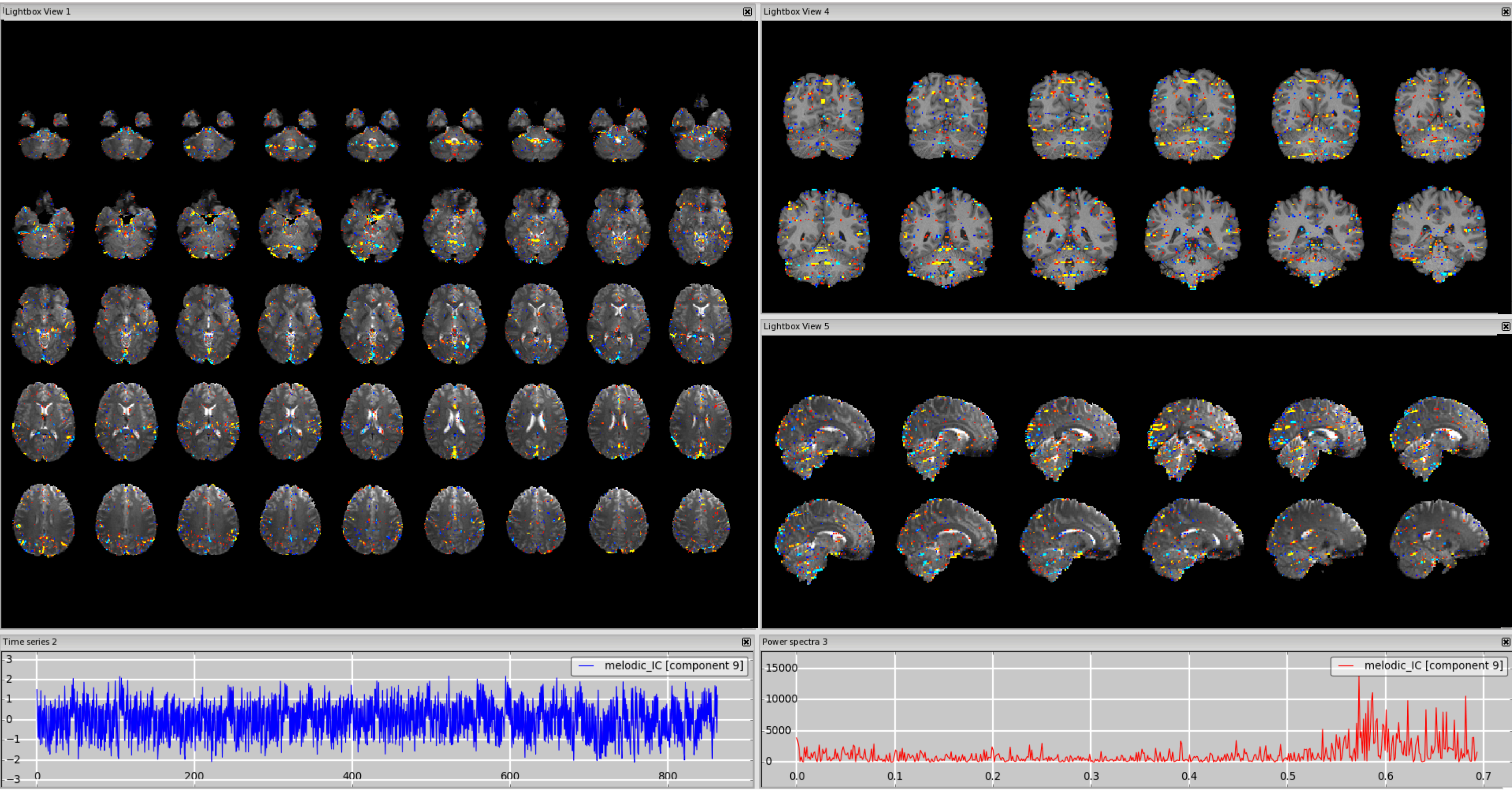

Lightbox View 1
Lightbox View 4
Lightbox View 5
Time series 2
melodic_IC [component 9]
3
2
1
0
-1
-2
-3
0
200
400
600
800
Power spectra 3
melodic_IC [component 9]
15000
10000
5000
0
0.0
0.1
0.2
0.3
0.4
0.5
0.6
0.7

Fig S20

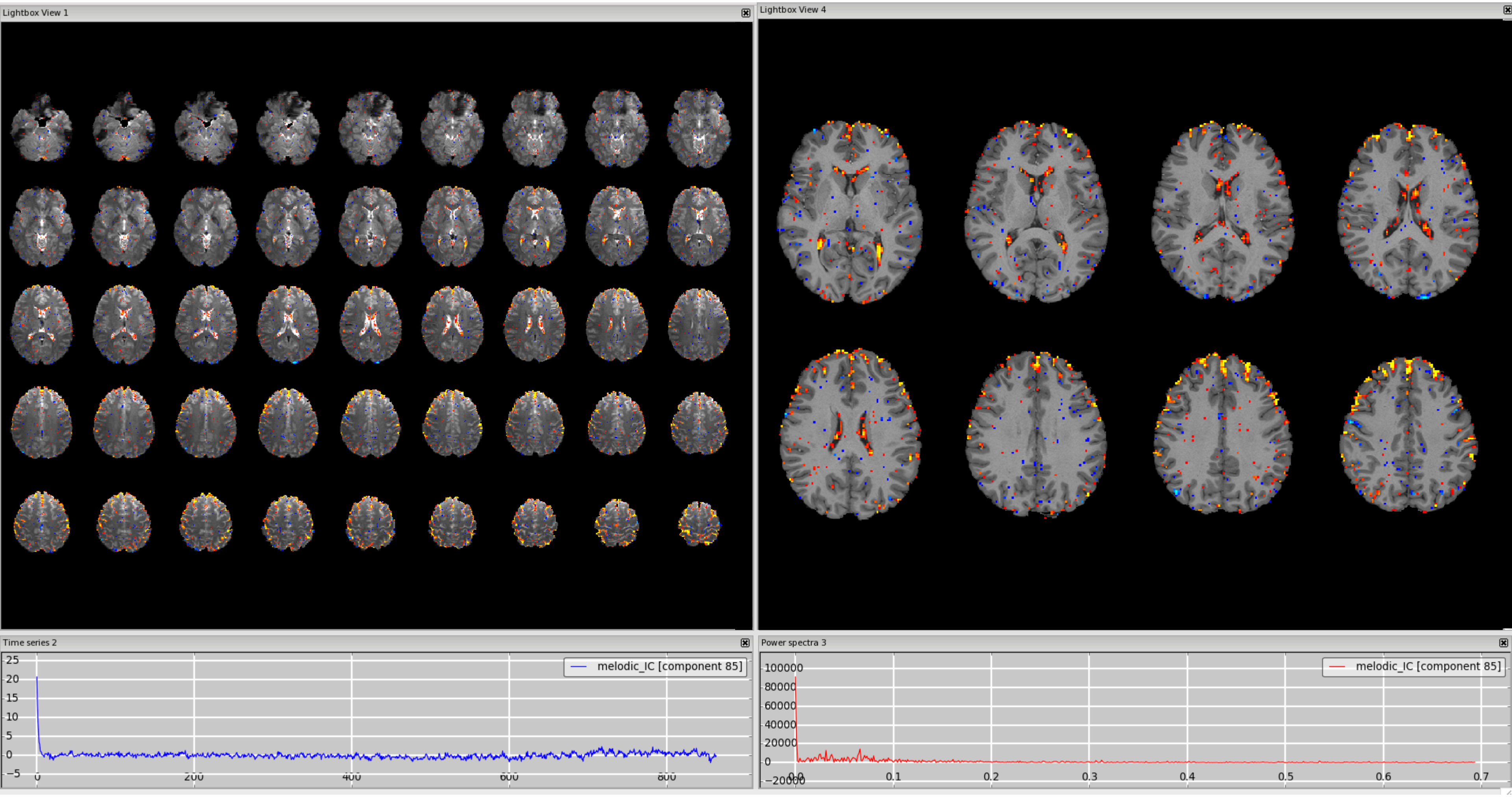
Lightbox View 1
Lightbox View 4
Time series 2
melodic_IC [component 85]
25
20
15
10
5
0
−5
0
200
400
600
800
Power spectra 3
melodic_IC [component 85]
100000
80000
60000
40000
20000
0
−20000
0.0
0.1
0.2
0.3
0.4
0.5
0.6
0.7

Fig S21

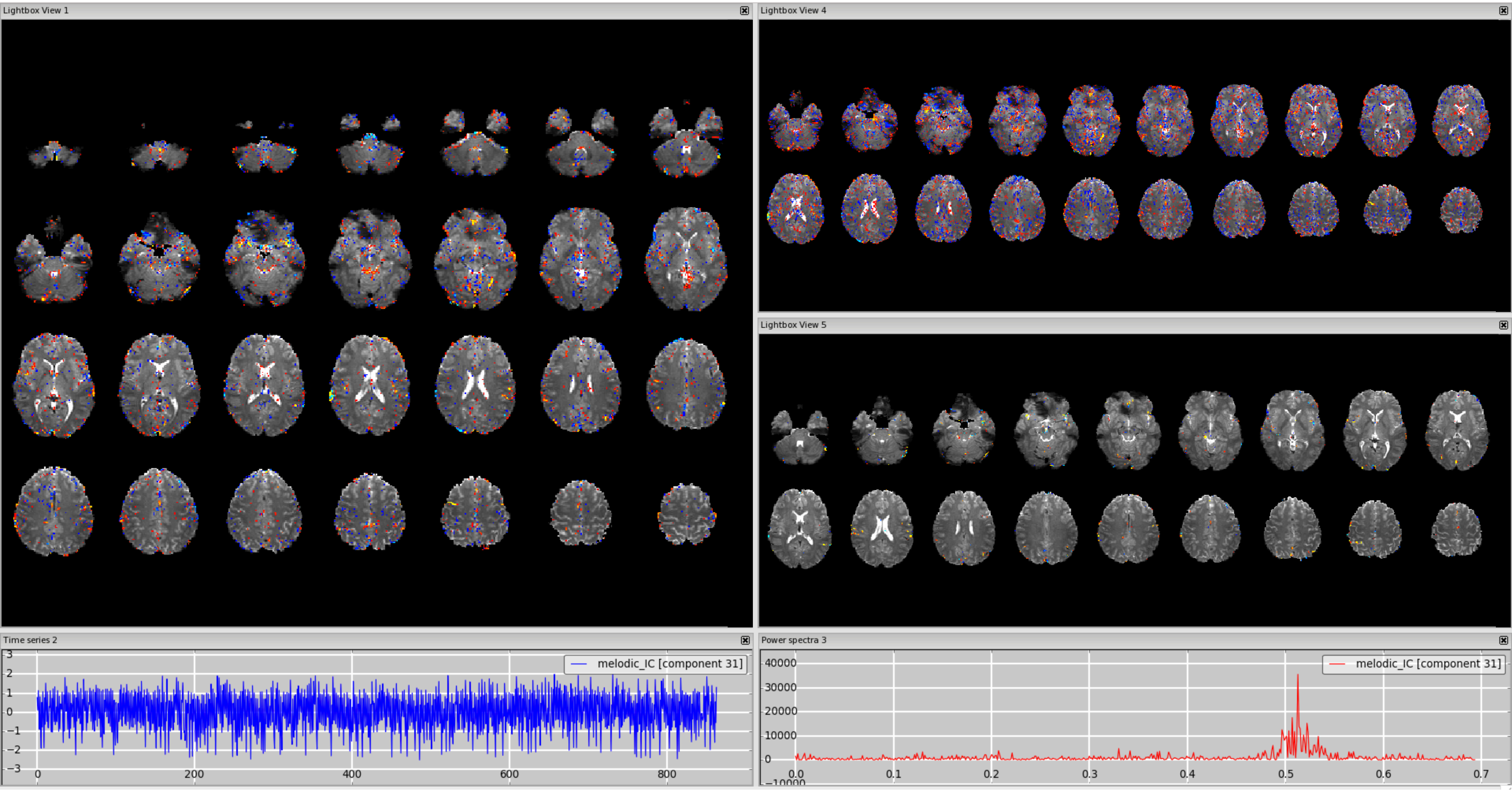
Lightbox View 1
Lightbox View 4
Lightbox View 5
Time series 2
melodic_IC [component 31]
3
2
1
0
−1
−2
−3
0
200
400
600
800
Power spectra 3
melodic_IC [component 31]
40000
30000
20000
10000
0
−10000
0.0
0.1
0.2
0.3
0.4
0.5
0.6
0.7

Fig S22

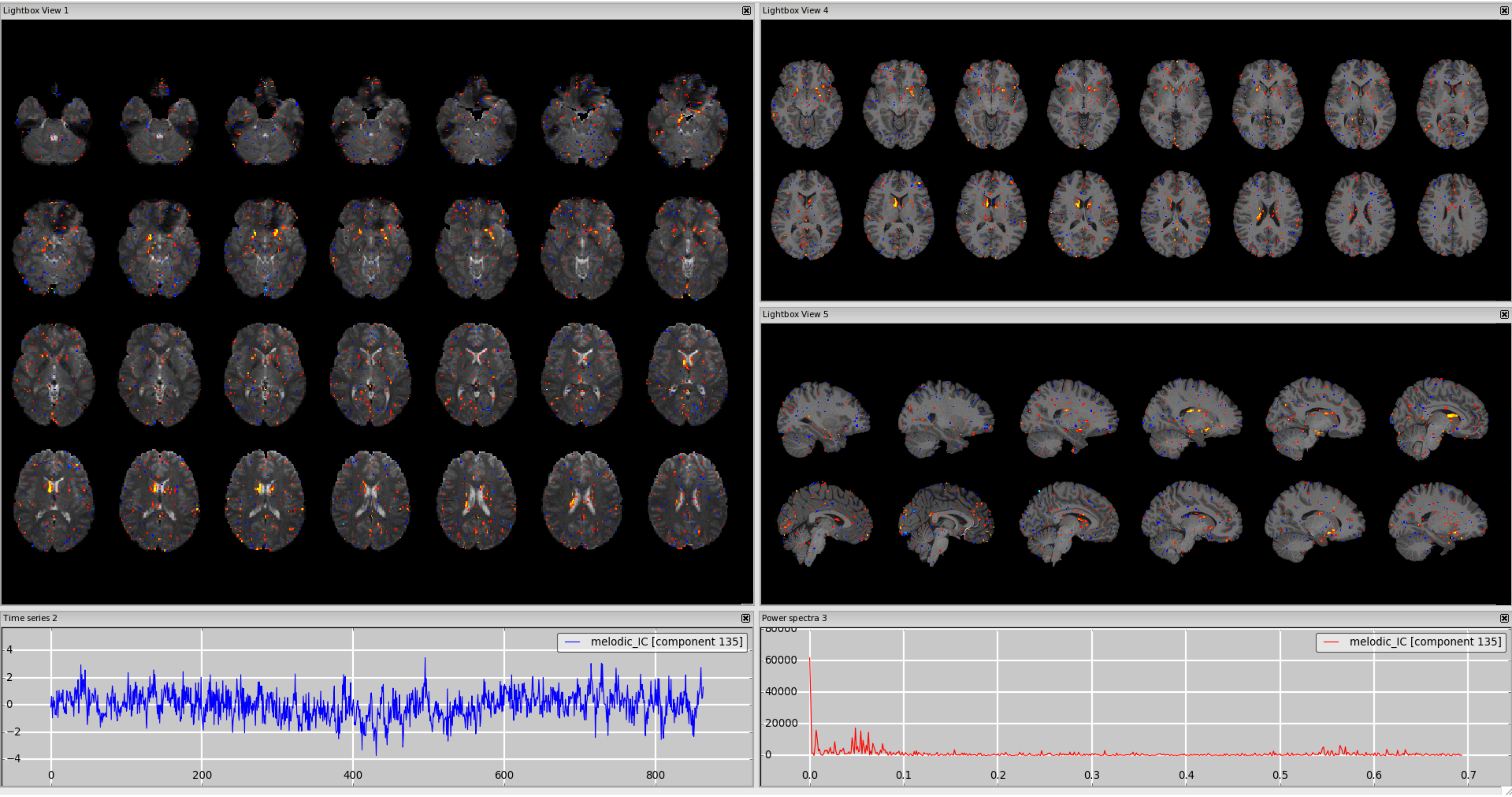
Lightbox View 1
Lightbox View 4
Lightbox View 5
Time series 2
melodic_IC [component 135]
4
2
0
-2
-4
0
200
400
600
800
Power spectra 3
melodic_IC [component 135]
60000
40000
20000
0
0.0
0.1
0.2
0.3
0.4
0.5
0.6
0.7

Fig S23

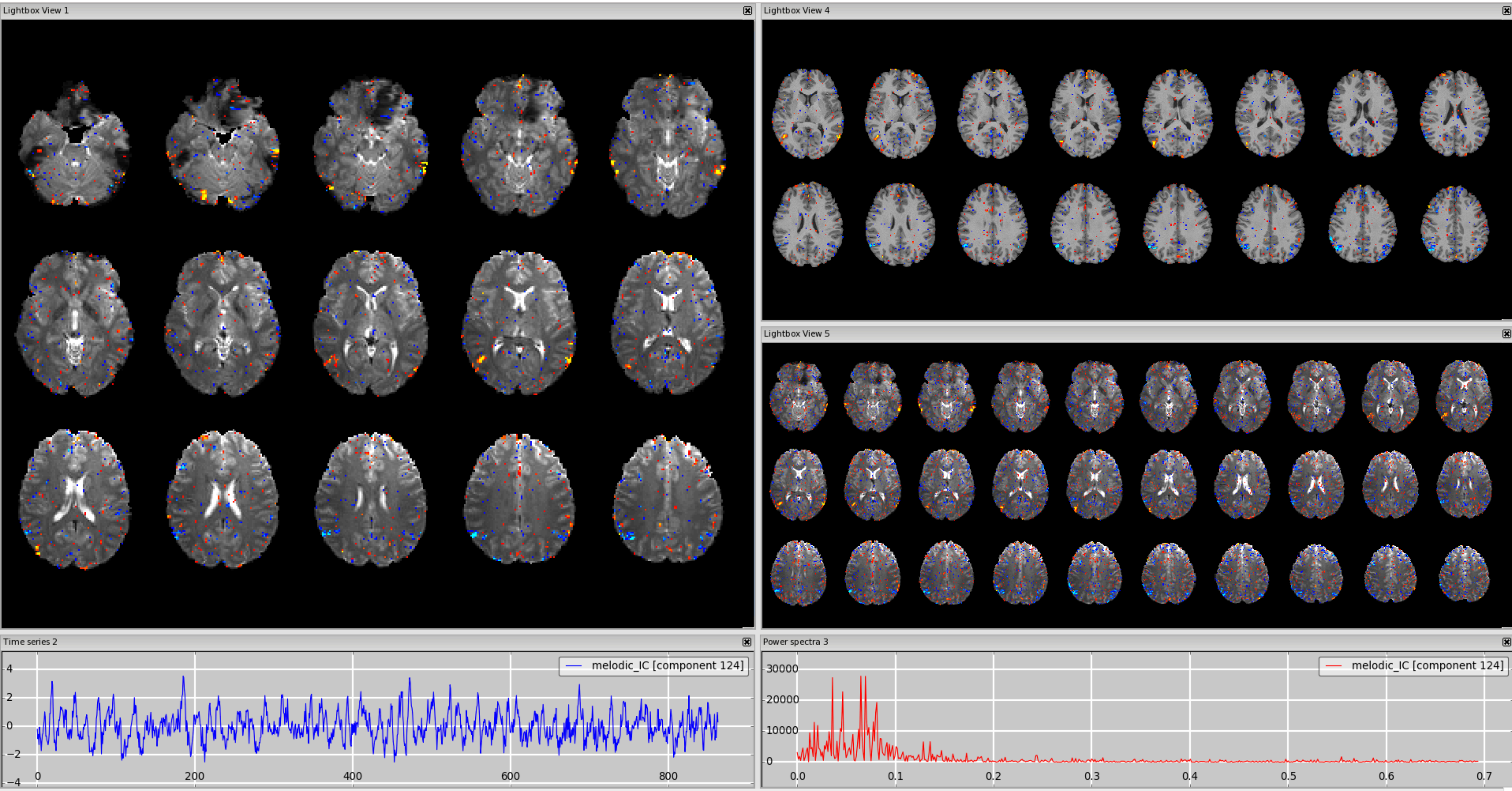
Lightbox View 1
Lightbox View 4
Lightbox View 5
Time series 2
melodic_IC [component 124]
4
2
0
-2
-4
0
200
400
600
800
Power spectra 3
melodic_IC [component 124]
30000
20000
10000
0
0.0
0.1
0.2
0.3
0.4
0.5
0.6
0.7

Fig S24
