# Supplementary material for: Hand classification of fMRI ICA noise components
Source: Neuroimage. 2017 Jul 1;154:188–205. doi: 10.1016/j.neuroimage.2016.12.036 (PMC5489418; doi:10.1016/j.neuroimage.2016.12.036)
Supplement: Supplementary file 3 — Supplementary material [file mmc3.pdf]

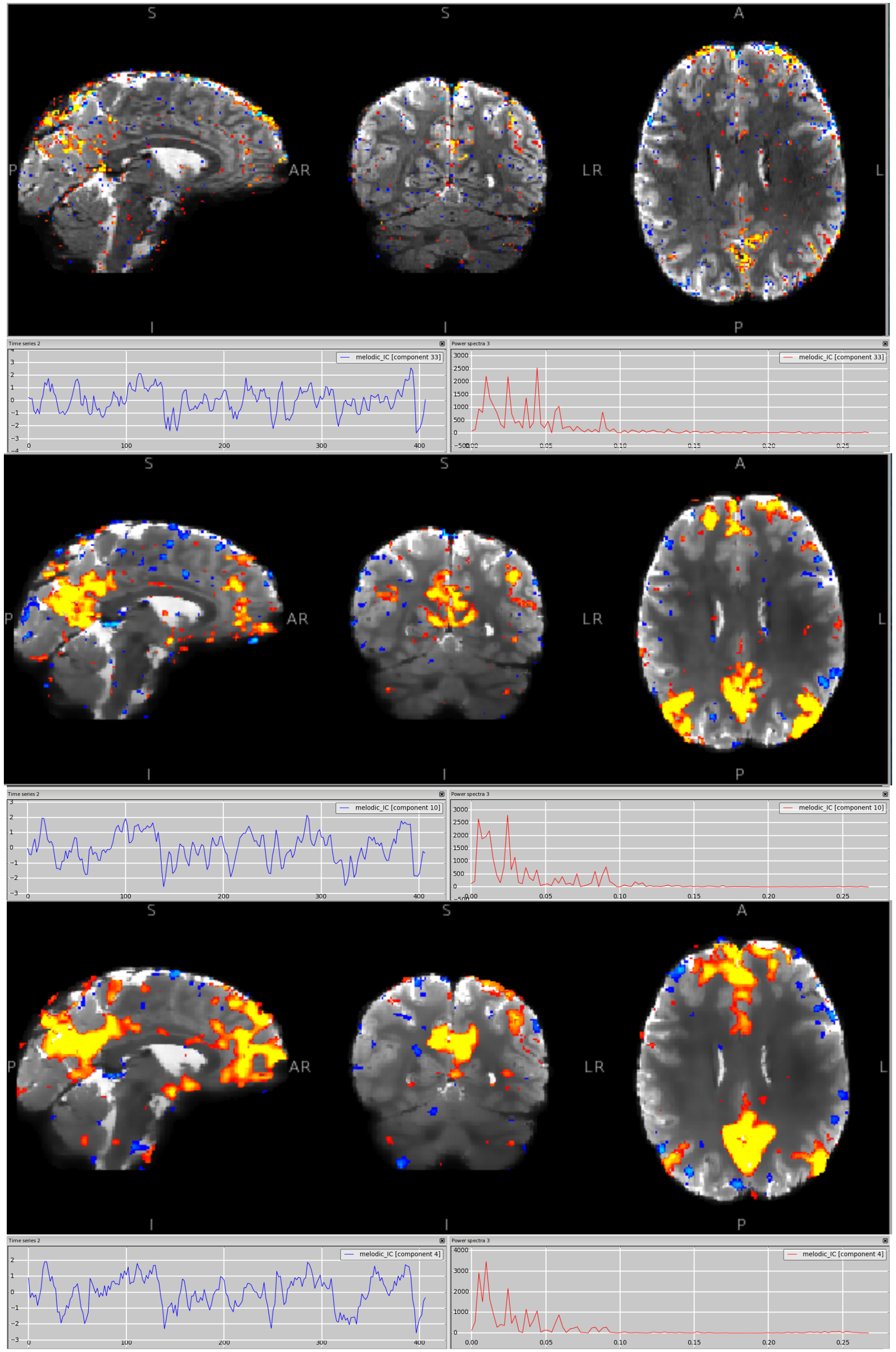
S
S
A
P
AR
LR
L
I
I
P
melodic_IC [component 33]
melodic_IC [component 33]
melodic_IC [component 10]
melodic_IC [component 10]
melodic_IC [component 4]
melodic_IC [component 4]

Fig S25

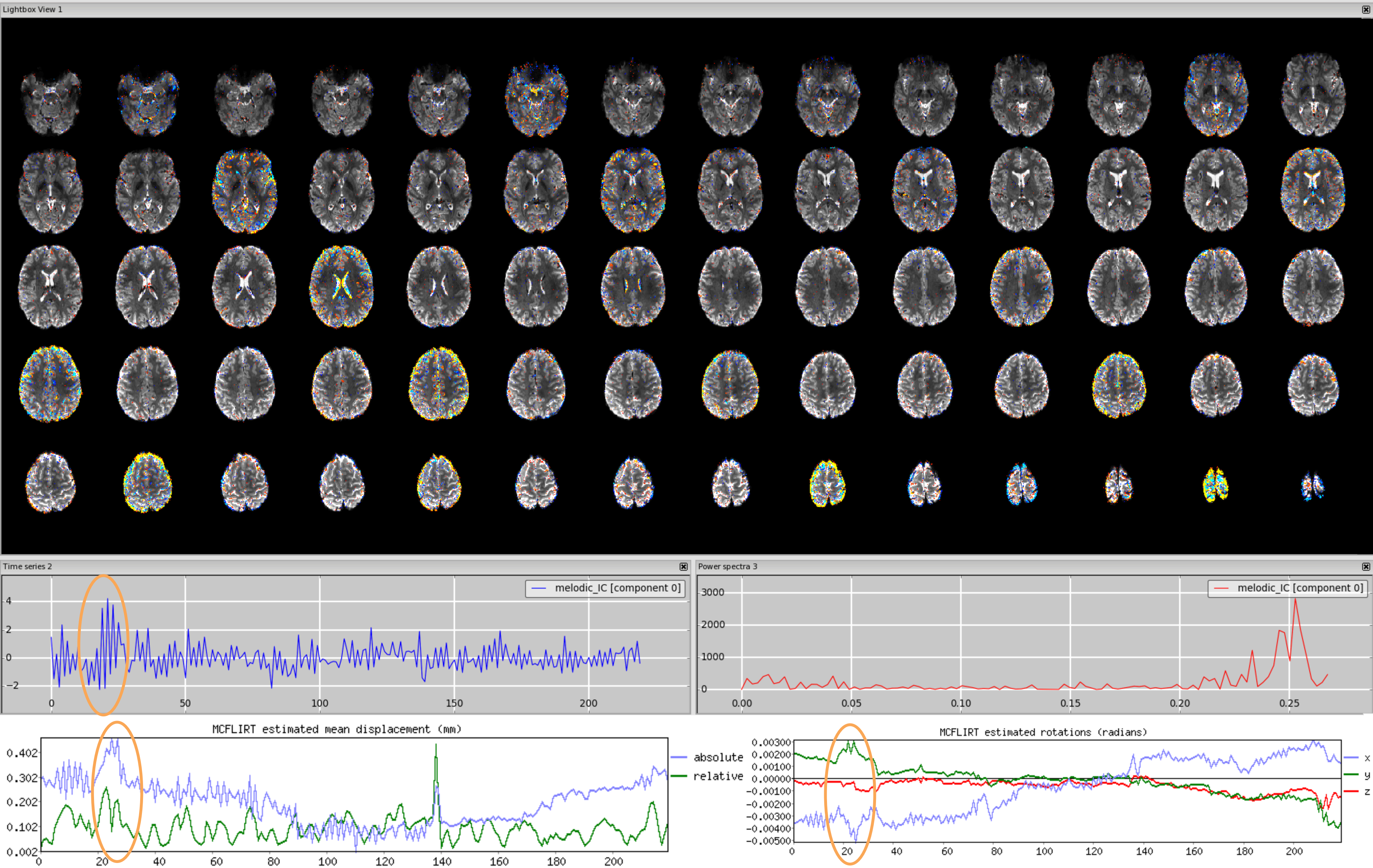
Lightbox View 1
Time series 2
melodic_IC [component 0]
Power spectra 3
melodic_IC [component 0]
MCFLIRT estimated mean displacement (mm)
absolute
relative
MCFLIRT estimated rotations (radians)
x
y
z

Fig S26

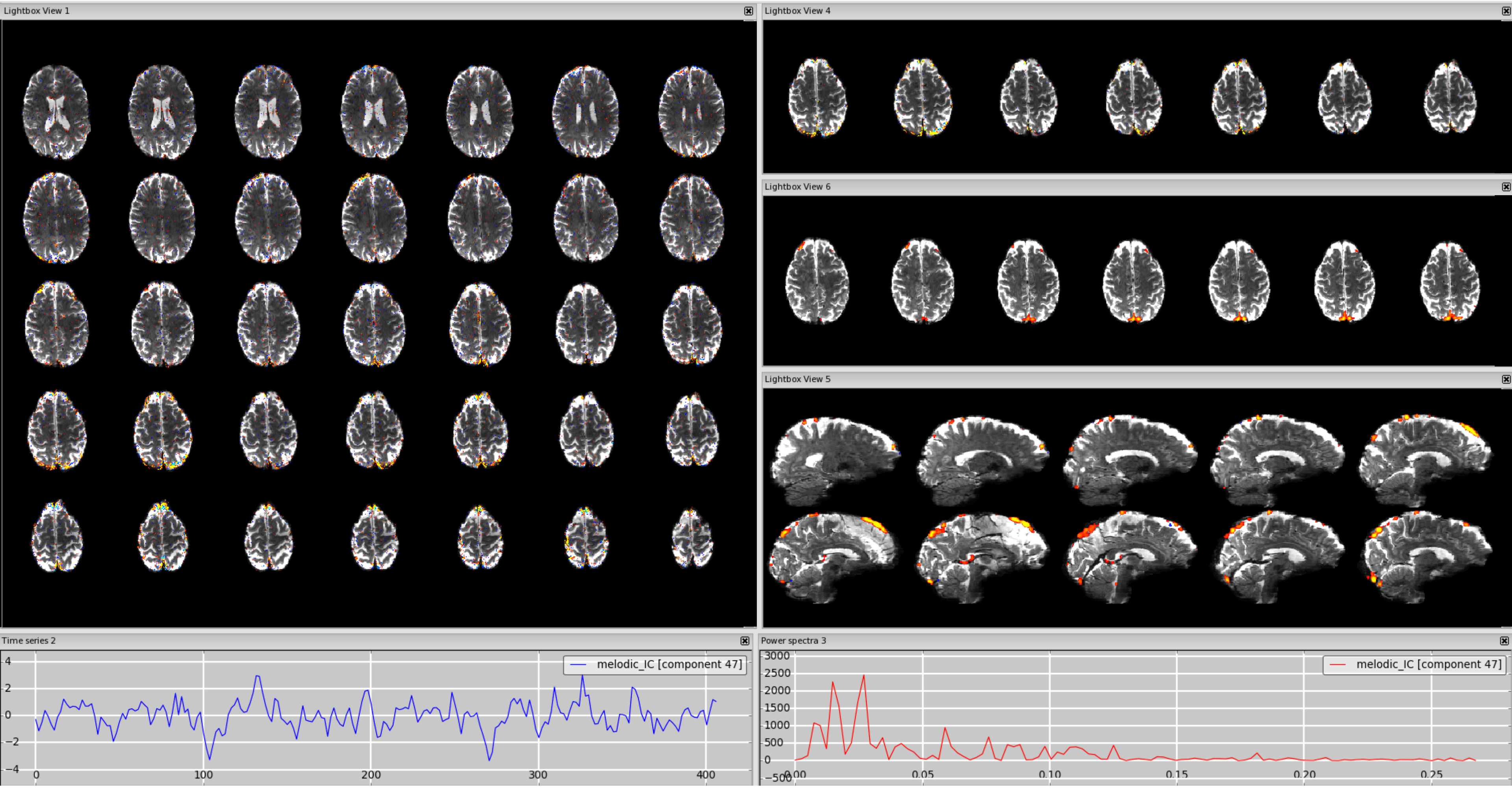
Lightbox View 1
Lightbox View 4
Lightbox View 6
Lightbox View 5
Time series 2
melodic_IC [component 47]
4
2
0
−2
−4
0
100
200
300
400
Power spectra 3
melodic_IC [component 47]
3000
2500
2000
1500
1000
500
0
−500
0.00
0.05
0.10
0.15
0.20
0.25

Fig S27

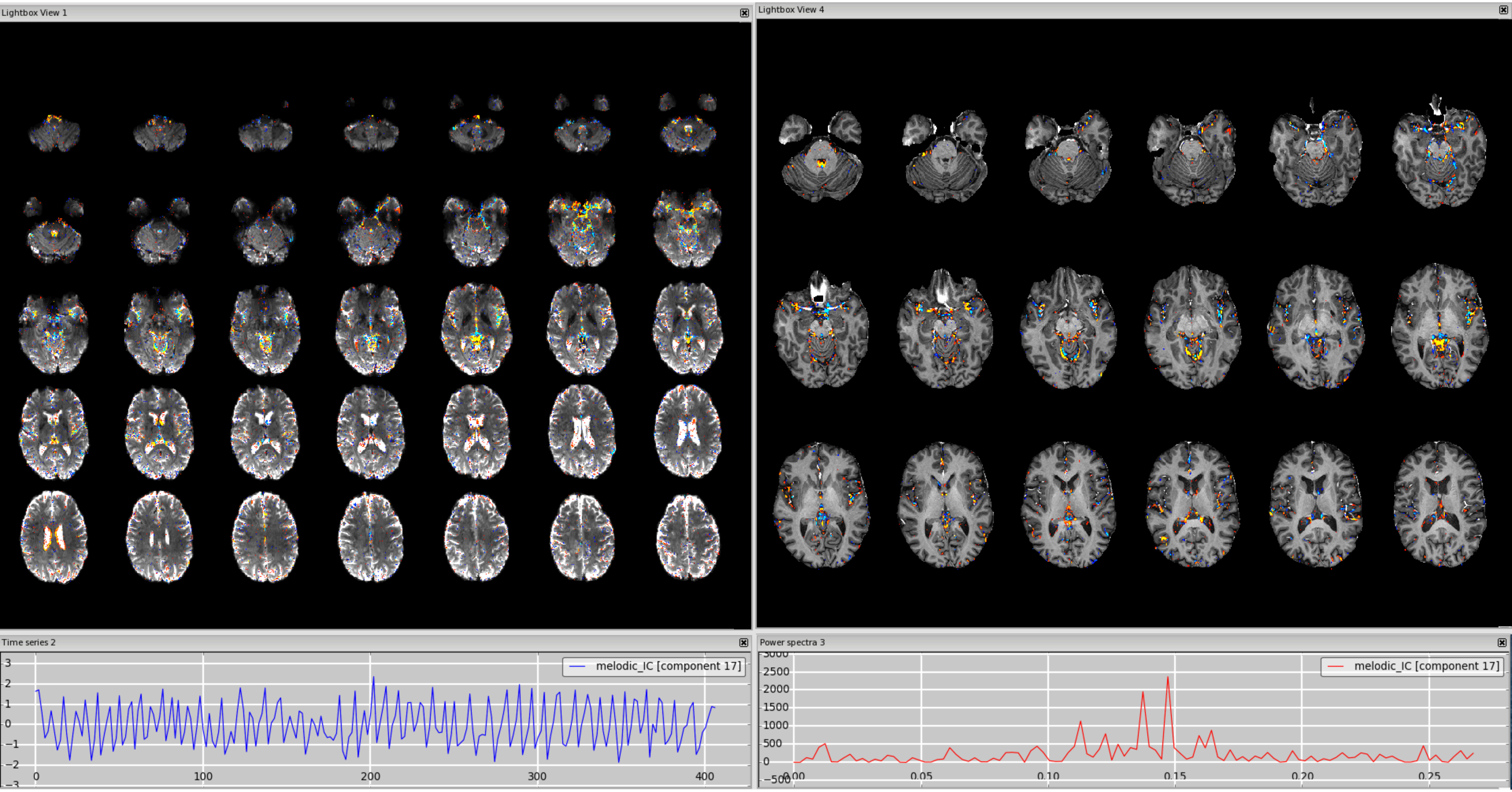
Lightbox View 1
Lightbox View 4
Time series 2
melodic_IC [component 17]
Power spectra 3
melodic_IC [component 17]

Fig S28

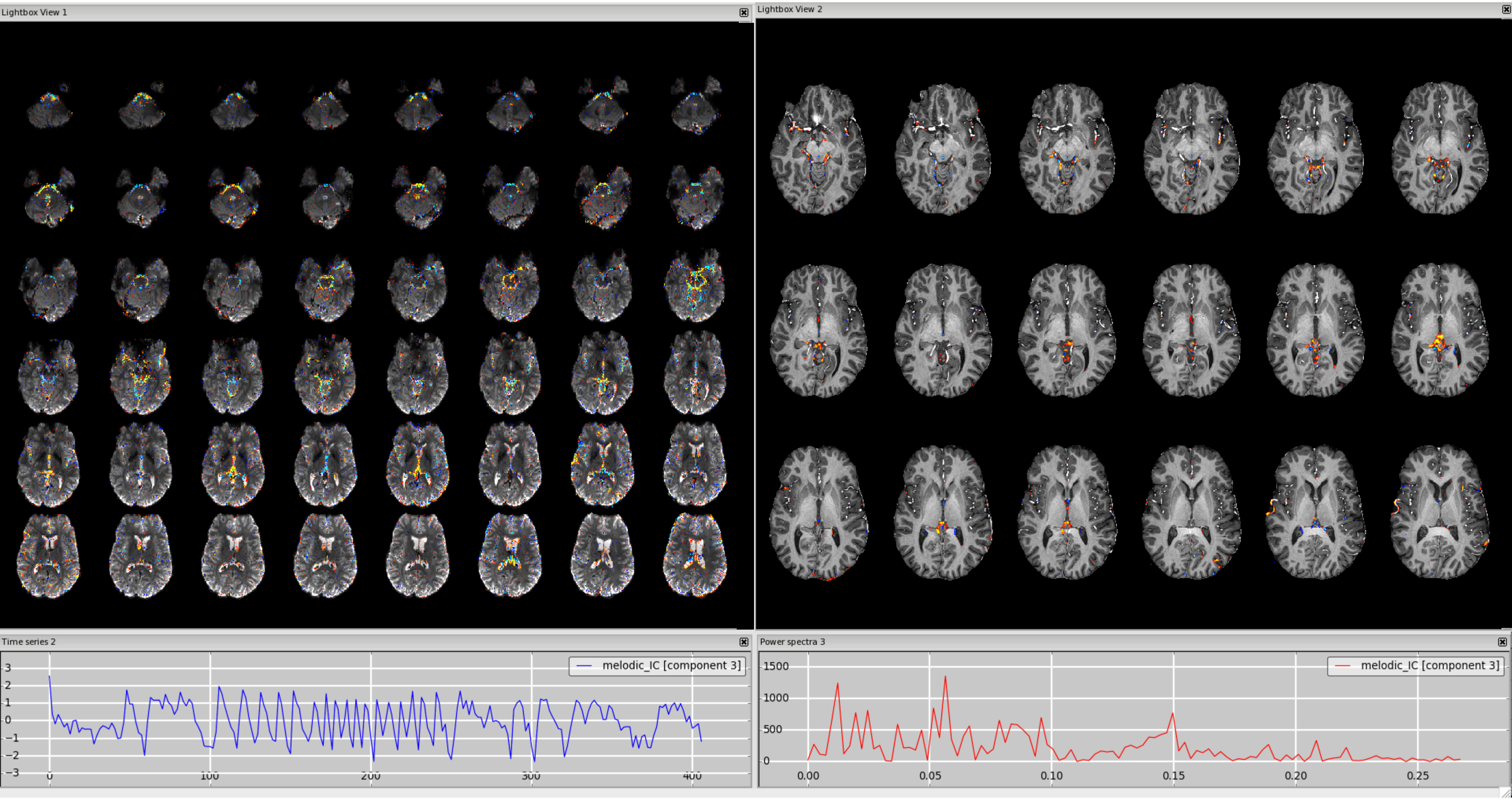

Lightbox View 1
Lightbox View 2
Time series 2
melodic_IC [component 3]
3
2
1
0
−1
−2
−3
0
100
200
300
400
Power spectra 3
melodic_IC [component 3]
1500
1000
500
0
0.00
0.05
0.10
0.15
0.20
0.25

Fig S29

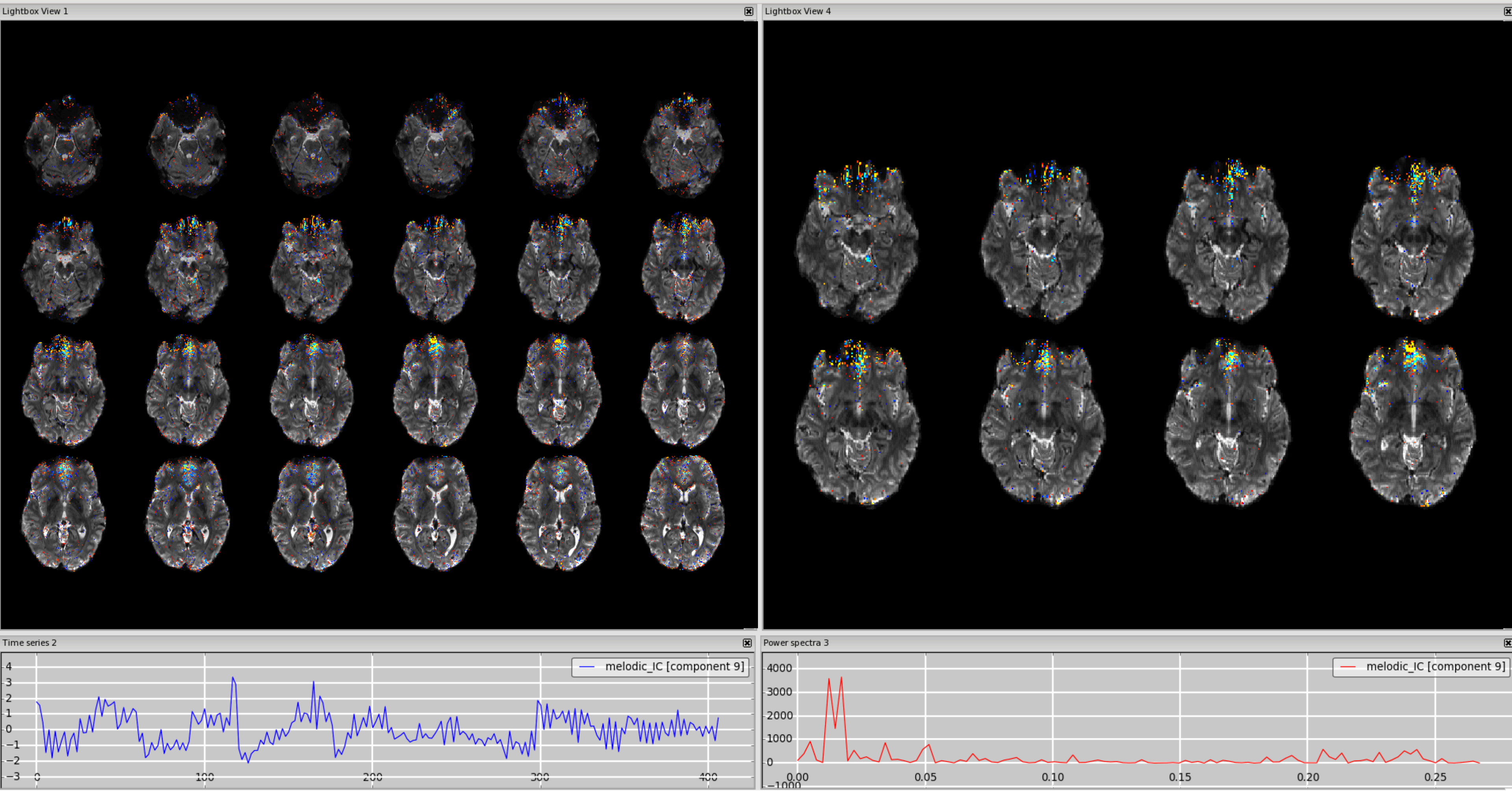
Lightbox View 1
Lightbox View 4
Time series 2
melodic_IC [component 9]
4
3
2
1
0
−1
−2
−3
0
100
200
300
400
Power spectra 3
melodic_IC [component 9]
4000
3000
2000
1000
0
−1000
0.00
0.05
0.10
0.15
0.20
0.25

Fig S30

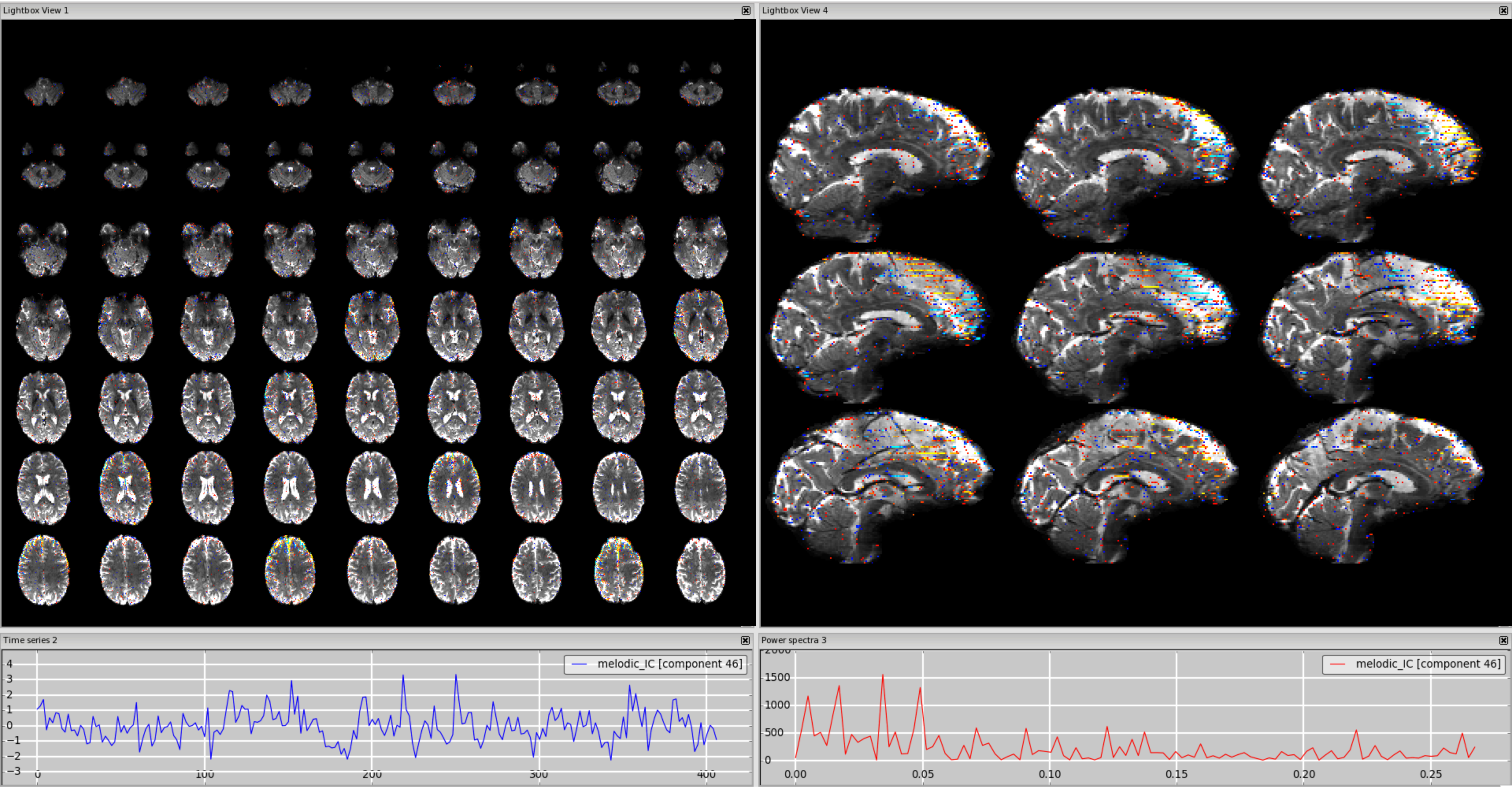
Lightbox View 1
Lightbox View 4
Time series 2
melodic_IC [component 46]
Power spectra 3
melodic_IC [component 46]

Fig S31

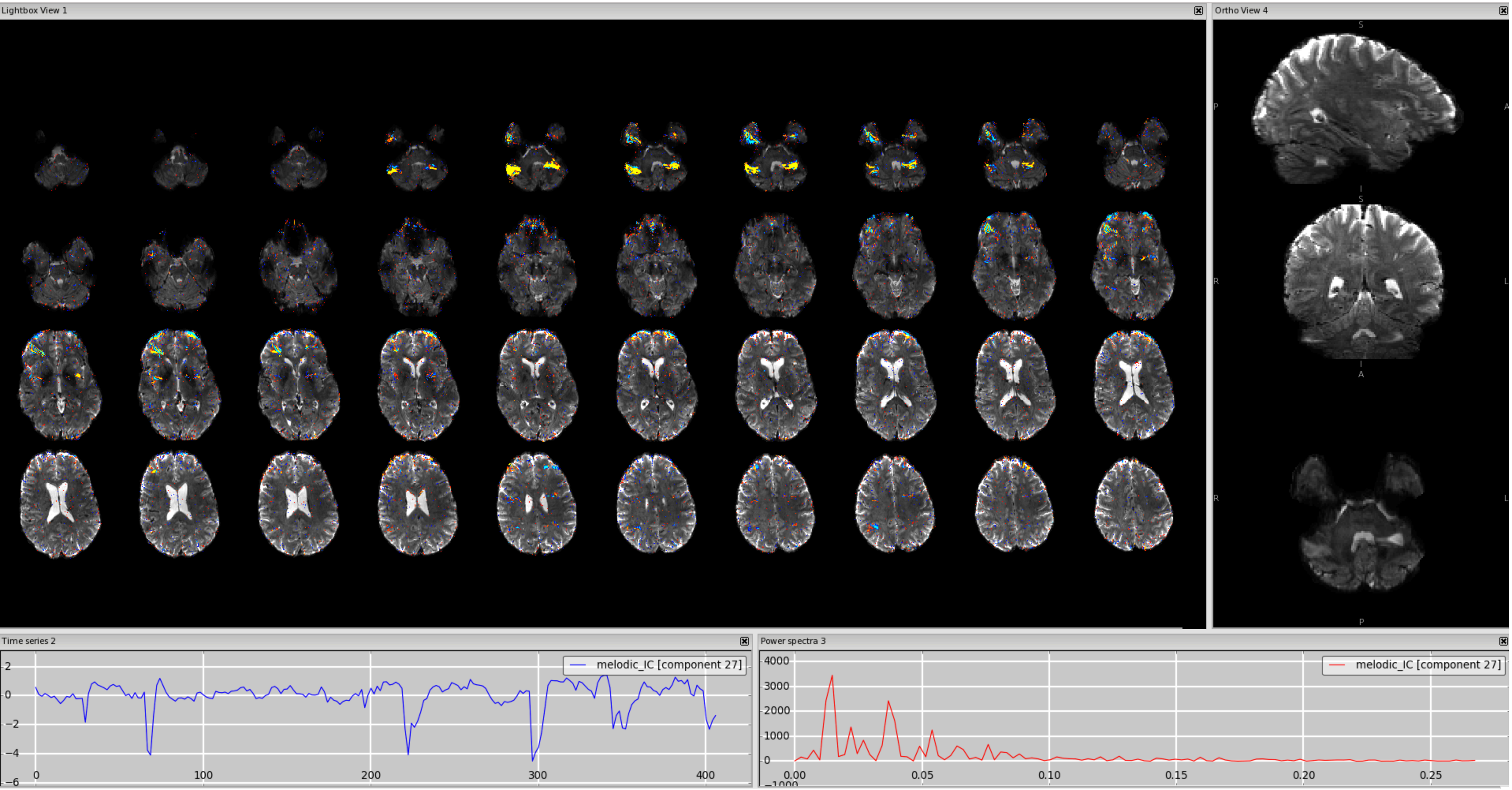

Lightbox View 1
Ortho View 4
Time series 2
melodic_IC [component 27]
Power spectra 3
melodic_IC [component 27]

Fig S32

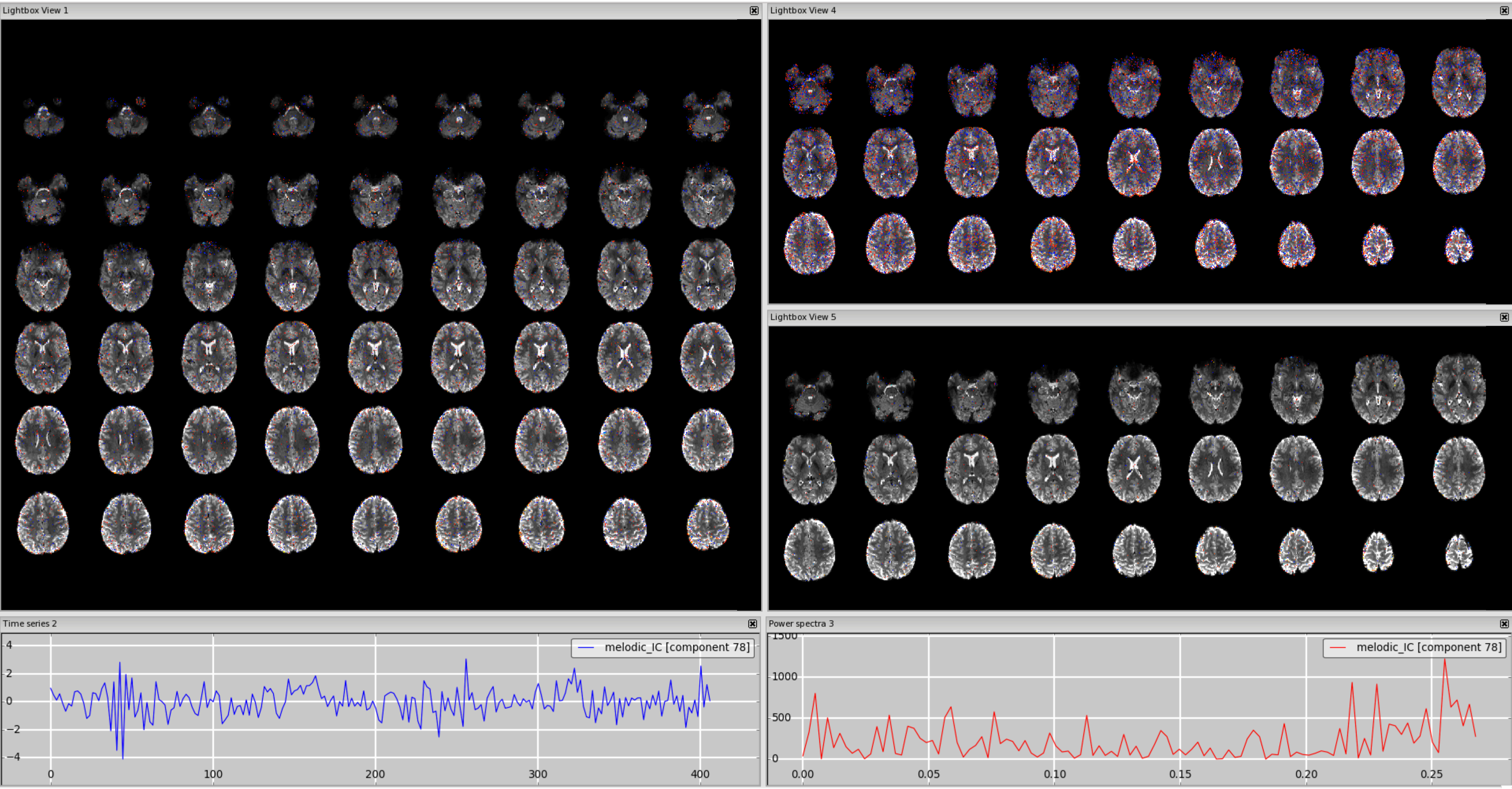
Lightbox View 1
Lightbox View 4
Lightbox View 5
Time series 2
melodic_IC [component 78]
4
2
0
−2
−4
0
100
200
300
400
Power spectra 3
melodic_IC [component 78]
1500
1000
500
0
0.00
0.05
0.10
0.15
0.20
0.25

Fig S33

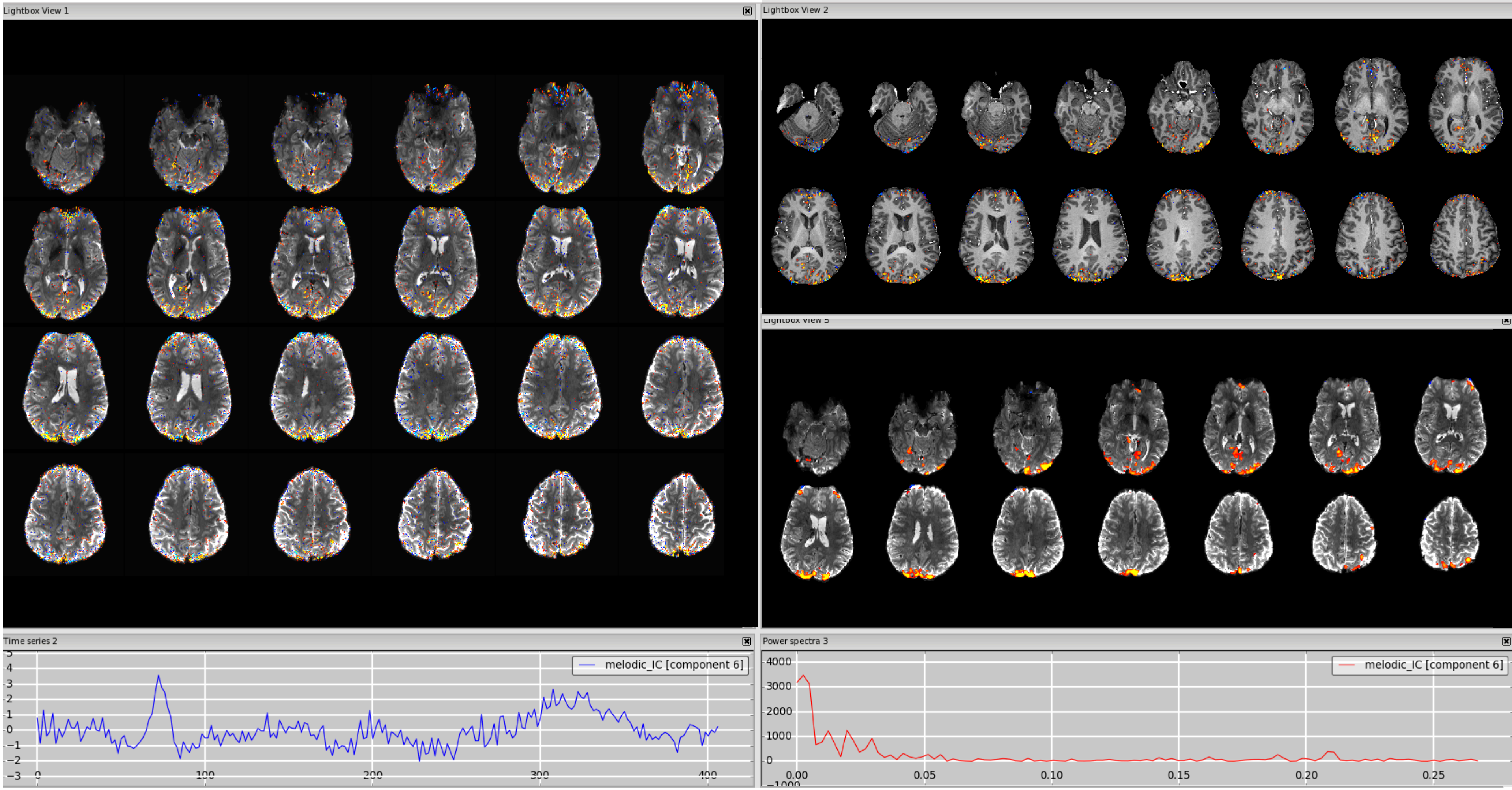

Lightbox View 1
Lightbox View 2
Time series 2
melodic_IC [component 6]
4
3
2
1
0
−1
−2
−3
0
100
200
300
400
Power spectra 3
melodic_IC [component 6]
4000
3000
2000
1000
0
0.00
0.05
0.10
0.15
0.20
0.25

Fig S34

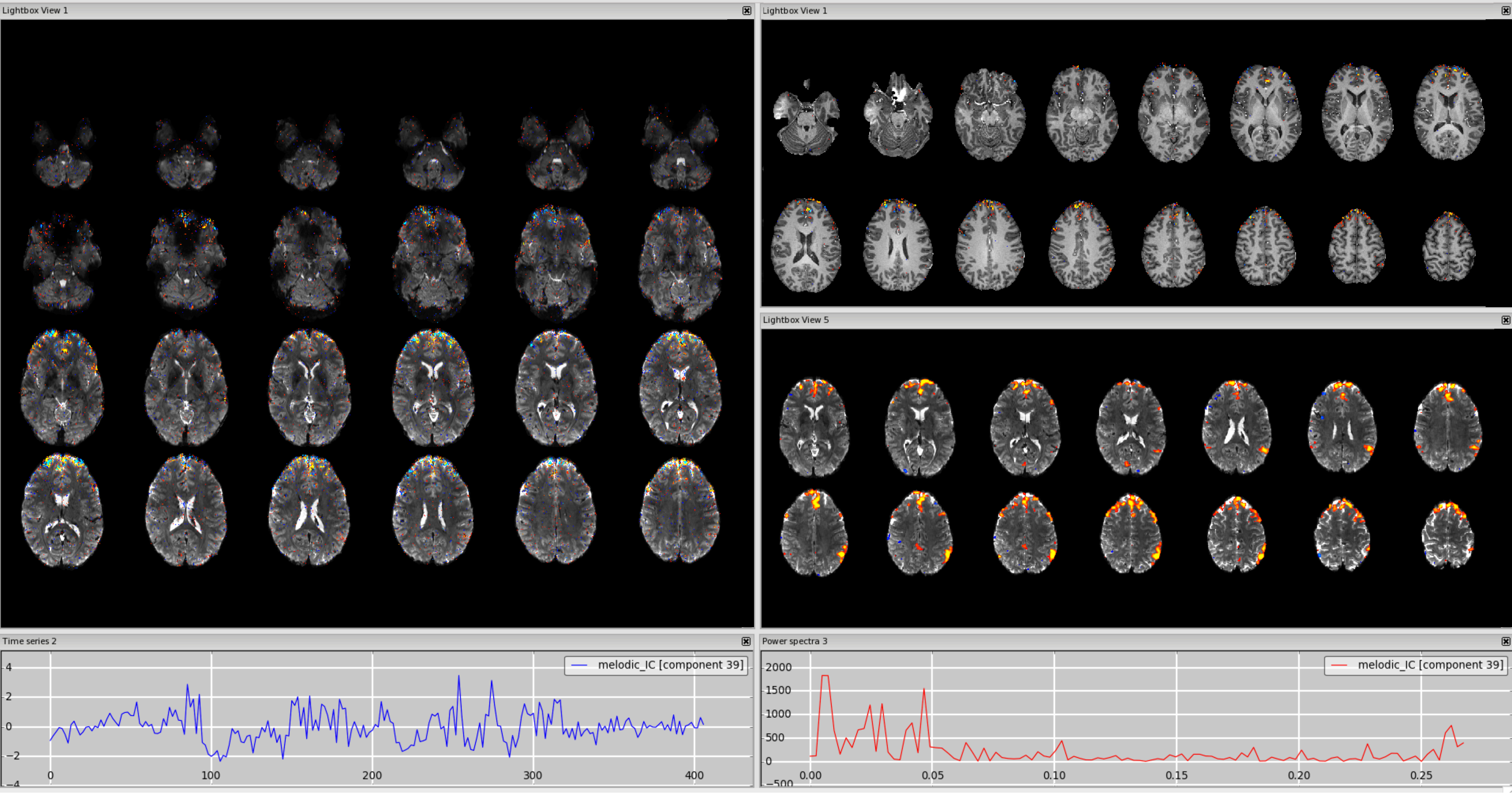
Lightbox View 1
Lightbox View 1
Lightbox View 5
Time series 2
melodic_IC [component 39]
4
2
0
−2
−4
0
100
200
300
400
Power spectra 3
melodic_IC [component 39]
2000
1500
1000
500
0
−500
0.00
0.05
0.10
0.15
0.20
0.25

Fig S35
