# Supplementary material for: Hand classification of fMRI ICA noise components
Source: Neuroimage. 2017 Jul 1;154:188–205. doi: 10.1016/j.neuroimage.2016.12.036 (PMC5489418; doi:10.1016/j.neuroimage.2016.12.036)
Supplement: Supplementary file 5 — Supplementary material [file mmc5.docx]

**Supplementary figure captions**

Figure S1. Examples of tools for components visualisation. a) FSLeyes (a replacement for FSLview that will be part of the next upcoming FSL version) provides standard view setup for hand classification of components (spatial map, time series and power spectrum, components list with a range of predefined classification labels). The user can then customise the view, as in the other examples presented in this paper. b) Connectome Workbench allows display of cortical surface maps of ICA components. The HCP provides standard view setups for each dataset, to visualize the ICA components.

Figure S2. Signal [standard 3T]. An example of signal component showing the Default Mode Network (DMN). The time series (left plot) does not contain sudden jumps and the power spectrum (right plot) is predominantly low frequency. The change of plans and the use of a structural image (e.g. high-resolution T1) as underlay can help evaluating whether the clusters are localised in the GM (right panel). Threshold z=2.3.

Figure S3. Motion artefact [standard 3T]. The spatial map presents the typical ring at the edge of the brain and the time series contains a sudden jump in correspondence to sudden head movement, as visible in the realignment profiles (highlighted in the orange circles).

Figure S4. Veins [standard 3T]. The sagittal sinus is better visible in sagittal plane, with a structural image as underlay (top-right panel). Changing the threshold (in this case from z=2 to z=4) helps checking that the peak is outside the brain (bottom-right panel). The power spectrum (right plot) is not very distinctive, as it contains some low-frequency power.

Figure S5. Arteries [standard 3T]. The middle cerebral artery runs close to the insula, so a structural image as underlay can help localising the vessels. Changing the threshold (in this case from z=2 to z=2.5) helps checking that the peaks are not in the GM (right panel). Note that the power spectrum is not as distinctive as in figure 4, due to aliasing when using longer TR (right plot).

Figure S6. Cerebrospinal fluid pulsation [standard 3T]. The spatial pattern overlaps the ventricles and the cortical CSF, and it is better localised overlaid onto a structural image (right panel). Given that the data have been spatially smoothed, changing the threshold (in this case from z=2.3 to z=3), helps checking that the peaks are in the CSF (right panel).

Figure S7. Fluctuations in subependymal (and transmedullary) veins [standard 3T]. Because the data is smoothed, the spatial pattern overlaps the white matter. It is better localised overlaid onto a structural image and at a higher threshold (in this case from z=2 to z=3) (right panel).

Figure S8. Susceptibility artefacts [standard 3T]. Localised on the EPI in areas of signal drop, due mainly to air-tissue interfaces. The use of a higher threshold (in this case from z=2.3 to z=4) makes it possible to verify that the peak is in the region of signal drop (right panels).

Figure S9. MRI related artefact [standard 3T]. Artefact generated by pulse sequence timing instabilities. The spatial pattern (with clusters present only on two consecutive slices and not following the GM), and the time series (left plot) are not physiologically meaningful.

Figure S10. Unclassified noise [standard 3T]. This component contains multiple different sources of noise, like motion (identifiable from the ring-shaped positive and negative clusters at the brain edge), CSF (with clusters in the ventricles, as visible in the top-right panel, overlaid on top of a structural image), susceptibility artefact, visible in areas of EPI signal drop.

Figure S11. Unknown [standard 3T]. In this example, the component contains clearly some neural-related signal (posterior cingulate - precuneus), but also some artefacts, possibly of vascular origin, especially visible in the sagittal plane (top-right panel).

Figure S12. Unknown [standard 3T]. This component does not look clearly neural-related, but might contain some signal (partial overlap with the GM, low-frequency peak), even if not distinguishable from noise as in figure S11. Careful inspection at different thresholds (from z=2.3 to z=1 in the top-right panel or to z=4 in the bottom-right panel), also with the underlay of structural image, is a good practice to help determine that the component does not belong to other categories.

Figure S13. Signal [HCP]. Example of signal component showing the Default Mode Network (DMN). The time series (left plot) is relatively smooth and the power spectrum (right plot) is clearly low frequency. Looking at different planes and underlying a structural image (T1-w in this case) can help evaluating the localization of the clusters (right panel). Threshold z=2.3.

Figure S14. Motion artefact [HCP]. The spatial map presents the typical ring at the edge of the brain, and even if the time series doesn’t show spikes or jumps, the spatial patters is usually enough to classify this example as a motion artefact. Threshold z=2.3

Figure S15. Veins [HCP]. The sagittal sinus is visible in the axial plane, though the view in the other planes can help the visualization (right panels). With a higher threshold (right panels) it typically results in a single centered peak in the axial view, as well as when smoothing the spatial map (bottom-right panel). Threshold z=2.3 (left panel) and z=6 (right panels).

Figure S16. Arteries [HCP]. The presence of the areas of circle of Willis in the spatial map together with clusters nearby the insula can help to recognize the cardiac origin of the component. Moreover, underlying a structural image can help to exclude the localization in GM (right panel). Threshold z=2.3.

Figure S17. Cerebrospinal fluid pulsation [HCP]. The spatial pattern overlaps the fourth ventricle, the cisterna magna and the aqueduct of Sylvius and some cortical CSF. Moreover the time series is not physiologically meaningful. Localisation can be helped by the overlaid onto a structural image (right panel). Threshold z=2.3. In this component a contribution from the arteries is also present (see Figure S16).

Figure S18. Fluctuations in subependymal (and transmedullary) veins [HCP]. The spatial maps overlap the WM-CSF boundary, better localised overlaid onto a structural image (top-right panel) and smoothing the data (bottom-right panel). Threshold z=2.3.

Figure S19. Susceptibility artefacts [HCP]. The spatial pattern is mainly localised in correspondence of the areas of signal drop in EPI. Increasing the threshold (in this case from z=2.3 to z=5, right panel) makes it possible to verify that the peak is in the region of signal drop.

Figure S20. Multiband artefact [HCP]. In this example, this type of artefact is not straightforward to recognise in the axial view. However, it can be seen as alternating stripes in the coronal or sagittal view (right top and bottom panels, respectively). Threshold z=2.3.

Figure S21. MRI related artifact [HCP]. This artifact is due to the initial T1 saturation effect in the CSF at the start of the sequence. As a consequence, it appears as an initial spike in the time series (left plot) and the spatial pattern overlaps the ventricles and cortical CSF (threshold z=2.3). This component would not have been present if more initial scans had been deleted before preprocessing.

Figure S22. Unclassified noise [HCP]. The component has a predominantly high frequency spectrum (right plot), a noisy time series (left plot) and a scattered spatial pattern. Neither decreasing the threshold (in this case from z=2.3 to z=1.5, top-right panel) or increasing it (in this case from z=2.3 to z=5, bottom-right panel) shows any GM cluster that would likely have neural origin.

Figure S23. Unknown [HCP]. In this example, the component clearly contains some neural-related signal in the basal ganglia, but also some noise (e.g. physiological noise). The power spectrum (right panel) shows both a peak at low frequencies and some smaller peaks at higher frequencies, and the time series is irregular and contains some spikes. Threshold z=2.3.

Figure S24. Unknown [HCP]. In this example there is no clear presence of signal or noise. The spatial pattern shows some clusters in the GM, but not clearly attributable to one of the most typical RSN. Using a structural image as background (top-right panel) or trying to change the threshold (from z=2.3 to z=1,8 in this case) can help to check that the peaks are properly located in GM and can help to determine that the component does not belong to other categories.

Figure S25. Signal [7T]. Example of signal components showing the Default Mode Network (DMN) with spatial smoothing of different extent performed in the preprocessing phase. From top to bottom: no spatial smoothing, smoothing with Gaussian kernel FWHM=3mm and with FWHM=5 mm, respectively. The time series (left plots) are relatively smooth and the power spectra (right plots) are predominantly low frequency. Threshold z=2.3.

Figure S26. Motion artefact [7T]. The spatial map presents the typical ring at the edge of the brain and the time series contains peaks in good correspondence with the realignment profiles (highlighted in the orange circles). Threshold z=2.3. The fact that the spatial pattern is visible in alternating slices suggests that in this component motion artefact is combined with multiband artefact (see Figure S31).

Figure S27. Veins [7T]. The sagittal sinus is visible in the axial plane, though the sagittal plane can help the visualization (bottom-right panel). Increasing the threshold (in this case from z=2.3 to z=3, top right panel) or smoothing the spatial maps of the component (middle-right panel), helps checking that the peak is outside the brain (top-right panel), excluding that the component contains neural-related signal from the occipital lobe (e.g. primary visual RSN).

Figure S28. Arteries [7T]. The presence of the circle of Willis in the spatial map can help to recognise the cardiac origin of the component. Moreover, underlying a structural image (right panel) can help to exclude the presence of neural-related signal in the GM (insula). Threshold z=2.3.

Figure S29. Cerebrospinal fluid and major vessels pulsation [7T]. This is an example of interaction between acquisition, CSF pulsation and major vessels. Unlike previous examples of this type of artefact, the spatial pattern of this component does not include the ventricles and the cortical CSF completely, but the peaks are localised mainly in correspondence of the CSF nearby vessels. The contribution from arteries is also present, as they are both related to physiological pulsation. The pattern of signal in alternating slices suggests an interaction with the acquisition sequence (multiband). Localisation can be helped by the overlaid onto a structural image (right panel). Threshold z=2.3.

Figure S30. Susceptibility artefacts [7T]. The spatial pattern is mainly localised in correspondence of the areas of signal drop in EPI. Increasing the threshold makes it possible to verify that the peak is in the region of signal drop (right panel). Threshold z=2.3 (left panel) and z=3 (right panel).

Figure S31. Multiband artefact [7T]. Not easily recognizable in the axial view, it can be seen as alternating stripes in the other planes (e.g., in the sagittal plane, right panel). Threshold z=2.3.

Figure S32. MRI related artifact [7T]. Example of a 7T-related artifact. The spatial map (left panel) shows strong peaks located in the cerebellum. These peaks correspond to BOLD signal hyperintense foci (right panel) caused by the fat from the eyes due to uneven fat suppression. Some spikes can be detected in the time series (left plot).

Figure S33. Unclassified noise [7T]. The present component has a scattered spatial pattern (left panel) and quite broad frequency (right plot). Some spikes are present in the time series (left plot) and the power spectrum contains a strong high frequency peak (right plot). Neither decreasing the threshold (in this case from z=2.3 to z=1.5, top-right panel) nor increasing it (in this case from z=2.3 to z=3, bottom-right panel) shows any GM cluster that would likely have neural origin.

Figure S34. Unknown [7T]. In this example, the component contains some neural-related signal in the occipital lobe, but also some noise, mainly in frontal areas, nearby EPI signal dropout. The power spectrum is predominantly low frequency but the time series is quite irregular. The occipital clusters are the ones mainly visible after smoothing the spatial map (bottom-right panel). Threshold z=2.3.

Figure S35. Unknown [7T] This component in some ways looks like neuronal-related signal spatially, particularly after smoothing (bottom-right panel, sigma=5mm), but the mixture of positive and negative voxels in the unsmoothed map is more complicated. Also the time series is not as cleanly oscillatory in the way seen in clean pure signal components.
